# An Explainable 2D-QSAR Machine Learning Approach for Predicting COX-2 Inhibitory Activity Using Molecular Fingerprints

**DOI:** 10.3390/ph19050698

**Published:** 2026-04-29

**Authors:** Mebarka Ouassaf, Bader Y. Alhatlani

**Affiliations:** 1 Group of Computational and Medicinal Chemistry, LMCE Laboratory, University of Biskra, Biskra 07000, Algeria; 2 Unit of Scientific Research, Applied College, Qassim University, Buraydah 52571, Saudi Arabia

**Keywords:** COX-2 inhibitors, machine learning, Random Forest, QSAR, Morgan fingerprints, SHAP, explainable artificial intelligence, virtual screening, drug discovery, cancer

## Abstract

**Background/Objectives:** Cyclooxygenase-2 (COX-2) is a well-established target in the development of anti-inflammatory drugs due to its central role in mediating inflammation. The identification of novel COX-2 inhibitors remains a key focus in pharmaceutical research. This study aimed to develop a robust and interpretable machine learning framework to predict COX-2 inhibitory activity and support virtual screening efforts. **Methods:** A curated dataset of 2052 compounds was obtained from the ChEMBL database. Molecular structures were encoded using Morgan fingerprints derived from SMILES representations. Several machine learning algorithms were trained and evaluated, including ensemble-based methods. Model performance was assessed using internal validation and external test sets. Robustness was further evaluated through Y-randomization tests. Model interpretability was investigated using SHAP (SHapley Additive exPlanations) analysis to identify key structural features contributing to activity. **Results:** Among the evaluated models, ensemble methods demonstrated superior predictive performance, with the Random Forest algorithm providing the most consistent and reliable results across validation and external datasets. Y-randomization confirmed that the model predictions were not due to chance correlations. SHAP analysis revealed that the most influential features corresponded to chemically meaningful substructures aligned with known COX-2 pharmacophore characteristics. The final optimized model was successfully deployed as a publicly accessible web application for real-time prediction using SMILES input. **Conclusions:** This study demonstrates the effectiveness of explainable machine learning approaches in predicting COX-2 inhibitory activity. The developed framework provides a reliable and interpretable tool for accelerating COX-2 inhibitor discovery and facilitating virtual screening in drug development.

## 1. Introduction

Cyclooxygenase (COX) enzymes play a central role in inflammatory pathways by catalyzing the conversion of arachidonic acid into prostaglandins [1]. Among them, cyclooxygenase-2 (COX-2) is an inducible isoform primarily associated with inflammation, pain, and the progression of various cancers, whereas COX-1 is constitutively expressed and involved in essential physiological functions such as gastric mucosal protection and platelet homeostasis [2]. This functional distinction led to the development of selective COX-2 inhibitors aimed at reducing the gastrointestinal side effects commonly observed with non-selective nonsteroidal anti-inflammatory drugs (NSAIDs) [3].

However, post-marketing clinical and epidemiological studies revealed that several selective COX-2 inhibitors, including rofecoxib and valdecoxib, are associated with increased cardiovascular risks, such as myocardial infarction and stroke, leading to their withdrawal or restricted use [4]. Consequently, there is a persistent need for the discovery of novel COX-2 inhibitors with improved safety profiles while maintaining therapeutic efficacy.

In addition, most currently known COX-2 inhibitors share closely related chemical scaffolds, limiting structural diversity and constraining further optimization of selectivity and toxicity [5]. Traditional drug discovery pipelines rely on experimental screening of large chemical libraries, a process that is time-consuming, costly, and characterized by high attrition rates [6,7]. These challenges highlight the need for efficient computational strategies capable of prioritizing promising candidates at early stages.

In this context, machine learning (ML) has emerged as a powerful tool for modeling complex structure–activity relationships in cheminformatics [8,9,10]. In particular, fingerprint-based 2D-QSAR approaches provide an effective representation of molecular structures by encoding substructural information into numerical vectors suitable for predictive models [11,12]. However, many high-performing ML models behave as “black boxes”, limiting interpretability and reducing confidence in their predictions [13,14,15].

To address these limitations, the present study proposes an explainable 2D-QSAR machine learning framework for predicting COX-2 inhibitory activity based on Morgan fingerprints. By combining robust predictive performance, external validation, applicability domain analysis, and SHAP-based interpretation, this approach aims to identify chemically meaningful structural features associated with COX-2 inhibition. Furthermore, the developed model is deployed as an accessible web-based application, enabling real-time prediction and facilitating its use in virtual screening and early-stage drug discovery.

## 2. Results

### 2.1. Comparison of Machine Learning Models

In the context of developing a reliable predictive model for COX-2 inhibitory activity, a range of machine learning algorithms were systematically evaluated to identify the most effective approach for distinguishing active compounds from inactive ones. The primary objective was not only to achieve high predictive accuracy but also to ensure balanced performance in terms of precision and recall, thereby minimizing false positives while maintaining sensitivity toward active compounds.

As summarized in Table 1, the evaluated models exhibited varying levels of predictive performance across the validation dataset, reflecting differences in their ability to capture underlying structure–activity relationships. Among the tested algorithms, ensemble-based models demonstrated superior and more stable performance. In particular, the Random Forest model achieved the best overall results, with an accuracy of 0.808 and well-balanced precision (0.833) and recall (0.856) for the active class, resulting in a robust F1-score of 0.807. The relatively high recall indicates that the model is effective in correctly identifying active compounds, while the strong precision suggests a reduced rate of false positives, which is critical in virtual screening applications.

Other models such as MLP and Naïve Bayes showed competitive performance, with accuracy values close to 0.80; however, their performance was less consistent across different evaluation metrics. For instance, although Naïve Bayes achieved a comparable F1-score (0.804), its probabilistic assumptions may limit its ability to fully capture complex nonlinear relationships. In contrast, models such as Decision Tree and Gradient Boosting exhibited comparatively lower performance, likely due to overfitting tendencies (in the case of Decision Tree) or insufficient generalization under the given parameter settings [16,17].

**Figure 1:**
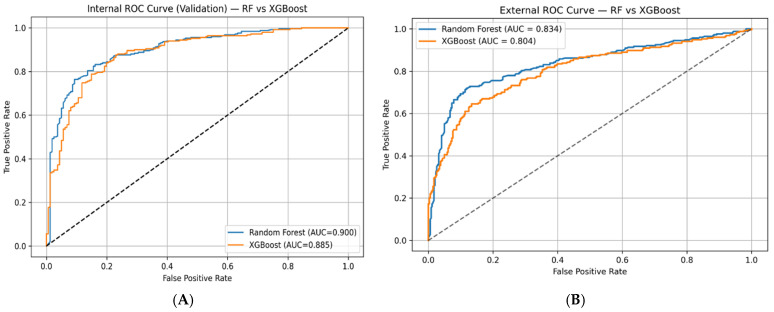
Receiver operating characteristic (ROC) curves of Random Forest and XGBoost models. (**A**) Internal validation ROC curves. (**B**) External validation ROC curves. The dashed diagonal line represents the performance of a random classifier (AUC = 0.5).

To further assess model discriminative power, receiver operating characteristic (ROC) analysis was performed for the top-performing models (Random Forest and XGBoost). As shown in Figure 1A, the Random Forest model achieved a higher area under the curve (AUC = 0.902) compared to XGBoost (AUC = 0.894) on the internal validation set, indicating slightly better separation between active and inactive compounds.

Importantly, external validation results (Figure 1B) confirmed the robustness and generalization capability of the models. The Random Forest model maintained superior performance with an AUC of 0.822, outperforming XGBoost (AUC = 0.790). The observed decrease in AUC from internal to external validation is expected due to the increased chemical diversity of the external dataset; however, the consistent advantage of Random Forest suggests better adaptability to unseen chemical space.

The predicted class labels and probability scores for the validation and external datasets are provided in Supplementary Table S3 to ensure transparency and facilitate further evaluation of model performance.

Based on the combined evaluation of classification metrics and ROC analysis across both internal and external datasets, Random Forest and XGBoost were identified as the top-performing models. Nevertheless, Random Forest demonstrated more consistent and reliable performance, making it the most suitable candidate for subsequent interpretation and deployment in COX-2 activity prediction.

### 2.2. Visualization of Chemical Space Using t-SNE

**Figure 2:**
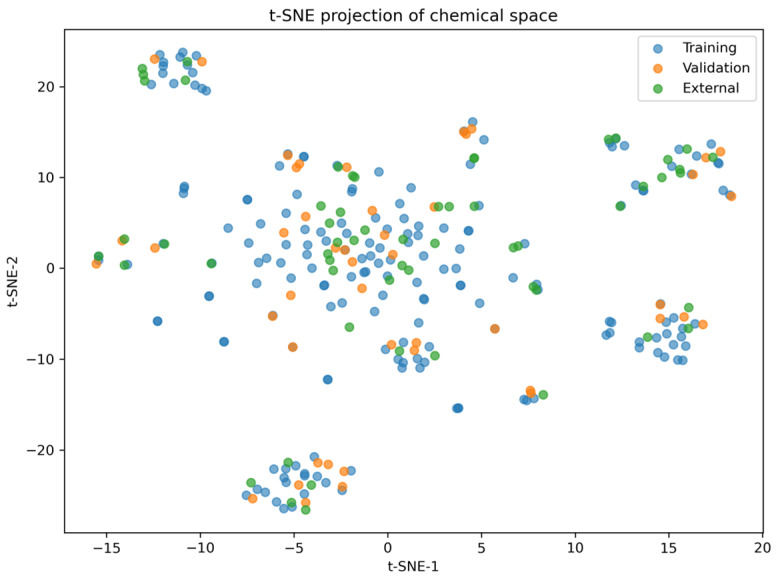
t-SNE projection of the chemical space of the training, validation, and external datasets based on ECFP4 fingerprints. The training and validation sets exhibit substantial overlap, indicating consistent internal data distribution. The external dataset shows a broader distribution with partially overlapping and distinct clusters, reflecting increased structural diversity while remaining partially within the learned chemical space.

To further explore the structural diversity of the datasets, a two-dimensional visualization of the chemical space was performed using t-SNE based on ECFP4 fingerprints. As shown in Figure 2, the training and validation datasets exhibit substantial overlap, confirming the consistency of the internal data split. In contrast, the external dataset displays a broader and partially dispersed distribution, indicating increased structural diversity. Notably, several clusters remain shared between the external and training sets, suggesting that a significant portion of external compounds fall within the learned chemical space. These findings are consistent with the applicability domain analysis and further support the robustness and generalization capability of the developed model, particularly in handling structurally diverse external compounds.

### 2.3. Y-Randomization Analysis of the Final Model

Although the developed model demonstrated strong predictive performance on the validation and external datasets, it is essential to ensure that such performance is not driven by chance correlations, which may arise when working with high-dimensional molecular descriptors such as fingerprint representations. In cheminformatics and QSAR modeling, models can occasionally achieve seemingly good performance without capturing true structure–activity relationships.

To address this concern, a Y-randomization (permutation) test was conducted using the Random Forest model. In this procedure, the activity labels were randomly shuffled while keeping the molecular descriptors unchanged, and the model was retrained using the same configuration. This process was repeated over multiple iterations (*n* = 20), and the resulting performance metrics were recorded.

The original (non-randomized) model achieved (Table 2) an accuracy of 0.867 and an AUC of 0.931, whereas models trained on randomized labels showed substantially lower performance, with mean accuracy of approximately 0.623 and mean AUC of approximately 0.518. These values are close to random classification behavior, as expected when no real relationship exists between structure and activity. The associated *p*-values (0.0476) further indicate that the probability of obtaining the observed model performance by chance is low and statistically significant.

**Figure 3:**
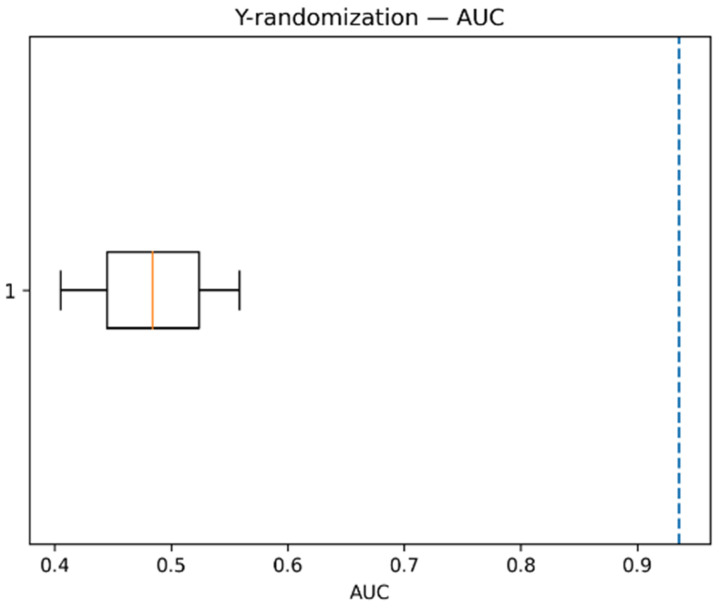
Y-randomization test showing the distribution of AUC values for models trained on randomized labels compared to the original model performance. The boxplot represents the distribution of AUC values from randomized models, where the orange line indicates the median AUC. The blue dashed vertical line represents the AUC of the original (non-randomized) model.

As illustrated in Figure 3, the boxplot represents the distribution of AUC values obtained from the randomized models, while the dashed vertical line indicates the AUC of the original model. The clear separation between the two highlights that the true model performance lies well outside the range of the randomized baseline. The compact distribution of the randomized AUC values around 0.5 reflects random guessing, whereas the markedly higher AUC of the original model confirms that the model has learned meaningful and non-random structure–activity relationships.

Overall, the Y-randomization results confirm that the model captures meaningful structure–activity relationships rather than chance correlations.

### 2.4. Applicability Domain (AD) Results

The applicability domain (AD) analysis was conducted to assess the reliability of model predictions for novel compounds and to determine whether they fall within the chemical space represented by the training dataset [14]. In cheminformatics, predictions are generally considered more reliable when compounds are structurally similar to those used during model development [18].

**Figure 4:**
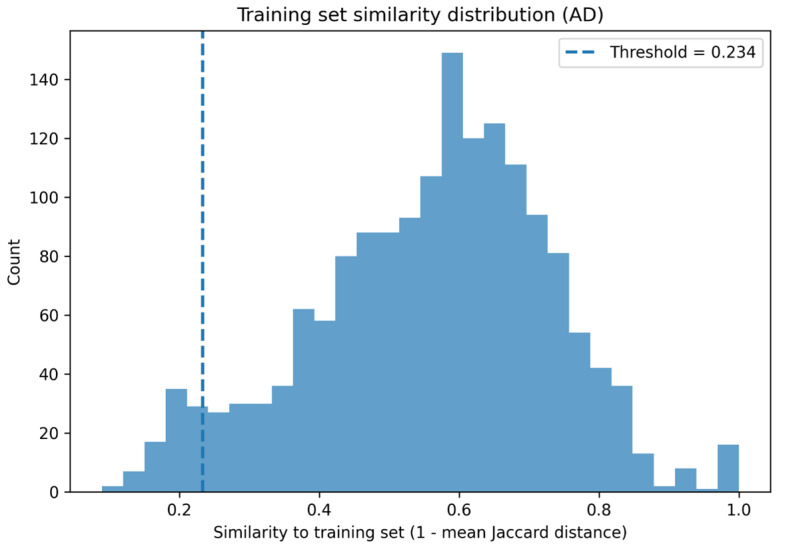
Distribution of Jaccard similarity values within the training set used to define the applicability domain (AD). The dashed vertical line represents the similarity threshold separating compounds inside and outside the model’s chemical space.

A distance-based approach was employed using molecular fingerprint similarity. As shown in Figure 4, the distribution of similarity values within the training set was used to define a threshold separating compounds inside and outside the applicability domain, thereby representing the boundary of the chemical space learned by the model. The histogram illustrates this distribution, where the vertical dashed line indicates the applicability threshold. Compounds located to the right of this threshold are considered within the domain, while those on the left fall outside and may yield less reliable predictions.

**Figure 5:**
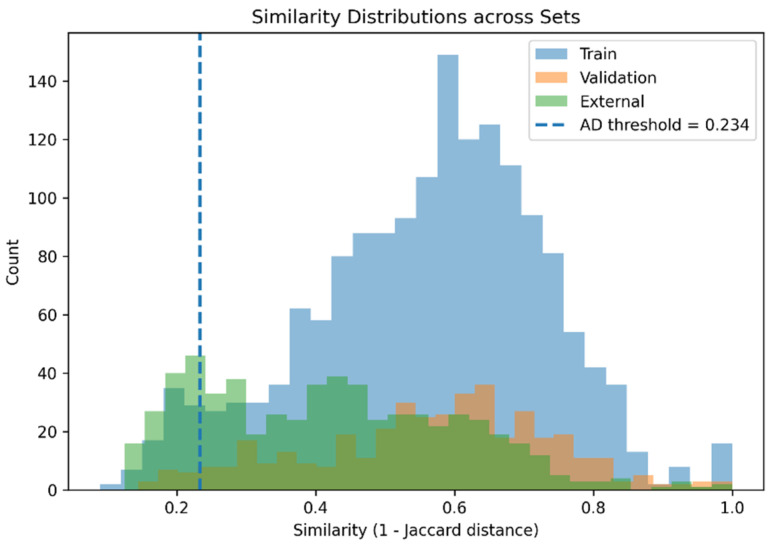
Similarity distributions across training, validation, and external datasets. The overlap between distributions indicates that most compounds fall within the applicability domain, supporting the reliability of model prediction.

Further comparison of similarity distributions across datasets (Figure 5) shows that the majority of validation and external compounds fall within or near the defined applicability domain, indicating substantial overlap with the training chemical space and supporting the validity of the model’s predictions on unseen data.

**Figure 6:**
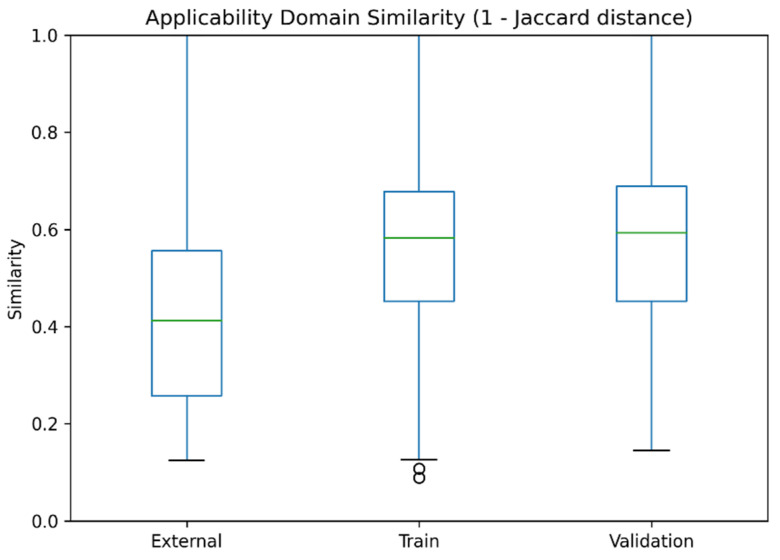
Boxplot of similarity values for training, validation, and external datasets. The box represents the interquartile range (IQR), the green line inside the box indicates the median, and the whiskers represent the range of non-outlier values. Circles denote outliers. Comparable distributions confirm that the datasets share similar chemical space, with slightly lower similarity observed for the external set due to increased structural diversity.

Additionally, the boxplot representation (Figure 6) provides a comparative overview of similarity distributions across the training, validation, and external datasets. The training set exhibits a median similarity of approximately 0.58, with an interquartile range (IQR) from about 0.45 to 0.68. A highly comparable distribution is observed for the validation set, with a median close to 0.60, confirming that validation compounds lie within a similar chemical space.

In contrast, the external dataset shows a lower median similarity of approximately 0.40, with values distributed between roughly 0.25 and 0.55. This shift toward lower similarity reflects increased structural diversity, which is expected for an independent test set. Despite this difference, the distributions still partially overlap, indicating that a substantial portion of external compounds remains within the applicability domain.

Overall, the AD analysis demonstrates that most evaluated compounds fall within the model’s chemical space, ensuring that predictions are reliable and not based on extrapolation beyond the learned domain. This finding further supports the robustness and practical applicability of the developed COX-2 predictive model.

### 2.5. SHAP-Based Model Interpretation

To investigate the contribution of individual structural features encoded as Morgan fingerprint bits, SHAP (SHapley Additive exPlanations) analysis was performed for the Random Forest model [19]. This approach enables quantification of the impact of each substructural feature on model predictions.

**Figure 7:**
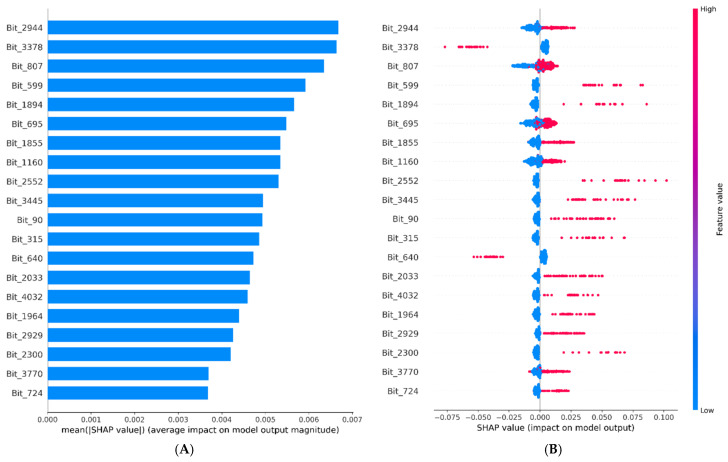
SHAP-based interpretation of the Random Forest model using Morgan fingerprint descriptors. (**A**) Global SHAP importance ranking of fingerprint bits. (**B**) SHAP summary (beeswarm) plot showing the magnitude and direction of feature contributions.

The global SHAP importance ranking (Figure 7A) identified the top 20 fingerprint bits based on their mean absolute SHAP values. These bits represent local atomic environments and molecular fragments contributing to the prediction of COX-2 inhibitory activity. The relatively small differences in importance among the top-ranked features suggest that the model integrates multiple complementary substructures rather than relying on a single dominant pharmacophore.

The SHAP summary plot (Figure 7B) illustrates both the magnitude and direction of feature contributions. In general, the presence of a fingerprint bit (value = 1) is associated with positive SHAP values, indicating an increased probability of COX-2 inhibitory activity, whereas its absence (value = 0) tends to correspond to neutral or negative contributions.

Notably, certain fingerprint bits exhibited variable SHAP distributions across compounds, indicating context-dependent effects. For example, some features contribute positively to specific structural environments while showing weaker or negative effects in others, reflecting the complex interplay between molecular substructures.

**Figure 8:**
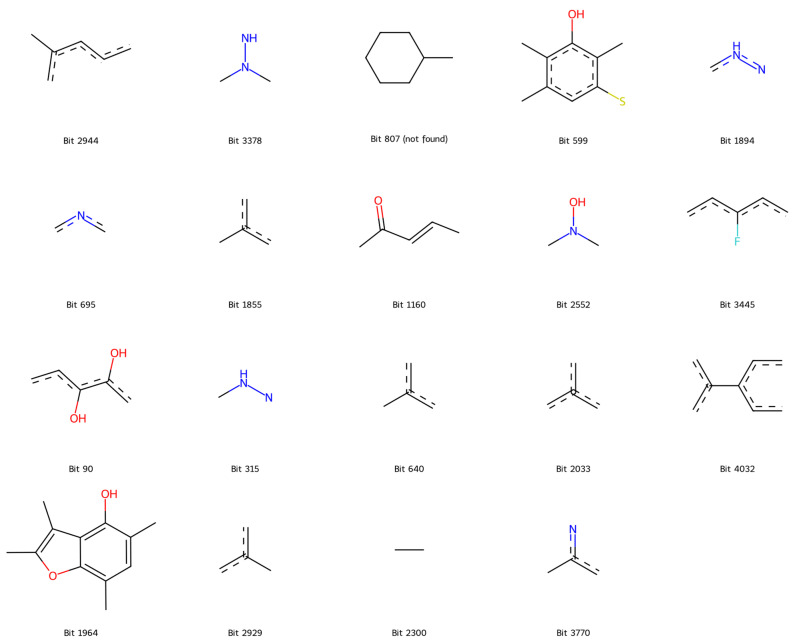
Mapped substructures associated with important fingerprint bits. Atom colors follow standard conventions: oxygen (red), nitrogen (blue), sulfur (yellow), fluorine (light blue), and carbon (gray/black).

To enhance chemical interpretability, the most influential fingerprint bits were mapped to representative molecular substructures (Figure 8). These mapped fragments correspond to chemically meaningful motifs, including aromatic systems, heteroatom-containing groups, and hydrophobic regions, which are consistent with known structural requirements for COX-2 inhibition [20,21,22]. This mapping provides a direct link between model predictions and underlying chemical features, supporting the validity and interpretability of the developed model. To further interpret these findings at the compound level, a case study analysis was conducted using known COX-2 inhibitors.

### 2.6. Model Deployment and Web Interface

**Figure 9:**
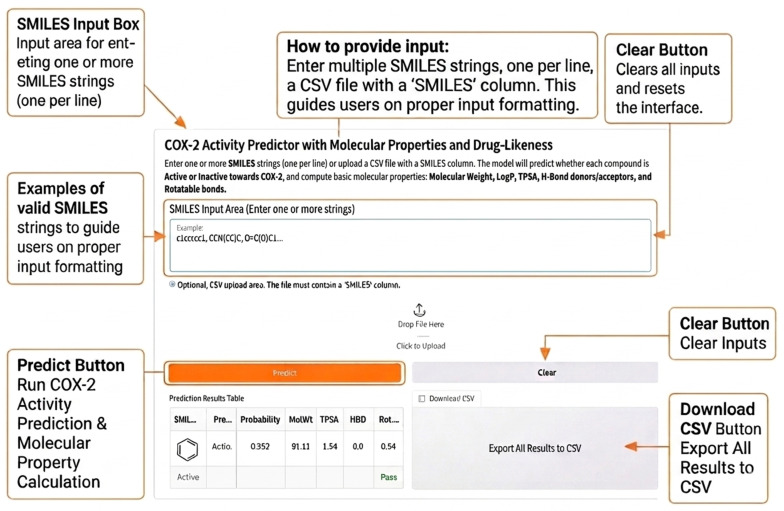
Annotated schematic of the web interface used for model deployment and compound activity prediction.

Figure 9 illustrates the graphical user interface of the deployed COX-2 activity prediction web application. The interface was designed to be simple and intuitive, allowing users to interact with the predictive model without requiring programming expertise. As shown in Figure 9, users can enter one or more molecular structures using SMILES notation through the main input box, with the example SMILES strings provided to guide proper formatting. Once the input is submitted, the prediction button triggers the full inference pipeline, including COX-2 activity classification and calculation of selected molecular and drug-likeness properties. The predicted results are displayed immediately in a structured table, enabling straightforward interpretation of activity status and associated scores. In addition, the interface includes utility features such as a clear button to reset the input fields and an option to download the prediction results as a CSV file, facilitating further analysis and integration into virtual screening workflows. Overall, the design of the interface emphasizes usability and practicality, supporting rapid exploration of new chemical structures in a web-based environment (https://huggingface.co/spaces/N80-ouass/cox-pred) (accessed on 13 April 2026).

### 2.7. Model Validation Using Known COX Inhibitors

To further assess the predictive capability of the developed model, a validation study was conducted using a set of known COX inhibitors and structurally related compounds. As shown in Table 3, the model successfully identified all selective COX-2 inhibitors, including celecoxib, etoricoxib, rofecoxib, valdecoxib, and lumiracoxib, as active compounds with high prediction probabilities (>0.94). This demonstrates the model’s strong ability to recognize key structural features associated with COX-2 inhibition.

In contrast, compounds that are not selective COX-2 inhibitors, such as sulfamethoxazole and acetazolamide, were correctly classified as inactive, indicating good specificity. Non-selective COX inhibitors, including aspirin and indomethacin, were predicted as inactive or borderline, which is consistent with their different inhibition profiles.

A notable observation is the prediction of parecoxib as inactive with moderate probability (0.43). This can be explained by its nature as a prodrug that requires metabolic activation to form valdecoxib, suggesting that the model primarily captures structural features of the active form rather than prodrug behavior.

Additionally, methazolamide was predicted as active with low confidence (probability ≈ 0.53) but was classified outside the applicability domain (AD). This highlights the importance of AD analysis, as predictions for compounds outside the model’s chemical space should be interpreted with caution.

Overall, these results confirm that the model is capable of distinguishing between selective COX-2 inhibitors and unrelated compounds, while also appropriately reflecting uncertainty in borderline or structurally distinct cases.

## 3. Discussion

The present study demonstrates that machine learning approaches, particularly ensemble-based methods, can effectively predict COX-2 inhibitory activity from 2D molecular representations. By integrating a curated ChEMBL dataset, Morgan fingerprint descriptors, systematic benchmarking, and SHAP-based interpretability analysis, a robust and chemically meaningful predictive framework was established.

Among the evaluated models, the Random Forest classifier exhibited the most consistent performance across both internal and external validation datasets, achieving a strong balance between precision and recall with good generalization capability (external ROC–AUC ≈ 0.82). The observed decrease in performance from internal to external validation is expected and reflects the increased structural diversity of the external dataset rather than model overfitting.

The robustness of the model was further supported by Y-randomization analysis, where performance dropped to near-random levels (AUC ≈ 0.5), indicating that the model captures meaningful structure–activity relationships rather than chance correlations. In addition, applicability domain analysis confirmed that most compounds fall within the defined chemical space, supporting the reliability of predictions, while compounds outside the domain exhibited lower confidence, as expected.

In addition to evaluating predictive performance and robustness, understanding the underlying factors driving model predictions is essential. SHAP-based interpretation revealed that influential fingerprint features correspond to chemically meaningful motifs associated with COX-2 inhibition, including aromatic systems, heterocyclic scaffolds, and sulfonamide-like groups [23,24]. The distribution of SHAP contributions suggests that the model integrates multiple complementary substructural features rather than relying on a single dominant pharmacophore.

To further contextualize these findings at the molecular level, a case study analysis was conducted using representative COX-2 inhibitors. The SHAP analysis identified key fingerprint features corresponding to aromatic ring systems and sulfonamide-related substructures, which are characteristic of celecoxib. Mechanistically, the sulfonamide group is known to occupy the COX-2 side pocket and interact with key residues such as Arg513 and His90, which play a well-established role in isoform selectivity [25,26]. Meanwhile, the presence of aromatic rings is consistent with interactions within the hydrophobic channel of the enzyme, involving residues such as Val349, Leu352, and Tyr385 [25]. These structural and interaction patterns are in agreement with previously reported binding modes of selective COX-2 inhibitors, including celecoxib, which exploits the unique side pocket of COX-2 to achieve selectivity [26].

Overall, this case study suggests that the model captures chemically and biologically relevant features that are consistent with the established pharmacophore of COX-2 inhibitors. Consistent with these observations, validation using known COX inhibitors further confirmed model reliability, as selective COX-2 inhibitors were correctly predicted as active with high confidence, while unrelated compounds were classified as inactive.

To place these findings in the context of the existing literature, recent studies on COX-2 inhibitor prediction were examined. Ligand-based QSAR approaches using molecular fingerprints and classical machine learning algorithms remain widely used. For example, Dibia et al. [27] reported strong classification performance (accuracy = 0.9484, MCC = 0.8741) using an XGBoost model trained on ChEMBL-derived data. While these results demonstrate the effectiveness of boosting methods, they also highlight a common limitation in QSAR studies related to reliance on random train–test splits.

This limitation has been partially addressed in subsequent studies. In a broader modeling framework, Tian et al. [28] developed large-scale ensemble models integrating multiple molecular representations and algorithms, including external validation datasets. Their results highlight a recurring observation in QSAR modeling, where performance tends to decrease on external datasets (e.g., MCC ≈ 0.603), reflecting the inherent difficulty of generalizing across chemically diverse compounds.

Alternatively, deep learning approaches have been explored to capture more complex relationships. Yasir et al. [29] applied a GraphConv model to the DUD-E benchmark and reported high predictive performance. However, benchmark datasets such as DUD-E rely on artificially generated decoys and may not fully represent real-world chemical distributions.

In parallel, increasing attention has been given to model interpretability. Studies such as Rudrapal et al. [30] and Yang et al. [31] incorporated explainability techniques including SHAP and LIME to better understand feature contributions. While these approaches improve transparency, predictive performance remains variable depending on dataset quality and modeling strategies.

Overall, the existing literature demonstrates significant progress in COX-2 prediction, particularly in terms of model diversity and performance optimization. However, recurring challenges include dataset heterogeneity, evaluation strategies, and reproducibility. Building on these observations, the present study addresses these challenges by combining a robust machine learning pipeline with transparent data processing, external validation, and explainability.

While Morgan fingerprints (ECFP4) were selected due to their strong ability to capture local structural environments, alternative representations such as MACCS keys or physicochemical descriptors could also be considered. Nevertheless, ECFP4 provides higher structural resolution compared to predefined key-based fingerprints, making it suitable for modeling diverse chemical scaffolds such as COX-2 inhibitors. Future work will explore hybrid descriptor sets to further enhance predictive performance.

Despite these strengths, certain limitations remain. The use of 2D descriptors restricts the capture of three-dimensional and binding-specific interactions, and the model does not explicitly address COX-1/COX-2 selectivity. Future studies should incorporate 3D descriptors, multi-target modeling, and more diverse datasets to improve predictive scope.

Importantly, the present study ensures full reproducibility through the public availability of the dataset, source code, and a permanent DOI, thereby enhancing transparency and practical applicability.

Overall, this study demonstrates that explainable 2D-QSAR machine learning models can provide accurate, interpretable, and practically useful predictions for COX-2 inhibitory activity, supporting their application in drug discovery workflows.

## 4. Materials and Methods

### 4.1. Bioactivity Data Collection and Curation

Bioactivity data for human Cyclooxygenase-2 (COX-2; *PTGS2*, ChEMBL target ID: CHEMBL230) were retrieved from the ChEMBL database. All associated activity records were downloaded and subjected to systematic preprocessing.

To ensure consistency across experimental measurements, only IC50 values reported in nanomolar (nM) units were retained. Records containing approximate activity qualifiers (e.g., “>” or “<“), percentage inhibition data, non-numeric entries, or missing SMILES structures were excluded. Data preprocessing was performed using Python (version 3.10) with the Pandas (version 2.2.2) and RDKit (version 2022.09.5) libraries. Machine learning models were implemented using Scikit-learn (version 1.2.2), with additional libraries including NumPy (version 1.26.4) and Joblib (version 1.3.2). The web application was developed using Gradio (version 4.44.0) and FastAPI (version 0.112.2).

To reduce the impact of potential experimental noise, IC50 values exceeding 1,000,000 nM were removed. Duplicate compounds were eliminated based on their canonical SMILES representations to ensure structural uniqueness within the dataset.

For numerical stability and improved modeling performance, IC50 values were transformed into their negative logarithmic form (pIC50) using the standard equation:

pIC50 = −log10(IC50 [M])

However, activity classification was performed directly on IC50 values to maintain interpretability and consistency with commonly used thresholds.

For binary classification, compounds were labeled as Active when IC50 ≤ 1000 nM and labeled as Inactive otherwise. The 1 µM cutoff is widely adopted in ligand-based modeling studies as a standard threshold to distinguish biologically relevant inhibition from weak activity, while providing a practical balance between sensitivity and specificity [16,21].

After preprocessing, the final dataset consisted of 2052 compounds, including 1270 active and 782 inactive molecules, indicating a moderate class imbalance (approximately 62% active and 38% inactive).

The final curated dataset is provided as Supplementary Table S1, including compound identifiers, SMILES representations, IC50 values, and assigned activity labels. This dataset served as the basis for molecular fingerprint generation and subsequent machine learning modeling.

### 4.2. Molecular Representation

Molecular structures were encoded using Morgan circular fingerprints, as implemented in RDKit, corresponding to Extended Connectivity Fingerprints (ECFP) [32]. Canonical SMILES strings were converted into fixed-length ECFP representations with a radius of 2 (ECFP4) and a vector size of 2048 bits.

These fingerprints were generated as binary bit vectors, where each bit encodes the presence or absence of specific circular substructures centered on individual atoms [33]. By iteratively capturing local atomic environments, Morgan fingerprints provide a robust and informative representation of molecular structure and have been widely used in QSAR modeling and activity prediction tasks [26].

While the use of a fixed-length bit vector may introduce hash collisions, a size of 2048 bits is commonly adopted as a practical compromise between computational efficiency and representational capacity.

The resulting fingerprint vectors were converted into NumPy arrays and used as standardized input features for all machine learning models evaluated in this study.

### 4.3. Model Development and Training Procedure

Several machine learning algorithms were evaluated to identify the most suitable model for predicting COX-2 inhibitory activity. All models were trained on the curated dataset encoded with Morgan fingerprints, as described previously. The evaluated classifiers were implemented primarily using the Scikit-learn library [34] and included Random Forest, Gradient Boosting, Support Vector Machine (RBF kernel), k-Nearest Neighbors (KNN), Naïve Bayes, Multi-Layer Perceptron (MLP) [35], Decision Tree, and a tree-based XGBoost classifier [36].

The dataset was divided into training and validation subsets using an 80:20 stratified hold-out split to preserve class distribution, with a fixed random seed (random_state = 42) to ensure reproducibility. To further improve model robustness and assess stability, hyperparameter optimization was performed using 5-fold stratified cross-validation (StratifiedKFold, n = 5) in combination with GridSearchCV.

To mitigate potential class imbalance, class weighting was applied in selected models (e.g., Random Forest and Decision Tree classifiers). All models were trained using the same molecular fingerprint representation to ensure a fair comparison.

Model-specific hyperparameters were optimized during the cross-validation process. For example, the number of trees in ensemble models (e.g., n_estimators = 400 for Random Forest and 600 for XGBoost), the architecture of the MLP (two hidden layers of sizes 256 and 128), and the neighborhood size for KNN (k = 5 with distance weighting) were determined through systematic tuning.

Model performance on the validation set was assessed using accuracy, precision, recall, and F1-score. These metrics enabled systematic comparison of candidate algorithms and guided the selection of the final model, considering not only predictive performance but also stability and interpretability.

### 4.4. External Validation of the Selected Models

To evaluate model generalizability, an independent external test set comprising 597 COX-2 bioactivity records was retrieved from the ChEMBL database [21,22] and was not used during model development.

To ensure a rigorous and unbiased evaluation, the external dataset was carefully processed to maintain independence from the model development data. Compounds with identical SMILES representations were removed to eliminate duplicates and prevent data leakage. In addition, further filtering was applied to reduce structural redundancy, ensuring that the external set remained chemically diverse and representative of the broader COX-2 chemical space.

The same preprocessing pipeline—including SMILES sanitization and Morgan fingerprint generation—was applied to the external dataset to ensure methodological consistency with the training data. After preprocessing, the final external dataset (Table S2) consisted of 597 compounds, including 254 active and 343 inactive molecules.

Based on internal validation performance, the two best-performing models (Random Forest and XGBoost) were selected for external evaluation. Their predictive performance was assessed using accuracy, precision, recall, F1-score, and ROC–AUC.

### 4.5. Y-Randomization Test

To assess the robustness of the developed binary classification model and exclude potential chance correlations, a Y-randomization (permutation) test was performed using the Random Forest classifier. In this procedure, the training activity labels were randomly shuffled while keeping the molecular fingerprint descriptors unchanged. The model was then retrained using the same configuration as in the original experiment and evaluated on the validation set.

This process was repeated over 20 independent runs. For each iteration, performance metrics including accuracy and ROC–AUC were recorded. The distributions of these metrics under randomized label conditions were compared with those obtained from the original model. In addition, empirical *p*-values were calculated to quantify the likelihood of achieving the observed performance by chance.

A significant decrease in performance under label randomization, together with low *p*-values, confirms that the predictive ability of the model is not due to random correlations but reflects meaningful structure–activity relationships.

### 4.6. Applicability Domain Analysis

To evaluate the reliability of model predictions for novel compounds, an applicability domain (AD) analysis was performed using a similarity-based approach [37]. Morgan fingerprint representations were used to quantify structural similarity between compounds.

A k-nearest neighbors (kNN) method (k = 5) was applied using the Jaccard distance metric, which is well-suited for binary fingerprint data and is equivalent to the Tanimoto coefficient commonly used in cheminformatics [38]. For each compound, the mean similarity to its five nearest neighbors in the training set was calculated.

The distribution of mean similarity values within the training set was used to define the applicability threshold, which was set as the 5th percentile of the training similarity distribution. Compounds with similarity values below this threshold were considered outside the applicability domain (OOD).

Validation and external compounds falling outside this domain were flagged, and their predictions were interpreted with caution, as they lie outside the chemical space adequately represented by the training data.

This similarity-based AD framework ensures that model predictions are considered reliable only for compounds structurally similar to those in the training dataset.

### 4.7. Model Interpretation Using SHAP Analysis

To gain insight into the molecular features underlying the predictions of the optimized binary Random Forest model, SHAP (SHapley Additive exPlanations) analysis was performed. SHAP is a game-theoretic interpretability framework that quantifies the contribution of each input feature to the model output. SHAP values were computed using the TreeExplainer algorithm implemented in the SHAP library, which is specifically designed for tree-based models [39].

The analysis was conducted on a representative subset of the training data to ensure computational efficiency while preserving the distribution of the chemical space. SHAP values were calculated for each compound to quantify the contribution of individual Morgan fingerprint bits to the predicted probability of the active class.

### 4.8. Web-Based Interface Development

A web-based interface was developed to enable programmatic access to the trained prediction pipeline. The application was implemented using the Gradio framework and deployed on the HuggingFace Spaces platform (https://huggingface.co/spaces, accessed on 13 April 2026), using a Docker-based environment to ensure reproducibility and consistent runtime configuration [40].

The interface accepts molecular structures in SMILES format as input and returns predicted COX-2 inhibitory activity along with the corresponding probability score. Molecular features are generated dynamically using Morgan fingerprints in the same manner as during model training, ensuring consistency between training and inference.

The application is publicly accessible and does not require local installation, providing a user-friendly platform for real-time prediction and virtual screening.

## 5. Conclusions

In this study, an explainable 2D-QSAR machine learning framework was developed for the prediction of COX-2 inhibitory activity using molecular fingerprints derived from SMILES representations. Among the evaluated models, the Random Forest classifier demonstrated the most reliable and consistent performance across internal and external validation, confirming its robustness and generalization capability.

The integration of Y-randomization, applicability domain analysis, and external validation ensured that the predictive performance was not driven by chance and that the model operated within a well-defined chemical space. Furthermore, SHAP-based interpretation provided valuable insight into the molecular determinants of activity, linking model predictions to chemically meaningful substructures associated with COX-2 inhibition.

Importantly, the developed model was implemented as an interactive web-based application, enabling real-time prediction of COX-2 inhibitory activity directly from SMILES input. This accessible interface enhances the practical utility of the model and facilitates its application in virtual screening and early-stage drug discovery.

Overall, this work demonstrates that explainable machine learning combined with 2D-QSAR modeling offers a reliable, interpretable, and practically deployable approach for COX-2 activity prediction. Future work will focus on incorporating three-dimensional descriptors and multi-target modeling strategies to improve selectivity prediction and broaden applicability.

## Figures and Tables

**Table 1:** Performance comparison of machine learning models on the validation dataset.

Model	Accuracy	Precision (Active)	Recall (Active)	F1-Score (Weighted)
Random Forest	0.808	0.833	0.856	0.807
Gradient Boosting	0.771	0.762	0.908	0.761
MLP Neural Network	0.803	0.814	0.876	0.800
Decision Tree	0.781	0.808	0.840	0.780
k-Nearest Neighbors	0.793	0.782	0.916	0.785
Naïve Bayes	0.803	0.851	0.820	0.804

**Table 2:** Y-randomization (Permutation test) results for the final Random Forest model (*n* = 20).

Metric	Original Model	Permuted Mean	Permuted Max	*p*-Value
Accuracy	0.867	0.536	0.606	0.048
ROC–AUC	0.931	0.509	0.610	0.048

**Table 3:** Validation of the developed model using known COX inhibitors and related compounds.

Compound	Prediction	Probability (Active)	In_AD	Interpretation
Celecoxib	Active	0.968	Yes	Correct (COX-2 inhibitor)
Etoricoxib	Active	0.965	Yes	Correct (COX-2 inhibitor)
Rofecoxib	Active	0.954	Yes	Correct (COX-2 inhibitor)
Valdecoxib	Active	0.952	Yes	Correct (COX-2 inhibitor)
Lumiracoxib	Active	0.947	Yes	Correct (COX-2 inhibitor)
Aspirin	Inactive	0.475	Yes	Expected (non-selective COX inhibitor)
Indomethacin	Inactive	0.320	Yes	Expected (COX-1/COX-2 non-selective)
Sulfamethoxazole	Inactive	0.210	Yes	Correct (not a COX inhibitor)
Acetazolamide	Inactive	0.180	Yes	Correct (different target)
Parecoxib	Inactive	0.430	Yes	Borderline (prodrug of valdecoxib)
Methazolamide	Active	0.531	No	Low confidence (outside AD)

## Data Availability

The original data presented in the study are openly available in [Hugging Face] at https://huggingface.co/spaces/N80-ouass/cox-pred (accessed 13 April 2026); [Github] at https://github.com/profnabila/cox2-mL-model (accessed 20 April 2026) or [Zenodo] at https://doi.org/10.5281/zenodo.19559334 (accessed 20 April 2026).

## Supplementary Materials

The following supporting information can be downloaded at https://www.mdpi.com/article/10.3390/ph19050698/s1. Table S1: Training Compounds; Table S2: External Compounds; Table S3: External Validation Dataset.

## Abbreviations

COX-2	Cyclooxygenase-2
COX-1	Cyclooxygenase-1
QSAR	Quantitative Structure–Activity Relationship
2D-QSAR	Two-Dimensional Quantitative Structure–Activity Relationship
ML	Machine Learning
ECFP	Extended Connectivity Fingerprint
SMILES	Simplified Molecular Input Line Entry System
IC50	Half-Maximal Inhibitory Concentration
pIC50	Negative Logarithm of IC50
AD	Applicability Domain
kNN	k-Nearest Neighbors
ROC	Receiver Operating Characteristic
AUC	Area Under the Curve
RF	Random Forest
SVM	Support Vector Machine
MLP	Multi-Layer Perceptron
XGBoost	Extreme Gradient Boosting
SHAP	SHapley Additive exPlanations
NSAIDs	Nonsteroidal Anti-Inflammatory Drugs
OOD	Out-of-Domain
