# Supplementary material for: An Explainable 2D-QSAR Machine Learning Approach for Predicting COX-2 Inhibitory Activity Using Molecular Fingerprints
Source: Pharmaceuticals (Basel). 2026 Apr 29;19(5):698. doi: 10.3390/ph19050698 (PMC13209868; doi:10.3390/ph19050698)
Supplement: Supplementary file 1 [file pharmaceuticals-19-00698-s001.zip › Table S1 Training Compounds.pdf]

SMILES	IC50	Binary_Clas	pIC50					
CC(/C=C/c1	760	1	6.119186					
COc1cc2cc	760	1	6.119186					
C=CCNc1[n	30000	0	4.522879					
Cc1ccccc1N	57000	0	4.244125					
C/C(CCC(=C	17	1	7.769551					
O=CNCc1cc	120	1	6.920819					
Cc1ccc(C2=	580	1	6.236572					
CCCC/C(=C	500	1	6.30103					
CN1C(=O)C	60	1	7.221849					
CC(C)C[C@	10000	0	5					
CC1Cc2c(O	3	1	8.522879					
NC(=S)N(O	180	1	6.744727					
CCCC/C(=C	250	1	6.60206					
CC(C)(C)c1c	10000	0	5					
CCCCCCCC	250	1	6.60206					
COc1ccc(-c	1400	0	5.853872					
Cc1ccc(-c2c	120	1	6.920819					
COC1(c2cc	40000	0	4.39794					
NS(=O)(=O	25800	0	4.58838					
Cc1cc(-c2cc	27	1	7.568636					
COc1cc(OC	10000	0	5					
Cc1cc(OCc2	830	1	6.080922					
O=C(Nc1cc	90	1	7.045757					
CCCCCCCC	200	1	6.69897					
Cc1ccc(C(C	430	1	6.366532					
COc1cc(/C=	500	1	6.30103					
CC1Cc2c(O	125	1	6.90309					
COc1ccc(/C	1100	0	5.958607					
CCCCC1Oc2	59	1	7.229148					
CCC(O)(c1c	600	1	6.221849					
C/C(=N\OC	20	1	7.69897					
CCOC(=O)C	68	1	7.167491					
COc1cc(/C=	4000	0	5.39794					
CC1(C)CCC[	150000	0	3.823909					
c1cnc2nc(-	3550	0	5.449772					
O=C1OCc2c	720	1	6.142668					
O=C(c1cccs	380	1	6.420216					
Cn1cc(NC(=	290	1	6.537602					
Oc1ccccc1-	3000	0	5.522879					
O=C(Cc1cn	1600	0	5.79588					
COc1cc2oc	1400	0	5.853872					
CCCCCCCC	1050	0	5.978811					
CC(=O)Oc1	3100	0	5.508638					
COc1ccc(/C	120	1	6.920819					
CCCCCCC(S	8	1	8.09691					
CN(O)C(=O	1000	0	6					
N#CNC(=N	420	1	6.376751					
COc1ccc2cc	12500	0	4.90309					
Cc1ccccc1N	50000	0	4.30103					
CCCCCC[C@	300	1	6.522879					

COC1(c2cc	92900	0	4.031984
COC1(c2cc	830	1	6.080922
Oc1nc(-c2c	860	1	6.065502
CC(C1CC1c	900	1	6.045757
Cc1nccn1-c	340	1	6.468521
COc1cc(/C=	13900	0	4.856985
CCOC(=O)c	160	1	6.79588
C/C(=N\OC	14	1	7.853872
COc1cc(/C=	2	1	8.69897
CCCCCCCCl	500	1	6.30103
O=C(O)/C=	60	1	7.221849
Nc1cc(C(=C	900	1	6.045757
COc1cccc(-	430	1	6.366532
CCCCCCC(S	800	1	6.09691
CCCCCCc1c	58	1	7.236572
COc1cc2oc	74300	0	4.129011
CC1Cc2c(O	80	1	7.09691
Sc1cccc2cc	200000	0	3.69897
CC(C)c1ccc	160	1	6.79588
O=C(NCCC(	1500	0	5.823909
CC(C)=CCC/	10	1	8
NC(=O)N(C	57	1	7.244125
O=C1CCN(c	5	1	8.30103
NC(=O)N(C	380	1	6.420216
c1cnn2c(N(	1760	0	5.754487
N#Cc1cc(-c	20	1	7.69897
O=C1c2ccc	5300	0	5.275724
COC1(c2cc	80	1	7.09691
COc1cc([C@	380	1	6.420216
CC(C#Cc1c	700	1	6.154902
CCCC(O)C#	760000	0	3.119186
COc1ccc(/C	900	1	6.045757
CCCC/C(=C	700	1	6.154902
COc1ccc(C(	12	1	7.920819
O=C(/C=C/	36.7	1	7.435334
Cc1ccc2nc(	1530	0	5.815309
O=C(Nc1cc	33	1	7.481486
C[S+]([O-])	650	1	6.187087
CCCCCCCC(	280	1	6.552842
O=C(Cc1cn	4700	0	5.327902
CO/N=C/C1	150	1	6.823909
CC1CN(c2c	1800	0	5.744727
CCCCCNC(=	1500	0	5.823909
CCCCCCCC(	950	1	6.022276
O=C(O)CCC	4000	0	5.39794
O=c1c2ccc	3	1	8.522879
CCc1ccccc1	39000	0	4.408935
NS(=O)(=O)	50	1	7.30103
COc1ccc(C(	100000	0	4
O=C(c1cccr	120	1	6.920819
COc1cccc(-	80	1	7.09691

CC[C@](OC	740	1	6.130768
COC1(c2cc	570	1	6.244125
Cc1cc(NC(=	2090	0	5.679854
CC[C@@](	520	1	6.283997
CC[C@@]1	60	1	7.221849
N#Cc1cc(-c	2.3	1	8.638272
COC1=CC(=	310	1	6.508638
CN(O)C(=O	45	1	7.346787
CCOC(=O)c	255	1	6.59346
Cc1cc(-c2c	40000	0	4.39794
O=C(O)c1c	4600	0	5.337242
CCC(O)(c1c	1100	0	5.958607
CN1C(=O)C	100	1	7
O=c1c2ccc	5	1	8.30103
NC(=O)N(C	170	1	6.769551
C/C(=N\OC	28	1	7.552842
COC(=O)CC	190	1	6.721246
O=C1/C(=C	20	1	7.69897
COc1cc(/C=	340	1	6.468521
O=C1CCCN	99000	0	4.004365
Cc1cc(-c2c	24	1	7.619789
N#Cc1ccc(-	380	1	6.420216
CCCCC(Sc1	900	1	6.045757
C/C=C/c1c	50000	0	4.30103
CCC(O)(c1c	19	1	7.721246
CC(c1cc2cc	0.26	1	9.585027
COc1ccc(O	130	1	6.886057
CC1Cc2c(O	420	1	6.376751
CC1=C(C)C(	1700	0	5.769551
COC(=O)c1	7200	0	5.142668
NC(=O)N(C	510	1	6.29243
O=[N+]([O-	3400	0	5.468521
O=C(O)C(S	800	1	6.09691
CCC(Sc1nc(	2800	0	5.552842
COC1(c2cc	40000	0	4.39794
O=C(O)/C=	47	1	7.327902
O=C(Cc1cc	600	1	6.221849
O=C(O)C(S	700	1	6.154902
Cc1c(C(=O)	1640	0	5.785156
COc1cc(C2	370	1	6.431798
CCC(O)(c1c	800	1	6.09691
C/C(=C\CC/	77	1	7.113509
N#CC1=C(N	1660	0	5.779892
CC(CC(=O)(	70	1	7.154902
CC1CN(c2c	8400	0	5.075721
CC(C)(C)OC	81000	0	4.091515
O=C(O)C(S	240	1	6.619789
CCCCc1c(-c	100	1	7
O=c1cc(-c2	1700	0	5.769551
O=C(O)c1c	500	1	6.30103
CC[C@@H	40	1	7.39794

NC(=O)N(C	170	1	6.769551
CCCC(=O)N	52000	0	4.283997
CC(C)Cc1cc	2500	0	5.60206
CC1=CCCC2	30.51	1	7.515558
CC(C#Cc1cc	100	1	7
CCc1ccc(CS	100000	0	4
COc1cc(/C=	5480	0	5.261219
Cc1c(C)c2c	71	1	7.148742
COC1(c2cc	14	1	7.853872
O=[N+]([O-	50000	0	4.30103
O=S(=O)(N	10000	0	5
O=C1Nc2cc	5700	0	5.244125
C[C@H](C#	160	1	6.79588
CCOC(=O)c	10000	0	5
CCCCC/C=C	50000	0	4.30103
O=C1CCN(	1100	0	5.958607
CC(=O)Oc1	200	1	6.69897
Cn1ncc(NC	41	1	7.387216
O=C(/N=c1	380	1	6.420216
COC1(c2cc	60	1	7.221849
CCOC(=O)c	120	1	6.920819
Cc1ccc(O)c	100000	0	4
O=c1c2ccc	30	1	7.522879
Cc1cc(NC(=	23	1	7.638272
CCCCC(Sc1	600	1	6.221849
CC(=O)N(C	150	1	6.823909
O=C1CCCN	53000	0	4.275724
COC(=O)c1	45000	0	4.346787
CC(C)=CCC	350	1	6.455932
CCCCC(Sc1	1200	0	5.920819
CNC(=O)N(	45	1	7.346787
O=C1OCc2	380	1	6.420216
COc1ccc(/C	150	1	6.823909
O=C(NCCC	77600	0	4.110138
COC1=CC(=	1900	0	5.721246
COc1cccc(/	100000	0	4
NC(=O)N(C	60	1	7.221849
C[C@H](C#	600	1	6.221849
CCOC(=O)c	86	1	7.065502
CSc1ccc(-c	680	1	6.167491
CCCCCCCC	40	1	7.39794
CC(c1ccc(-c	4000	0	5.39794
COc1cc(/C=	1100	0	5.958607
CC(C)(C)c1c	6250	0	5.20412
CCCCCCOc1	480	1	6.318759
C#CCn1ccc	0.9	1	9.045757
OC1(c2cccc	50	1	7.30103
Cc1cccc(N2	2000	0	5.69897
NC(=O)N(C	1000	0	6
CC1(C)Cc2c	1700	0	5.769551
COc1ccc2c	3000	0	5.522879

O=C(O)c1n	6000	0	5.221849
Clc1ccc(-c2	300	1	6.522879
CN(C)S(=O)	65	1	7.187087
CC1CN(c2c	2700	0	5.568636
CCCC/C(=C	2100	0	5.677781
CC1=NN(c2	570	1	6.244125
O=C(OCC#(	15000	0	4.823909
C[C@@H](	50	1	7.30103
CCC1Oc2c(	63	1	7.200659
COc1cc([C(	10	1	8
CSc1ccccc1	100	1	7
CCOC(=O)c	130	1	6.886057
COC1=CC(=	330	1	6.481486
CCCCCC1=(	1370	0	5.863279
Cc1ccc(S(=(	2.07	1	8.68403
COc1ccc(C(	574	1	6.241088
CSc1ccc(CC	1	1	9
CCCCCCCC(	770	1	6.113509
Cc1ccc(Cn2	10	1	8
COc1ccc(-c	10	1	8
Cn1cc(NC(=	30	1	7.522879
CCOC(=O)c	420	1	6.376751
CC(C)=CCC/	200	1	6.69897
COc1cc(-c2	480	1	6.318759
CNc1sc(-c2	50000	0	4.30103
NC(=O)N(C	900	1	6.045757
CC[C@](OC	110	1	6.958607
CC1Cc2c(O	23	1	7.638272
CCOC(=O)c	580	1	6.236572
CC1(C)Cn2(	2300	0	5.638272
COc1ccc(/C	600	1	6.221849
C[C@@H]1	50000	0	4.30103
CC(C)NC(=(	3410	0	5.467246
CCCCCCOc1	280	1	6.552842
O=C1CCN((	3700	0	5.431798
CCCC1Oc2(	870	1	6.060481
CC(C)(C)c1(	300	1	6.522879
O=C(OCc1c	170	1	6.769551
CN(C)c1ccc	80	1	7.09691
Oc1ccc(Nc2	100	1	7
COc1cc(O)(	1140	0	5.943095
Cc1ccc(S(=(	2.6	1	8.585027
O=C(O)/C=	1200	0	5.920819
CC1CN(c2c	5800	0	5.236572
Cc1ccc2c(c	10000	0	5
COc1ccc(S2	350	1	6.455932
CCCCCCCC(	340	1	6.468521
COc1cc(-c2	116	1	6.935542
COc1ccc(/C	300	1	6.522879
O=C(O)c1c(	31	1	7.508638
Cc1ccc(C2=	860	1	6.065502

Cc1ccc(N2(	277000	0	3.55752
NC(=O)N(C	213	1	6.67162
CC(=O)N(C)	40	1	7.39794
CCOC(=O)c	650	1	6.187087
COc1ccc(/C	960	1	6.017729
O=C(O)c1c	1200	0	5.920819
O=C(OCC#(	30000	0	4.522879
CC(C)(C)c1	30000	0	4.522879
COc1cc2c(	4500	0	5.346787
Cn1ncc(NC	34	1	7.468521
NS(=O)(=O)	3450	0	5.462181
CCCc1cc(Cl	20	1	7.69897
COc1cc(-c2	1300	0	5.886057
CCCCCCCC(	560	1	6.251812
O=[N+]([O-	150	1	6.823909
Cc1cc(OCc2	570	1	6.244125
Cc1ccc(C(C	3160	0	5.500313
O=c1c2ccc(	100	1	7
NS(=O)(=O)	16150	0	4.791827
C/C(=N\OC	60	1	7.221849
CCC(O)(c1c	1840	0	5.735182
COc1cc(/C=	13900	0	4.856985
CCCCCCCC(	175	1	6.756962
CN(C(=O)c1	12000	0	4.920819
CS(=O)(=O)	10000	0	5
Cc1cc(NC(=	2170	0	5.66354
O=C(COc1c	1200	0	5.920819
COC1(c2cc(	3400	0	5.468521
O=C(O)/C((	181	1	6.742321
C[S+]([O-])(	710	1	6.148742
Cn1cc(NC(=	83	1	7.080922
C/C(=N\O[(	34	1	7.468521
c1ccc2cc(C	40000	0	4.39794
CCOC(=O)c	110	1	6.958607
CNc1oc(-c2	50000	0	4.30103
Cc1ccc2nc(	253	1	6.596879
CCCCCC(=C	1600	0	5.79588
CCC(Cc1ccc	3000	0	5.522879
NC(=O)N(C	970	1	6.013228
O=C1NCCN	8800	0	5.055517
CCOC(=O)c	96	1	7.017729
Cc1c[nH]c(	7600	0	5.119186
O=C(O)/C(=	31	1	7.508638
NC(=O)N(C	53	1	7.275724
CCCc1ccc2(	31	1	7.508638
Cc1c(C)c2c	290	1	6.537602
CCOCc1cc((	291	1	6.536107
CCCCCCCC(	6000	0	5.221849
Cc1nc(O)c(	5100	0	5.29243
COC1(c2cc(	2600	0	5.585027
CCCC/C=C\	3000	0	5.522879

C/C(=C\CC/	265	1	6.576754
CC(=NOC(c	32	1	7.49485
Cc1cccc(NC	10	1	8
CCCCC/C=C	7400	0	5.130768
COc1c(NC(=	400	1	6.39794
CCN(CC)C(=	3220	0	5.492144
CC(C)C1CN	20000	0	4.69897
Cc1cccc(OC	6	1	8.221849
CC(c1ccc(C	5000	0	5.30103
Cc1cc(-c2cc	57	1	7.244125
CC1(C)Cc2c	2400	0	5.619789
C/C(=N\O)c	200	1	6.69897
NC(=O)N(O	68	1	7.167491
CCC(OC)(c1	900	1	6.045757
CCCCCCCC(	115	1	6.939302
NC(=O)N(O	310	1	6.508638
CN(C)c1ccc	2200	0	5.657577
CCCCCCCC(	1500	0	5.823909
O=C(c1[nH	5700	0	5.244125
CCCCCCC1(	170	1	6.769551
Cc1cc(C(=C	1000	0	6
COc1ccc(C(	80000	0	4.09691
CO/N=C(\C	270	1	6.568636
CC(c1cc2cc	1600	0	5.79588
Cc1ccc(C(C	940	1	6.026872
COc1ccc(CI	6	1	8.221849
O=C1c2ccc	320	1	6.49485
NC(=O)N(O	140	1	6.853872
COc1ccc2o	82400	0	4.084073
CCc1cc(-c2	760	1	6.119186
CCCCCc1cc	940	1	6.026872
COC(C)(c1r	500	1	6.30103
C=CCc1cc(/	65	1	7.187087
CCCCC(Sc1	500	1	6.30103
COCC1(c2c	170	1	6.769551
CC(C)N(O)C	130	1	6.886057
COC1=CC(=	200	1	6.69897
CC1Cc2c(O	35	1	7.455932
COc1ccc(N	600	1	6.221849
N#Cc1ccc(-	600	1	6.221849
CC1=NN(C(	100	1	7
CCOC(=O)c	2490	0	5.603801
Cc1c(O)c(C	230	1	6.638272
CCCCCC(=C	460	1	6.337242
CNC(=O)N(	100	1	7
Cc1cc(/C=C	170	1	6.769551
CCC(O)(c1c	1000	0	6
CCCCC(Sc1	1400	0	5.853872
O=C1CCCC(	18440	0	4.734239
CC(C)(C)c1	10000	0	5
O=C(O)C(S	700	1	6.154902

CC[C@@](	10000	0	5
CC(C1CC1c	1100	0	5.958607
CCCCc1c(-c	500	1	6.30103
CCOC(=O)c	330	1	6.481486
CCN(Cc1cc	623	1	6.205512
CC[C@@](	4500	0	5.346787
CC1(C)Cc2c	88	1	7.055517
CCOC(=O)c	200	1	6.69897
COc1ccc(/C	80	1	7.09691
C/C(=C\CC/	10	1	8
CCC1(c2ccc	150	1	6.823909
O=C(O)/C=	28	1	7.552842
CSc1ccc(CC	41000	0	4.387216
CC1Cc2c(O	50	1	7.30103
CCCCCCC(S	600	1	6.221849
CCN([C@@	5500	0	5.259637
CCOC(=O)c	4760	0	5.322393
CCC(=O)NC	100000	0	4
CCCCCCC(S	300	1	6.522879
COC1(c2cc	400	1	6.39794
CC1=CCCC2	14.38	1	7.842241
CCCCc1cc(	2200	0	5.657577
CSc1ccc(-c2	5000	0	5.30103
CCCCCc1cc	3000	0	5.522879
CCCCC(Sc1	1200	0	5.920819
OCc1cc(-c2	260	1	6.585027
COc1cc(CC	1100	0	5.958607
O=C(OCC#C	15000	0	4.823909
C/C(=N\OC	20	1	7.69897
C[C@H](C#	80	1	7.09691
C/C(NCc1cc	70000	0	4.154902
O=c1c2ccc	61.6	1	7.210419
C=C1CCC[C	200000	0	3.69897
CSc1ccc(-c2	4320	0	5.364516
O=c1cc(-c2	0.8	1	9.09691
Cc1ccc(-c2	900	1	6.045757
CCOC(=O)c	49	1	7.309804
COc1cc(/C=	240	1	6.619789
CCCC/C(=C	550	1	6.259637
C[S+]([O-])	710	1	6.148742
O=C1NCCN	3000	0	5.522879
CC(c1ccsc1	2400	0	5.619789
COC(C)c1cc	1200	0	5.920819
CC1Cc2c(O	175	1	6.756962
Oc1cc2cc(c	1.85	1	8.732828
CCOC(=O)c	130	1	6.886057
CCCCCCCC(	230	1	6.638272
CC[C@](O)	21	1	7.677781
O=[N+]([O-	8600	0	5.065502
CCCCCCCC(	210	1	6.677781
CC(C)(C)c1c	4000	0	5.39794

COC1(c2cc	20	1	7.69897
COc1ccc2c	1600	0	5.79588
CC1OC(=O)	1300	0	5.886057
CCCCC/C=C	2000	0	5.69897
O=C1C2CC	310	1	6.508638
Oc1ccc(-c2	50	1	7.30103
CCCC(C#Cc	300	1	6.522879
CCCCCCCC	170	1	6.769551
O=C(/C=C/	10000	0	5
Cc1cc(NC(=	2070	0	5.68403
O=C(O)Cc1	3400	0	5.468521
c1coc(-c2n	7000	0	5.154902
CCC(Sc1nc(	3600	0	5.443697
N#Cc1cc(-c	120	1	6.920819
CCCCCCc1c	220	1	6.657577
CC(C)(C)c1c	60	1	7.221849
CC(C)N(O)C	70	1	7.154902
CC(=O)N(C)	70	1	7.154902
CC1CCC(=C	56200	0	4.250264
CCOC(=O)c	100	1	7
COC(=O)c1	160	1	6.79588
COCOc1ccc	2930	0	5.533132
CC1Cc2c(O	170	1	6.769551
Cc1cc(OCC	426	1	6.37059
COC(c1ccc	7000	0	5.154902
O=C1NCCN	50000	0	4.30103
CC(c1ccc(-c	1400	0	5.853872
COc1cc(C(=	460	1	6.337242
COC(=O)CC	1.06	1	8.974694
CCCCCCOc1	128	1	6.89279
O=C(Nc1cc	170	1	6.769551
COc1ccc(C	250	1	6.60206
NC[C@H](C	10000	0	5
CCCCCCC(S	8	1	8.09691
CC(C)(C)Sc1	8	1	8.09691
O=C(O)Cc1	3900	0	5.408935
COc1cc(-c2	439	1	6.357535
CCC(OC)(c1	40000	0	4.39794
CCCCCCCC	86	1	7.065502
N#Cc1cc(-c	70	1	7.154902
NS(=O)(=O)	91100	0	4.040482
COc1cccc(/	22000	0	4.657577
COC1(c2cc	200	1	6.69897
O=c1c2ccc	100	1	7
CCOC(=O)c	150	1	6.823909
Nc1ccc2[nH	66000	0	4.180456
CSc1ccc(CC	1	1	9
CC(C)(C)c1c	300	1	6.522879
COc1ccc(C(	6150	0	5.211125
Cc1ccc(C2=	1460	0	5.835647
CC(CO/N=C	110	1	6.958607

CS(=O)(=O)	270	1	6.568636
CCCCCCOc1	280	1	6.552842
O=[N+]([O-	8000	0	5.09691
CC(C#Cc1c	110	1	6.958607
Cc1ccc(-c2	520	1	6.283997
CCCCCCCC	190	1	6.721246
CCCCC(Sc1	1400	0	5.853872
CC[C@](O)	31	1	7.508638
CC(Oc1ccc(	190	1	6.721246
CC1Cc2c(O	60	1	7.221849
COC1(c2nc	40000	0	4.39794
[N-]=[N+]=	640	1	6.19382
COC1(c2cc	700	1	6.154902
CCCCCCCC	290	1	6.537602
COC1(c2cc	600	1	6.221849
Cc1cc(-c2c	36	1	7.443697
CC(c1ccc(-	3400	0	5.468521
Cc1ccc(S(=	3.6	1	8.443697
COc1cc(/C=	310	1	6.508638
Cc1c[nH]c(	7000	0	5.154902
O=C(O)c1c	20	1	7.69897
C[C@H](C#	1000	0	6
COc1ccccc1	10000	0	5
COC1(c2cc	24600	0	4.609065
NS(=O)(=O)	5290	0	5.276544
COc1cccc(/	600	1	6.221849
C/C(=N\OC	1510	0	5.821023
O=C1CCCN	5200	0	5.283997
Cn1ncc(NC	28	1	7.552842
NC(=O)N(C	42	1	7.376751
O=C(O)/C=	21	1	7.677781
CC(C)(C)c1	50000	0	4.30103
CC(c1cccc(	140	1	6.853872
Oc1ccc(OC	5000	0	5.30103
Cc1cc(C(C)	480	1	6.318759
COc1cc(CC	400	1	6.39794
C[S+]([O-])	700	1	6.154902
Cc1ccc(C)n	600	1	6.221849
O=C(NC1c2	67000	0	4.173925
Clc1ccc(-c2	50	1	7.30103
COc1cccc(N	3500	0	5.455932
CN(O)C(=O	61	1	7.21467
CC1Cc2c(O	250	1	6.60206
O=C(O)CCc	1800	0	5.744727
COC1(c2cc	30	1	7.522879
CN(Cc1cccc	2300	0	5.638272
CN(O)C(=O	1000	0	6
O=C(CCCCc	150	1	6.823909
FC(F)(F)Oc1	500	1	6.30103
CCCCCC(O)	100	1	7
CCc1ccc(-c2	530	1	6.275724

OC1(c2ccc	6000	0	5.221849
C=CCn1ccc	1.4	1	8.853872
CCCCCCC(S	1500	0	5.823909
CC1Cc2c(O	22	1	7.657577
C[C@H](C#	110	1	6.958607
CN(O)C(=O	1070	0	5.970616
O=c1cc(-c2	1.9	1	8.721246
Cn1cc(NC(=	41	1	7.387216
COC1(c2cc	20	1	7.69897
Cc1ccc(C2=	6040	0	5.218963
COc1ccc2c	45400	0	4.342944
COC1(c2cc	300	1	6.522879
O=C1OCc2	400	1	6.39794
C/C(=C\CC/	210	1	6.677781
Cc1ccc2ccc	4600	0	5.337242
O=C(O)/C=	160	1	6.79588
O=C(Nc1cn	12	1	7.920819
COC1(c2cc	40	1	7.39794
CCOC(=O)c	4710	0	5.326979
Cc1cccc(N2	4800	0	5.318759
O=C1CCCC	78300	0	4.106238
O=[N+]([O-	14900	0	4.826814
CCn1cc(NC	200	1	6.69897
Cc1cc(C(=C	42800	0	4.368556
Cc1ncccc1C	10	1	8
O=C(O)c1cc	50000	0	4.30103
CC(C#Cc1cc	200	1	6.69897
CS(=O)(=O)	13500	0	4.869666
NC(=O)N(C	180	1	6.744727
CCCCCc1cc	10000	0	5
CC(C#Cc1cc	1000	0	6
COc1cc(-c2	0.83	1	9.080922
CNC(=O)N(	420	1	6.376751
CC1Cc2c(O	8	1	8.09691
Cc1ccc(S(=	3.7	1	8.431798
O=[N+]([O-	1220	0	5.91364
O=C(O)CSc	10000	0	5
CCCC/C(=C	300	1	6.522879
CCCCCCCC	155	1	6.809668
CC1Cc2c(O	60	1	7.221849
CC1Cc2c(O	55	1	7.259637
Cc1nccn1C	140	1	6.853872
CCCC/C(=C	200	1	6.69897
Cn1c(=O)cc	6930	0	5.159267
Cc1cc(-c2cc	6.3	1	8.200659
C[C@@H](	700	1	6.154902
CCN(CC)C(=	2400	0	5.619789
COc1cc(-c2	459	1	6.338187
O=C(Oc1cc	1600	0	5.79588
CCCCC(Sc1	1800	0	5.744727
CCOC(=O)c	1700	0	5.769551

FC(F)(F)c1c	100000	0	4
O=C(OCC#(	15000	0	4.823909
COc1ccc(/C	100000	0	4
CCOC(=O)c	7000	0	5.154902
S=c1[nH]nc	1260	0	5.899629
C/C=C/c1cc	50000	0	4.30103
CCCCCC(O)	100000	0	4
Cc1cccn2c(	1200	0	5.920819
O=C(O)/C(=	23	1	7.638272
Cc1cc(/C=C	120	1	6.920819
COc1cc(/C=	410	1	6.387216
CCC(O)(c1c	40000	0	4.39794
Cc1cccc(N2	7900	0	5.102373
O=C(c1cccs	380	1	6.420216
CCCCCc1cc	3000	0	5.522879
CC1=C(CC/(	450	1	6.346787
CCc1ccc(S2	180	1	6.744727
COc1ccc2cc	36200	0	4.441291
CCCc1cc(Cl	26	1	7.585027
CC1Cc2c(O	46	1	7.337242
O=C(c1cccs	380	1	6.420216
CN(O)C(=O	3200	0	5.49485
c1cc2c(cc1(	420	1	6.376751
CCCCCCC(S	600	1	6.221849
CCCCc1c(-c	265	1	6.576754
Oc1nc(-c2c	470	1	6.327902
O=C(NC1C(	100000	0	4
O=C(Nc1cc	10000	0	5
Cc1ccc2nc(	50	1	7.30103
Cn1ncc(NC	45	1	7.346787
O=C(O)c1cc	900	1	6.045757
CCCCCCC(S	10000	0	5
O=c1cc(-c2	4.3	1	8.366532
COc1cc(O)c	400	1	6.39794
COc1ccccc1	1140	0	5.943095
CCCCC/C=C	4400	0	5.356547
Cc1ccc(C)c(	240	1	6.619789
O=C(/C=C/(	3900	0	5.408935
COC1(c2ccc	1000	0	6
COC(=O)CN	2.32	1	8.634512
CCCCCCC(S	200	1	6.69897
CCCCCCC(S	8	1	8.09691
C/C(=N\OC	820	1	6.086186
Cc1cnc(C(C	40000	0	4.39794
NC(=O)N(C	640	1	6.19382
Cc1ccc(S(=(	2500	0	5.60206
CC(C)(C)CN	450	1	6.346787
Cc1cccc(N2	1300	0	5.886057
CCCCCCCC(	1490	0	5.826814
CCC(O)(c1c	2.7	1	8.568636
CC1Cc2c(O	62	1	7.207608

CCCC/C(=C	300	1	6.522879
Cc1cc2cc3c	250	1	6.60206
CCOC(=O)c	10000	0	5
Cc1cccc(N2	3400	0	5.468521
CC(C)CC(=C	1800	0	5.744727
CCCCCCCC(	600	1	6.221849
CC(c1ccco1	1950	0	5.709965
COc1ccc(/C	760	1	6.119186
CNC(=O)N(	36	1	7.443697
CN(O)C(=O	50	1	7.30103
CCCCC(Sc1	4000	0	5.39794
COc1ccc(/C	30000	0	4.522879
CCCCC(Sc1	500	1	6.30103
COc1cccc(N	6700	0	5.173925
O=C(OCC#(	20000	0	4.69897
CCCCC/C=C	44	1	7.356547
NC(=O)N(O	89	1	7.05061
CC(=O)c1cc	4900	0	5.309804
O=C(NCCC(	2200	0	5.657577
CC(C)OC(=(	102	1	6.9914
COc1cc(/C=	960	1	6.017729
COC1=CC(=	10000	0	5
CCCCCCC(S	700	1	6.154902
NC(=O)N(O	830	1	6.080922
O=C(C(=O)I	0.53	1	9.275724
COc1ccc(-n	70	1	7.154902
C/C(=N\O[(	8	1	8.09691
Oc1ccc(-c2	500	1	6.30103
CC(C)(C)OC	60	1	7.221849
O=C(O)C(S(	100	1	7
CC(c1ccccc	2700	0	5.568636
NS(=O)(=O)	10000	0	5
CC(=O)Oc1	7400	0	5.130768
C=C(C/C=C	1000	0	6
Clc1ccc2nc	60	1	7.221849
Cc1ccc(-c2r	380	1	6.420216
O=C1NCCN	5300	0	5.275724
C/C(=N\OC	9	1	8.045757
Cc1ccc2c(c	6050	0	5.218245
N#Cc1cc(-c	80	1	7.09691
CCC(O)(c1c	2500	0	5.60206
O=C(O)C(S(	800	1	6.09691
O=C1C2CC(	480	1	6.318759
CC(=O)N(C(	900	1	6.045757
O=C(NCCC1	95100	0	4.021819
O=C(O)[C@	42	1	7.376751
O=[N+]([O-	600	1	6.221849
C=C1C(O)C	16.54	1	7.781464
COc1cc([C(	13800	0	4.860121
COc1ccc(-c	170	1	6.769551
C/C=C/c1cc	50000	0	4.30103

OC1(c2cccc	140	1	6.853872
CC(C)c1ccc	260	1	6.585027
C=CCN(CC=	6900	0	5.161151
CCCCCCCCC	105	1	6.978811
CC(C)(Cl)CC	8000	0	5.09691
CS(=O)(=O)	10000	0	5
O=C(O)C[C	2100	0	5.677781
CCOc1ccc2	10	1	8
Cc1ccc(C2=	330	1	6.481486
COc1ccc(/C	110	1	6.958607
CC(Cc1ccc(	25	1	7.60206
CCCCCC(=C	1500	0	5.823909
O=C(OCC#C	30000	0	4.522879
Cc1cc(-c2n	600	1	6.221849
CCCCC(CCC	14	1	7.853872
CC(C)CC(C1	970	1	6.013228
C/C(=N\OC	22	1	7.657577
c1cc2c(cc1	230	1	6.638272
O=c1cc(-c2	102	1	6.9914
O=C1c2ccc	320	1	6.49485
Cc1cnccc1-	430	1	6.366532
O=C(OCC#C	50000	0	4.30103
COc1cc(/C=	200	1	6.69897
CCC(O)(c1c	40000	0	4.39794
CCOc1cc(/C	10000	0	5
CS(=O)(=O)	200	1	6.69897
C[C@H](C#	80	1	7.09691
CCCCCCC(S	3300	0	5.481486
CCCCCCCCC	240	1	6.619789
COc1ccc(C(	4740	0	5.324222
COc1cc(-c2	189	1	6.723538
O=C(N[C@	50000	0	4.30103
C/C(=N\O)c	40	1	7.39794
CC(C#Cc1cc	4000	0	5.39794
CC(C)/C=C/	1670	0	5.777284
CCCCC/C=C	2800	0	5.552842
CCNC(=O)N	24	1	7.619789
COc1ccc(Cc	30	1	7.522879
CC(C#Cc1cc	4000	0	5.39794
COc1cccc(C	725	1	6.139662
CC(c1ccoc1	2100	0	5.677781
CC(C1=Cc2	200	1	6.69897
C/C(=N\OC	360	1	6.443697
COc1ccc(/N	8220	0	5.085128
COC1(c2ccc	300	1	6.522879
Cn1nnc(NC	54	1	7.267606
O=C1OCc2c	5500	0	5.259637
CSc1ccccc1	820	1	6.086186
Cc1cnc2c(S	200000	0	3.69897
CC1Cc2c(O	37	1	7.431798
O=c1c2cccc	10	1	8

CO[C@@](	510	1	6.29243
Cc1c(CC(=C	2000	0	5.69897
Cc1ccc(S(=	170	1	6.769551
CC(/C=C/c1	1200	0	5.920819
COc1cccc(C	100000	0	4
C[C@H](C#	220	1	6.657577
O=C(c1sc(N	290	1	6.537602
Cc1ccccc1C	480	1	6.318759
Cn1ncc(NC	190	1	6.721246
Cc1ccc(N/N	1920	0	5.716699
O=[N+]([O-	6900	0	5.161151
CC(C)=CCC	18000	0	4.744727
CCC1CN(c2	3800	0	5.420216
COc1cc(/C=	50	1	7.30103
O=C(CCOc1	300	1	6.522879
CCCCC(Sc1	500	1	6.30103
CCCCC(Sc1	1600	0	5.79588
O=C(Oc1cc	50000	0	4.30103
C#CCCCC(=	3000	0	5.522879
CCOC(=O)c	182	1	6.739929
COc1cccc(/	800	1	6.09691
CC1=NN(C(	61	1	7.21467
Cc1cc(OCc2	730	1	6.136677
O=C(O)CSC	7	1	8.154902
N=C1/C(=N	2730	0	5.563837
O=c1[nH]n	600	1	6.221849
CCc1ccc(/C	590	1	6.229148
COc1cccc(-	42	1	7.376751
O=C(Oc1cc	8700	0	5.060481
CC(c1ccc(-c	3100	0	5.508638
COc1ccc(-c	13900	0	4.856985
Oc1ccc(-c2	76	1	7.119186
CCCC/C(=C	5700	0	5.244125
COC(=O)c1	4100	0	5.387216
CC1CCC(=C	69180	0	4.160019
O=c1c2ccc	100	1	7
CN1CCn2n	20	1	7.69897
COc1cccc(C	69000	0	4.161151
CC(C#Cc1c	10000	0	5
O=C1OCc2	280	1	6.552842
COc1ccc(C	376	1	6.424812
O=C(O)C(S	500	1	6.30103
CC(C)(C)CN	2900	0	5.537602
S=c1[nH]nc	3710	0	5.430626
CCCCCCCC(	270	1	6.568636
NC(=O)N(C	91	1	7.040959
NC(=O)N(C	48	1	7.318759
CC1(C)OC(=	6000	0	5.221849
Cc1cc(/C=C	54	1	7.267606
COC1c2c(-c	40	1	7.39794
FC(F)(F)c1c	400	1	6.39794

CSc1cccc(N	2200	0	5.657577
O=c1cc(-c2	2900	0	5.537602
O=C(NC12(	100000	0	4
COc1ccc2c(	400	1	6.39794
COC1(c2cc(	30	1	7.522879
CC(c1ccc(O	2620	0	5.581699
CC1Cc2c(S(	11	1	7.958607
Cc1ccc2nc(	600	1	6.221849
CCCCCCOc1	770	1	6.113509
COc1ccc2[r	22500	0	4.647817
O=c1[nH]n	100	1	7
C/C(=N\O[(	70	1	7.154902
COc1ccc(C(	52	1	7.283997
COc1ccc(Cc	2134	0	5.670806
CC1(C)NC(c	6800	0	5.167491
CCCOc1ccc	12000	0	4.920819
CC(C1CC1)I	1700	0	5.769551
CNc1oc(-c2	50000	0	4.30103
Oc1cc(O)cc	240	1	6.619789
CC(C)(C)c1c	2800	0	5.552842
C/C(=N\OC	48	1	7.318759
OC(c1ccc(N	400	1	6.39794
Nc1ccc2oc(	28900	0	4.539102
Clc1ccc(-c2	50	1	7.30103
CC1Cc2c(O	38	1	7.420216
C=C1CCCC2	12.73	1	7.895172
Cc1ccc2nc(	40	1	7.39794
CCCCCNC(=	300	1	6.522879
O=C1OCc2c	250	1	6.60206
COC1=CC(=	450	1	6.346787
CCOC(=O)c	1000	0	6
Cc1ccc(S)c2	200000	0	3.69897
NC(=O)N(C	1400	0	5.853872
CCCCC(Sc1	700	1	6.154902
CC1Cc2c(O	300	1	6.522879
COC1(c2cc(	760	1	6.119186
COc1cc(/C=	530	1	6.275724
CC(C)(C)CN	180	1	6.744727
CC(C#Cc1cc	2000	0	5.69897
COC1(c2cc(	720	1	6.142668
C/C(=N\OC	30	1	7.522879
CCCCc1cc(-	6000	0	5.221849
O=C(O)/C(=	21	1	7.677781
O=C(O)C(Sc	400	1	6.39794
O=C1NCCN	2100	0	5.677781
COC1(c2cc(	480	1	6.318759
NC(=O)N(C	120	1	6.920819
C/C(=N\OC	90	1	7.045757
CC(C)(C)CN	380	1	6.420216
O=C1NCCN	5800	0	5.236572
CCCc1cc(C)	12	1	7.920819

O=C(OCc1c	150	1	6.823909
CCc1cc(/C=	90	1	7.045757
Cc1cc(C(C)I	1800	0	5.744727
Cc1cc(/C=C	45	1	7.346787
CCOC(=O)c	95	1	7.022276
Oc1ccc(-c2	20	1	7.69897
COc1cc(/C=	270	1	6.568636
O=C1CC(O)	390	1	6.408935
CS(=O)(=O)	740	1	6.130768
CC1CN(c2c	3100	0	5.508638
COc1cccc(C	100000	0	4
CN(C)CCCC	290	1	6.537602
CSc1c(CC(C	170	1	6.769551
CC1Cc2c(O	16	1	7.79588
CC(C)(CC(O	10	1	8
CCOC(C)=C	200	1	6.69897
COc1cc(O)c	2500	0	5.60206
COc1cc(/C=	200	1	6.69897
CC(C)(C)c1c	260	1	6.585027
C=C(C)[C@	2	1	8.69897
O=c1[nH]cc	10000	0	5
Cc1ccc(C(C	1000	0	6
COc1ccc2[r	74200	0	4.129596
O=C(NCCC1	4200	0	5.376751
CC1Cc2c(O	33	1	7.481486
COc1ccc(/C	440	1	6.356547
COC1(c2cc	4200	0	5.376751
Clc1ccc(-c2	10000	0	5
CC(C#Cc1cc	70	1	7.154902
COc1ccc(-c	1370	0	5.863279
COC1(c2sc(	140	1	6.853872
Cc1cc(OCc2	160	1	6.79588
Oc1ccc(-c2	30	1	7.522879
Cc1cc(OCc2	80	1	7.09691
COc1ccc(/C	940	1	6.026872
CN(O)C(=O	90	1	7.045757
CCCCC(Sc1	900	1	6.045757
COc1cc(/C=	350	1	6.455932
NS(=O)(=O)	15000	0	4.823909
O=C(O)C(Sc	400	1	6.39794
Oc1cccc(Nc	1200	0	5.920819
Oc1ccc(Nc2	30	1	7.522879
Cc1ccc(/C=	160	1	6.79588
CCCCCCCC(	30	1	7.522879
CCOc1cccc	10000	0	5
NC(=O)N(O	550	1	6.259637
COc1ccccc1	2700	0	5.568636
C/C(=C\CC/	131	1	6.882729
c1ccc(COc2	7500	0	5.124939
Oc1ccc(Nc2	60	1	7.221849
CC1Cc2c(O	55	1	7.259637

NC(=O)N(C	194	1	6.712198
COc1ccc(/C	400	1	6.39794
O=C1OCc2c	140	1	6.853872
Cc1ccc(-c2c	1420	0	5.847712
Oc1c(Cl)cc(	3600	0	5.443697
CCOC1(c2c	80	1	7.09691
C/C(=N\OC	40	1	7.39794
O=c1c2cccc	30	1	7.522879
Cc1cc(/C=C	1000	0	6
C/C=C/c1cc	50000	0	4.30103
CC(c1ccc(O	360	1	6.443697
COC1CCc2c	40000	0	4.39794
CCOC(=O)c	150	1	6.823909
CCOC(=O)c	5500	0	5.259637
Cc1cc(-c2cc	9	1	8.045757
CCCCCC(=C	1300	0	5.886057
CCCCC(Sc1c	800	1	6.09691
COC1(c2ccc	150	1	6.823909
Oc1ccc(Nc2	30	1	7.522879
O=c1[nH]n	600	1	6.221849
COc1cc(/C=	4000	0	5.39794
CN(O)C(=O	87	1	7.060481
CC1Cc2c(O	28	1	7.552842
CCCN(O)C(=	160	1	6.79588
CCCCCCCc1	310	1	6.508638
O=C(O)/C=	27	1	7.568636
O=C(O)c1cc	800	1	6.09691
CC1Cc2c(O	500	1	6.30103
CC1Cc2c(O	300	1	6.522879
COc1cc(/C=	2	1	8.69897
COc1ccc(Cc	99	1	7.004365
O=c1[nH]cc	10000	0	5
O=C(CCc1c	100	1	7
COc1ccc(N	400	1	6.39794
Oc1ccc(-c2	900	1	6.045757
COc1cc2c(c	4200	0	5.376751
C=CCc1cc(C	20	1	7.69897
CC1Cc2c(O	60	1	7.221849
CC(C)Cc1cc	23000	0	4.638272
CC(=O)N(O	38	1	7.420216
CC(C)=CCC	600	1	6.221849
O=c1c2cccc	5	1	8.30103
O=c1c2cccc	100	1	7
CCCCCCCCI	23000	0	4.638272
COC1=CC(=	550	1	6.259637
CC1(C)C=Cc	2400	0	5.619789
COC(=O)CC	580	1	6.236572
O=C(c1ccc(	50	1	7.30103
Cc1ccc(-c2n	350	1	6.455932
O=C1CCOC	26830	0	4.571379
O=C(NCCC(	1100	0	5.958607

COc1cc2oc	480	1	6.318759
CC(C)c1ccc	35	1	7.455932
Cn1c(-c2cc	10000	0	5
COC1(c2cc	80	1	7.09691
Cc1ccc(S(=	1.1	1	8.958607
NC(=O)N(C	740	1	6.130768
CC(C(=O)N	100	1	7
Oc1ccc(-c2	7	1	8.154902
CC(/C=C/c1	850	1	6.070581
COc1cc(/C=	270	1	6.568636
O=C(O)/C=	160	1	6.79588
Cc1ccc(C2=	250	1	6.60206
O=c1oc2cc	24000	0	4.619789
O=C1OC(=(	660	1	6.180456
COC(=O)c1	9800	0	5.008774
O=C(NO)N(	5200	0	5.283997
CCC(O)(c1c	11	1	7.958607
COc1ccc(/C	300	1	6.522879
O=C1OCc2	250	1	6.60206
Nc1ccc(-c2	50	1	7.30103
CCCCC/C=C	2000	0	5.69897
CC[C@](CC	70	1	7.154902
O=C(O)/C=	27	1	7.568636
O=[N+]([O-	3090	0	5.510042
COC1CCC(=	590	1	6.229148
Cc1cccc(N2	1700	0	5.769551
COc1ccc(-c	590	1	6.229148
O=C(O)/C=	17	1	7.769551
O=C(CSc1n	15000	0	4.823909
O=C(Cc1cn	6800	0	5.167491
CN(C)c1ccc	50	1	7.30103
CC(/C=C/c1	80	1	7.09691
O=C1NCCN	200	1	6.69897
CC1Cc2c(O	4000	0	5.39794
Cc1nn(Cc2	65000	0	4.187087
COc1ccc(/C	80	1	7.09691
COc1ccc(-c	400	1	6.39794
COc1cc(-c2	649	1	6.187755
O=C(OCc1c	130	1	6.886057
CC1Cc2c(O	26	1	7.585027
CCCCC/C=C	1100	0	5.958607
O=C(Oc1cc	1000	0	6
CCC(O)(c1c	198	1	6.703335
CC(C)(C)c1	6000	0	5.221849
O=C(NCC12	78	1	7.107905
Cc1cc(OCC	358	1	6.446117
O=C(Nc1cc	600	1	6.221849
CCCCNC(=C	10500	0	4.978811
CCCCC(Sc1	3000	0	5.522879
O=c1cc(-c2	0.5	1	9.30103
Cc1cc(-c2c	270	1	6.568636

O=C(O)c1n	7400	0	5.130768
COC(=O)c1	3400	0	5.468521
CCCCCCCC/	6000	0	5.221849
CCCc1cc(Cl	11	1	7.958607
CCC(O)(c1c	223	1	6.651695
CCc1nc(-c2	5000	0	5.30103
Oc1ccc(-c2	40	1	7.39794
CNS(=O)(=(	25	1	7.60206
Cc1cc(-c2cc	82	1	7.086186
COc1ccc(N1	2810	0	5.551294
CCCC(O)(c1	8800	0	5.055517
FC(F)(F)c1c	600	1	6.221849
COc1ccc(/C	9000	0	5.045757
CCCCc1c(-c	115	1	6.939302
CC1(C)COC	190	1	6.721246
NC(=O)N(C	1360	0	5.866461
COc1ccc(/C	49000	0	4.309804
COc1ccc(S2	680	1	6.167491
COC1(c2cc(	600	1	6.221849
CN1CCC(=C	100000	0	4
CCOC(=O)c	140	1	6.853872
COc1ccc(-c	500	1	6.30103
CSC1(c2cc(	72	1	7.142668
CC(=O)CC1	62380	0	4.204955
CCCCCCCC(	29	1	7.537602
O=C1NCCN	4600	0	5.337242
CC(=O)c1cc	600	1	6.221849
COc1cc(-c2	439	1	6.357535
CC(C)(C)c1c	1900	0	5.721246
Cc1cc(NC(=	680	1	6.167491
CN(C)c1ccc	500	1	6.30103
CC(C)c1ccc	250	1	6.60206
COc1cccc(C	80	1	7.09691
O=C(CCCc1	130	1	6.886057
CC(c1cc2cc	1500	0	5.823909
CC1(C)CCC[	680	1	6.167491
CCOC(=O)c	160	1	6.79588
CCCCCCC(S	1500	0	5.823909
COC(=O)c1	370	1	6.431798
O=C(O)C(Sc	500	1	6.30103
CC(C)(C)CN	10000	0	5
COc1ccc2cc	58000	0	4.236572
COc1ccc(CC	2500	0	5.60206
OCCOc1ccc	8000	0	5.09691
CCCCCCNC	11900	0	4.924453
Cc1cc(-c2cc	170	1	6.769551
O=C(O)/C=	87	1	7.060481
CCCCCCC(S	300	1	6.522879
COc1cc(/C=	4.2	1	8.376751
C=C1CCC[C	200000	0	3.69897
CCCCCCc1c	115	1	6.939302

NC(=O)N(C	3600	0	5.443697
CCOC(=O)C	500	1	6.30103
COc1ccc2c	3600	0	5.443697
C[C@@H]1	830	1	6.080922
C=CCNC(=C	5000	0	5.30103
CC(=O)Oc1	450	1	6.346787
COc1cc(-c2	620	1	6.207608
O=c1oc2cc	21000	0	4.677781
CCOC(=O)/	35	1	7.455932
CNc1nc2c(	100	1	7
CCCCCCCC	220	1	6.657577
Cc1ccc(CN(	2100	0	5.677781
CC(c1cc2cc	1500	0	5.823909
CC1Cc2c(O	105	1	6.978811
C=CCn1cc(	1.8	1	8.744727
COC1(c2cc	200	1	6.69897
CC1Cc2c(O	110	1	6.958607
Cc1nccn1-c	830	1	6.080922
O=C(Cc1cn	2300	0	5.638272
CC(C)C(=O)	13000	0	4.886057
O=C(O)c1n	380	1	6.420216
COC(C)(c1c	500	1	6.30103
C/C(=N\OC	20	1	7.69897
Cc1ccnc(N2	180	1	6.744727
Cn1[nH]c(=	100	1	7
COc1ccc2c	6100	0	5.21467
O=C(c1cccs	380	1	6.420216
[N-]=[N+]=	440	1	6.356547
CC1Cc2c(O	360	1	6.443697
C=C1CCC[C	200000	0	3.69897
NC(=O)N(C	200	1	6.69897
O=C(O)/C=	91	1	7.040959
Cc1nccn1-c	1000	0	6
C[S+]([O-])	770	1	6.113509
COC1(c2cc	40	1	7.39794
O=C(O)C(S	400	1	6.39794
OC1(c2ccc	1460	0	5.835647
CC(C#Cc1c	300	1	6.522879
CCCCCC(=C	460	1	6.337242
Cn1c(=O)c	9900	0	5.004365
COc1cc(/C=	58.4	1	7.233587
CC(=O)C(C)	53450	0	4.272052
COc1cc(Cl)	300	1	6.522879
Brc1ccc(-c2	8400	0	5.075721
C/C(=C\c1c	430	1	6.366532
N#Cc1cc(-c	190	1	6.721246
COc1ccc2c	47400	0	4.324222
OC(c1cccc(	5100	0	5.29243
Cc1ccc(C2=	190	1	6.721246
NC(=O)N(C	120	1	6.920819
CC(C)CC(=C	16000	0	4.79588

COc1ccc(/C	90	1	7.045757
COC1c2c(-c	60	1	7.221849
CC1CN(c2c	5200	0	5.283997
O=C(c1cccs	380	1	6.420216
CNc1oc(-c2	50000	0	4.30103
COc1cc(O)c	2100	0	5.677781
Cc1ccc(S(=O	1700	0	5.769551
O=C(O)/C=	150	1	6.823909
Cc1ccc(C2=	350	1	6.455932
NC(=O)N(C	600	1	6.221849
O=C(O)/C=	14	1	7.853872
C/C(CCC(=O	20	1	7.69897
CC[C@](OC	410	1	6.387216
CCC(O)(c1c	10000	0	5
Cc1cc(-c2cc	33	1	7.481486
COc1ccc(/C	30000	0	4.522879
COc1ccc2cc	2900	0	5.537602
O=C(NCCC(	1900	0	5.721246
Nc1ccc(-c2	400	1	6.39794
CC1(C)Cc2c	30	1	7.522879
Cc1ccc2c(c	10000	0	5
Cc1cc(/C=C	370	1	6.431798
CCCCCCC(S	1400	0	5.853872
CCOC(=O)c	2000	0	5.69897
C=C1CCC[C	25100	0	4.600326
NC(=O)N(C	230	1	6.638272
CC1Cc2c(O	97	1	7.013228
CC(C)(C)CN	10000	0	5
c1ccc(-c2c(	10000	0	5
O=C(c1cccs	380	1	6.420216
O=C(O)[C@	60	1	7.221849
CC(/C(F)=C	90	1	7.045757
COC(=O)N(	90	1	7.045757
Cc1cc(C(O/	20	1	7.69897
O=C(COC(=	1490	0	5.826814
CCOC(=O)c	130	1	6.886057
CCCCC(=O)	12000	0	4.920819
c1ccc2c(c1	97000	0	4.013228
COc1ccc(-c	1593	0	5.797784
CC(C)c1ccc	360	1	6.443697
O=C(O)c1cc	500	1	6.30103
NNC(=O)c1	3500	0	5.455932
COc1ccc2cc	2800	0	5.552842
O=C(O)c1n	10000	0	5
Cc1c(C)c2c	520	1	6.283997
CC1=NN(c2	530	1	6.275724
NS(=O)(=O)	810	1	6.091515
CC[C@H](C	750	1	6.124939
COc1ccc(/C	980	1	6.008774
CC(C)(C)c1c	770	1	6.113509
CC(=O)NNc	65000	0	4.187087

CN(O)C(=O	200	1	6.69897
COCCNC(=(	50000	0	4.30103
COc1ccc2c(	76600	0	4.115771
COC1(c2cc(	400	1	6.39794
O=C(O)Cn1	4.9	1	8.309804
O=C(Nc1cc	240	1	6.619789
O=c1c2ccc(	55.9	1	7.252588
CN(O)C(=O	70	1	7.154902
Cc1ccc(Nc2	700	1	6.154902
O=C(Cc1cc(	120	1	6.920819
Oc1ccccc1N	30	1	7.522879
COC(=O)c1	7900	0	5.102373
O=C(Oc1cc	50000	0	4.30103
NC(=O)N(O	60	1	7.221849
CC(C)=CCc1	900	1	6.045757
Cn1nc(C(F)	5000	0	5.30103
COc1ccc(N(	1100	0	5.958607
COc1ccc2c(	670	1	6.173925
COc1cc(/C=	13900	0	4.856985
CCCCCCCC1	220	1	6.657577
Oc1c(Cc2cn	0.8	1	9.09691
COc1cc(/C=	560	1	6.251812
CCOC(=O)c	110	1	6.958607
O=C(O)/C=	140	1	6.853872
CC(C#Cc1c(	1700	0	5.769551
O=C1OCc2(	320	1	6.49485
CCCCCCCC(	500	1	6.30103
Cc1cc(C)c(-	1600	0	5.79588
N#Cc1cccc(	9300	0	5.031517
CCCCNC(=(	5500	0	5.259637
COC1(c2cc(	3	1	8.522879
Cc1ccc(C2=	1070	0	5.970616
C=CCC(O/N	142	1	6.847712
COc1cccc(/	6100	0	5.21467
CCOC(=O)c	1150	0	5.939302
CC(C)C[C@	10000	0	5
Cc1cc(-c2c(	86	1	7.065502
CCCOc1cc((	800	1	6.09691
COC(=O)c1	3500	0	5.455932
OC1(c2cccc(	70	1	7.154902
CCCCC(CCC	10	1	8
COc1ccc(N(	400	1	6.39794
Cc1ccc2c(c	10000	0	5
Clc1ccc2nc	170	1	6.769551
CCN(C)C(=(	22000	0	4.657577
CN(O)C(=O	250	1	6.60206
CC[C@@]([	154	1	6.812479
COc1cc(/C=	100000	0	4
O=C1CCCC1	98550	0	4.006343
Cc1ccc(S(=(	3500	0	5.455932
CC1Cc2c(O	12	1	7.920819

C/C(CCCC(=	59	1	7.229148
CCC[C@H](	770	1	6.113509
N#Cc1cc(-c	350	1	6.455932
CCCCCCCC(	40	1	7.39794
O=C1CCCN	5500	0	5.259637
CC(C)(C)CN	1420	0	5.847712
CC(C)(C)C(=	15000	0	4.823909
Cn1nnnc1-	3000	0	5.522879
Cc1ccc(C2=	230	1	6.638272
O=C1OCc2(	360	1	6.443697
NS(=O)(=O)	68000	0	4.167491
Cc1nccn1-c	1000	0	6
O=C1c2c(O	7000	0	5.154902
COc1cc(/C=	800	1	6.09691
COC(=O)[C	1.01	1	8.995679
O=C1N/C(=	54000	0	4.267606
CCCCCCC1=	330	1	6.481486
O=c1cc(-c2	100	1	7
CC(c1ccccc	30	1	7.522879
CC(=O)Nc1	1340	0	5.872895
CC(C)(C)Sc1	1.6	1	8.79588
Cc1cc(-c2cc	22	1	7.657577
C/C(=N\OC	120	1	6.920819
COC1(c2cc(	800	1	6.09691
CCn1c(=O)(	4.5	1	8.346787
CC(C)C(=O)	1800	0	5.744727
CCc1cccc(N	3000	0	5.522879
O=c1c(O)c(	100	1	7
CCOC(=O)N	72	1	7.142668
COc1cc(/C=	400	1	6.39794
NC[C@H](C	10000	0	5
CN(O)C(=O	200	1	6.69897
O=C(Nc1cc	1130	0	5.946922
COC(=O)c1	770	1	6.113509
CCC(=O)NC	100000	0	4
CCCCCNC(=	300	1	6.522879
Cn1c(=O)cc	440	1	6.356547
CCCCCCC1=	320	1	6.49485
COC1=CC(=	100	1	7
Cc1cc(-c2cc	20	1	7.69897
CCOC(=O)c	220	1	6.657577
C/C(=C\CC/	210	1	6.677781
Fc1ccc(CN2	1000	0	6
Cc1ccsc1CN	1100	0	5.958607
CCCCCCCC(	790	1	6.102373
CCn1ncc(N	29	1	7.537602
O=C(CCCC(	6000	0	5.221849
COC1(c2cn	500	1	6.30103
NC(=O)N(C	1800	0	5.744727
CN(O)C(=O	20	1	7.69897
O=C(/N=C1	360	1	6.443697

O=C1N=C(	300	1	6.522879
O=C1NC(O	2600	0	5.585027
Cc1cc(/C=C	100	1	7
CCCC(=O)c	8500	0	5.070581
CC(C)(C)c1	5550	0	5.255707
CCCCC/C=C	1000	0	6
COc1ccc2c	7100	0	5.148742
COC1(c2cc	850	1	6.070581
CCCCCC(=C	100000	0	4
NC(=O)N(C	70	1	7.154902
NC(=O)N(C	15000	0	4.823909
COC(=O)CC	6000	0	5.221849
CC(C)CCCC	15000	0	4.823909
Cc1ccc(S(=	1500	0	5.823909
CC1Cc2c(O	100	1	7
NC(=O)N(C	117	1	6.931814
CCCCC(Sc1	20	1	7.69897
O=C(NC(=S	0.34	1	9.468521
CCOC(=O)c	31	1	7.508638
O=c1[nH]n	600	1	6.221849
CCCCCCCC	260	1	6.585027
CNC(=O)C1	280	1	6.552842
Cn1nnc(-c2	80	1	7.09691
N#Cc1cc(-c	1000	0	6
O=C(O)c1n	6100	0	5.21467
CCCCCCCC	270	1	6.568636
OC(C#CCO	100000	0	4
COc1ccc(/C	600	1	6.221849
N#Cc1ccc(-	380	1	6.420216
CC1(C)OCC	350	1	6.455932
O=[N+]([O-	50000	0	4.30103
O=C/C=C/c	35000	0	4.455932
O=C(Nc1cc	1800	0	5.744727
N#Cc1cc(-c	3000	0	5.522879
O=C(c1cccs	380	1	6.420216
CN1C(=O)C	40	1	7.39794
CCCCCCCC	1020	0	5.9914
Cc1ccnc2c(	200000	0	3.69897
Cn1nccc1-c	229	1	6.640165
COc1ccc(/C	30000	0	4.522879
CCOC(=O)c	370	1	6.431798
Cc1occc1C	460	1	6.337242
COc1cc(/C=	230	1	6.638272
O=c1c2ccc	57.9	1	7.237321
CCCCCCC(S	400	1	6.39794
O/N=C1C(=	27000	0	4.568636
O=C1OCc2	88	1	7.055517
COc1cc(O)	4010	0	5.396856
O=C(O)/C=	30	1	7.522879
Cc1cc(NC(=	720	1	6.142668
C=CCNC(=C	37	1	7.431798

CCCOc1cc(	800	1	6.09691
O=c1c2ccc	100	1	7
NS(=O)(=O)	1780	0	5.74958
C/C(=N\OC	15	1	7.823909
Cc1c(C)c2c	130	1	6.886057
Cc1cc(/C=C	1000	0	6
Cn1c(-c2cc	650	1	6.187087
C=C(C/C=C	1100	0	5.958607
CC(C#Cc1cc	3100	0	5.508638
CC(C)(C)CN	10000	0	5
O=C(O)CCC	107	1	6.970616
C/C=C/c1cc	50000	0	4.30103
Cc1cc(OCC	820	1	6.086186
CC(=O)c1cc	15300	0	4.815309
Cc1ccc(C(C	3540	0	5.450997
O=C(/N=c1	380	1	6.420216
O=C1CCCC	130500	0	3.884389
O=C(c1cccc	70	1	7.154902
O=c1cc(-c2	11.6	1	7.935542
Cc1cc(S)c2n	200000	0	3.69897
CC(=O)c1cc	390	1	6.408935
CCCCNC(=C	65	1	7.187087
CS(=O)(=O)	10000	0	5
C[C@H](C#	230	1	6.638272
COC1CC(O)	23	1	7.638272
CC1CN(c2c	13000	0	4.886057
CS(=O)(=O)	10000	0	5
COc1cc(-c2	614	1	6.211832
Cn1c2c(c3c	1200	0	5.920819
O=C(CCCc1	130	1	6.886057
O=C(O)c1cc	2290	0	5.640165
CCOC(=O)c	1860	0	5.730487
CC(C)COC(=	176	1	6.754487
CCC(O)(c1c	55	1	7.259637
CC(c1ccccc	1400	0	5.853872
CCCCC(=O)	800	1	6.09691
COc1ccccc1	600	1	6.221849
Cc1cc(-c2cc	37	1	7.431798
CC(C)C(O)(	40000	0	4.39794
O=C(Nc1cc	280	1	6.552842
C[C@H](C#	260	1	6.585027
COc1ccc(/C	100000	0	4
CCC(O)(c1c	40000	0	4.39794
COc1ccc(N	1100	0	5.958607
CC(C)(C)CN	1020	0	5.9914
N#Cc1c(-c2	4330	0	5.363512
CN(O)C(=O	39	1	7.408935
O=c1cc(-c2	3.7	1	8.431798
Cc1cc(-c2cc	140	1	6.853872
CC(=O)C1(c	130	1	6.886057
Cc1nn2c(c1	4.8	1	8.318759

CCOC(=O)c	250	1	6.60206
O=C(O)c1c(	50000	0	4.30103
CC(C)CC(=C	1300	0	5.886057
Cc1cc(-c2c(	90	1	7.045757
C/C(=N\OC	20	1	7.69897
CCCCCCCC(	500	1	6.30103
C[S+]([O-])(	5000	0	5.30103
COc1ccc(/C	91	1	7.040959
COCCOCC1	24000	0	4.619789
C[C@@H]1	50000	0	4.30103
O=C(NO)c1	120	1	6.920819
CC[C@](O)	65	1	7.187087
CC1(C)CCC|	50000	0	4.30103
CNC(=O)c1	230	1	6.638272
CC[C@](O)	10000	0	5
CCOC(=O)c	84	1	7.075721
CC(/C=C\c1	160	1	6.79588
O=C(O)/C=	21	1	7.677781
COC1=CC(=	660	1	6.180456
CC(C)Cc1cc	3200	0	5.49485
COc1cc(-c2	696	1	6.157391
Cc1ccc2nc(	192	1	6.716699
C#CCn1cc((	3.1	1	8.508638
Brc1ccc2nc	150	1	6.823909
COc1ccc(C(	200	1	6.69897
Oc1cc(O)cc	18490	0	4.733063
C[C@H](C#	900	1	6.045757
CCOC(=O)c	240	1	6.619789
CC12CC3C(	100000	0	4
CC[C@](O)	28	1	7.552842
Cc1c(C)c2c	90	1	7.045757
COC1(c2cc(	2500	0	5.60206
COc1ccc2c(	180	1	6.744727
COc1cc(/C=	13.5	1	7.869666
COc1ccc2c(	10000	0	5
C/C=C/c1c(	50000	0	4.30103
O=C(Nc1cc	30	1	7.522879
Cc1ccc(C(=	1700	0	5.769551
COc1ccc(CI	1700	0	5.769551
O=C(O)/C((	20	1	7.69897
O=C1NCCN	4600	0	5.337242
CC(=O)Oc1	6100	0	5.21467
CS(=O)(=O)	850	1	6.070581
CC(C)(C)c1(	8000	0	5.09691
C[C@@H](	200	1	6.69897
N#Cc1cc(-c	160	1	6.79588
Cn1c(=O)cc	1100	0	5.958607
CCCCc1ccc(	42	1	7.376751
COc1ccc(-c	0.38	1	9.420216
Cc1cc(CN(C	380	1	6.420216
O=C(O)Cn1	2.5	1	8.60206

CCOC(=O)c	45	1	7.346787
COc1cc(OC	800	1	6.09691
COCc1cc(-c	160	1	6.79588
CC(c1ccc(B	420	1	6.376751
CO/C(C)=C/	1600	0	5.79588
CCCc1sc(-c	5100	0	5.29243
Cc1ccc2nc(	30	1	7.522879
CC(C)(O)c1	1500	0	5.823909
O=C1OCc2c	27	1	7.568636
CCCCCCC(S	600	1	6.221849
CCOc1ccc(S	29000	0	4.537602
COc1ccc(Cc	76	1	7.119186
COC1=CC(=	120	1	6.920819
COc1cc(CC	590	1	6.229148
O=C1N=C(c	110	1	6.958607
CC(C1CC1c	1200	0	5.920819
COC1(c2cc	30000	0	4.522879
CC(C#Cc1cc	160	1	6.79588
Cc1nccn1-c	1000	0	6
CCCCC/C=C	6	1	8.221849
CC[C@@](	340	1	6.468521
C[S+]([O-])c	80	1	7.09691
c1ccc(-c2c(	10000	0	5
O=C1OCc2c	4000	0	5.39794
O=C1CCCN	700	1	6.154902
CCC(OC)(c1	40000	0	4.39794
C/C(=N\O)c	40	1	7.39794
CN(C)S(=O)	210	1	6.677781
COC1=CC(=	360	1	6.443697
Cc1ccc(S(=	10000	0	5
O=C(O)CCC	3	1	8.522879
O=C(NCCC(	100	1	7
N#Cc1cc(-c	15	1	7.823909
O=C(O)c1cc	300	1	6.522879
COc1ccc(/C	2900	0	5.537602
CCCCCCC(S	199.2	1	6.700711
CC[C@@]1	1900	0	5.721246
CCOC(=O)c	1240	0	5.906578
CCCc1cc(Cl	105	1	6.978811
CC1Cc2c(O	165	1	6.782516
CS(=O)(=O)	10000	0	5
CS(=O)(=O)	10000	0	5
NC(=O)N(C	1700	0	5.769551
CC(C)(C)CN	410	1	6.387216
O=C1OCc2c	160	1	6.79588
O=C1CCCN	1800	0	5.744727
CCCCCCCC(	320	1	6.49485
COC(=O)/C	180	1	6.744727
CCCc1c(OC	18900	0	4.723538
CCCCCCC(S	400	1	6.39794
O=c1[nH]n	600	1	6.221849

COc1ccc(O	10000	0	5
CC(C#Cc1c	1300	0	5.886057
COC1(c2nc	500	1	6.30103
NC(=O)N(O	420	1	6.376751
CCc1ccc(N2	125000	0	3.90309
Clc1ccc(-c2	37500	0	4.425969
CCOC(=O)c	170	1	6.769551
CCC(O)(c1c	40	1	7.39794
CCC(OC)(c1	50	1	7.30103
Cc1cc(Cl)cc	44000	0	4.356547
O=C1OCc2	140	1	6.853872
O=C(O)c1c	2000	0	5.69897
CCC1Cc2c(	33	1	7.481486
O=C(Nc1cc	420	1	6.376751
O=C(NCCC(	2200	0	5.657577
CCOC(=O)c	130	1	6.886057
CCOc1cc(/(	860	1	6.065502
CCC(O)(c1c	11000	0	4.958607
O=C(OCC#(	75000	0	4.124939
COc1cc(/C=	2300	0	5.638272
COc1ccc(-c	36000	0	4.443697
CCCCCC(=C	2900	0	5.537602
CCOC(=O)c	237	1	6.625252
CC1Cc2c(O	11	1	7.958607
O=C1CCCN	2500	0	5.60206
O=C(/C=C/	4100	0	5.387216
C[C@H](C#	110	1	6.958607
COc1cccc(	7600	0	5.119186
O=c1cc(-c2	2000	0	5.69897
C/C(=N\OC	50	1	7.30103
C/C(=N\OC	16	1	7.79588
CC(C)c1ccc	1100	0	5.958607
CC(=O)Oc1	59.8	1	7.223299
Cc1ccc2nc(	7200	0	5.142668
C/C(=N/OC	170	1	6.769551
O=c1c(/C=I	28500	0	4.545155
O=C(O)COc	6300	0	5.200659
COC1(c2cc	20	1	7.69897
CC1Cc2c(C	3	1	8.522879
CN(C)Cc1c	80	1	7.09691
COc1cc(/C=	300	1	6.522879
CCCCCCCC(	490	1	6.309804
CC(O)c1cc(	520	1	6.283997
CCOC(=O)c	2760	0	5.559091
COC1(c2cc	400	1	6.39794
COc1ccc(N	12300	0	4.910095
COc1ccc(C(	72000	0	4.142668
N#Cc1ccc(-	100	1	7
CC(C#Cc1c	150	1	6.823909
CCCCC(Sc1	900	1	6.045757
Cc1cccc(NC	1870	0	5.728158

Oc1cccc(N(	3700	0	5.431798
COc1ccc(-c	1500	0	5.823909
Clc1ccc(-c2	2300	0	5.638272
CC(c1nc2cc	520	1	6.283997
COC(C)(c1r	40000	0	4.39794
O=C1C(Cc2	2.4	1	8.619789
CC1CN(c2c	3600	0	5.443697
Cc1ccc(-c2(	380	1	6.420216
COC1=CC(=	490	1	6.309804
O=C1O[C@	5600	0	5.251812
OC1(c2ccc(	640	1	6.19382
CCOC(=O)c	300	1	6.522879
NC[C@H](	10000	0	5
CC(c1cc2n(	860	1	6.065502
CCOC(=O)c	130	1	6.886057
COc1c(C(C)	1300	0	5.886057
O=C(NCc1c	2700	0	5.568636
CCOC(=O)C	4.5	1	8.346787
NC(=O)N(C	150	1	6.823909
COc1ccc(-c	400	1	6.39794
CCC(OCCc1	8	1	8.09691
CSc1ccc(-c2	580	1	6.236572
O=C1OCc2(	26	1	7.585027
Cn1ncc(Cl)(	5400	0	5.267606
CCCc1cc(Cl	13	1	7.886057
Cc1c(-c2cc(	230	1	6.638272
O=C(NC1C(	100000	0	4
NC(=O)N(C	900	1	6.045757
Cc1ccc(C2=	1580	0	5.801343
CC(C)(Cc1c	100	1	7
O=C1c2ccc	150	1	6.823909
CCCCCCOc1	1350	0	5.869666
O=C(Nc1cc	50000	0	4.30103
C#CCCCC(=	6000	0	5.221849
O=C(NCCC(	10000	0	5
O=C(NCCC(	15100	0	4.821023
COc1cc(/C=	240	1	6.619789
COC(=O)c1	6500	0	5.187087
COC1=CC(=	150	1	6.823909
CCCC/C(=C	500	1	6.30103
COc1ccc(/C	220	1	6.657577
O=C(CCCC(	50	1	7.30103
O=C(O)c1c(	610	1	6.21467
NC(=O)N(C	96	1	7.017729
CC1Cc2c(O	66	1	7.180456
CS(=O)(=O)	6200	0	5.207608
CCC(OC)(c1	600	1	6.221849
C[C@H](c1	1100	0	5.958607
Cc1ccc(C2=	300	1	6.522879
CCCCCCCC(	135	1	6.869666
O=c1cc(-c2	3	1	8.522879

Cc1ccc(C2=	520	1	6.283997
COc1ccc(-c	15000	0	4.823909
CCOC(=O)c	1790	0	5.747147
N#Cc1ccc(-	380	1	6.420216
CC(C#Cc1c	600	1	6.221849
NC(=O)N(C	60	1	7.221849
CCOC(=O)c	30000	0	4.522879
Cc1nc(-c2c	1320	0	5.879426
CCC(OC)(c1	4800	0	5.318759
Oc1ccc(-c2	210	1	6.677781
O=C(O)C(S	300	1	6.522879
O=C(NC12	100000	0	4
CC(=O)O[C	750	1	6.124939
C/C(=C\CC/	362	1	6.441291
CCCCCCCC	270	1	6.568636
O=c1[nH]n	600	1	6.221849
CCOC(=O)c	190	1	6.721246
CCCCCOc1	8.65	1	8.062984
COc1ccc2c	1000	0	6
CCOC(=O)c	97500	0	4.010995
Cc1ccc2nc(	80	1	7.09691
N#C/C(=C(/	53000	0	4.275724
NCc1ccc(-c	50	1	7.30103
CC(c1cc2cc	1200	0	5.920819
CC[C@](OC	220	1	6.657577
O=C(NCc1c	14400	0	4.841638
Cc1ccc2nc(	600	1	6.221849
CCCCCC1=C	1010	0	5.995679
C/C(=N\OC	27	1	7.568636
Fc1ccc(CN2	570	1	6.244125
CCOC(C)=C	200	1	6.69897
Cc1ccc(/C=	880	1	6.055517
CCCCCCCC	13	1	7.886057
CCCOC(=O)	2200	0	5.657577
CC(C)=CC/	2460	0	5.609065
CCCc1sc(-c	4500	0	5.346787
Cc1nc(NC(=	0.42	1	9.376751
O=C1NCCN	4800	0	5.318759
O=C(NCCC	900	1	6.045757
COC1=CC(=	380	1	6.420216
Cc1ccnc(N2	2800	0	5.552842
O=C(O)c1c	1100	0	5.958607
COc1ccc(/C	8100	0	5.091515
CCCCC1=C(	3300	0	5.481486
CCOC(=O)c	1400	0	5.853872
Cc1cc(C(C)	1700	0	5.769551
CCCCCCCC	590	1	6.229148
C[C@H](C#	90	1	7.045757
COc1cc(/C=	86	1	7.065502
CCN(Cc1cc	171	1	6.767004
O=c1c2ccc	100	1	7

CC(C)(C)c1c	300	1	6.522879
O=C1OCc2	115	1	6.939302
COc1ccc(-c	720	1	6.142668
O=C(Nc1cc	4	1	8.39794
O=C(Cc1cc	100	1	7
COC1(c2cc	20	1	7.69897
C/C(=N\OC	50	1	7.30103
O=C(O)c1n	65	1	7.187087
Cc1c(C(=O)	5910	0	5.228413
COc1ccc(/C	4620	0	5.335358
COc1ccc(/C	100000	0	4
CCCC/C(=C	900	1	6.045757
Oc1ccc(-c2	7	1	8.154902
COC1(c2cc	60	1	7.221849
CC1Cc2c(O	23	1	7.638272
Oc1ccc(-c2	600	1	6.221849
CCOC(=O)c	67	1	7.173925
CC(C#Cc1c	3500	0	5.455932
CCCC/C(=C	1700	0	5.769551
COc1ccc(-c	2700	0	5.568636
COc1cc(/C=	2	1	8.69897
CCOC(=O)c	2190	0	5.659556
CCOC(=O)c	1260	0	5.899629
O=C(Nc1cc	2237	0	5.650334
CC(=O)NC(	100000	0	4
O=c1cc(-c2	1600	0	5.79588
CSc1ccc(-c	2770	0	5.55752
CC(C)(C)SC	120	1	6.920819
CC(=O)c1cc	19000	0	4.721246
CCOC(=O)c	460	1	6.337242
CCCCCCCC	60	1	7.221849
O=C(O)Cc1	3700	0	5.431798
CC(c1cccs1	1800	0	5.744727
CCC(O)(c1c	88	1	7.055517
O=c1c2ccc	10	1	8
C[S+]([O-])	80	1	7.09691
Oc1cc(O)c(	4400	0	5.356547
Cc1ccc2c(c	10000	0	5
CCOc1ccc2	1800	0	5.744727
O=c1[nH]n	600	1	6.221849
COc1ccc(C	46	1	7.337242
CCOc1cc(/C	1250	0	5.90309
CCCCCCC(S	800	1	6.09691
CCOC(=O)c	130	1	6.886057
CC(C)(C)c1c	9000	0	5.045757
CC(C)=CCc1	2350	0	5.628932
O=C(NCc1c	440	1	6.356547
COc1ccc(C	860	1	6.065502
COc1cccc(N	240	1	6.619789
COC1(c2cc	1300	0	5.886057
C/C(CCCCC	17	1	7.769551

COc1cc(-c2	336	1	6.473661
CCOC(=O)c	1060	0	5.974694
N#Cc1cc(-c	36	1	7.443697
O=[N+]([O-	800	1	6.09691
CCN(Cc1cc	65	1	7.187087
O=C(OCC#	75000	0	4.124939
CCCCCCCC	6300	0	5.200659
NC(=O)N(C	4000	0	5.39794
CC(c1ccc(/	710	1	6.148742
C/C(=N\OC	33	1	7.481486
CCCCCCCC	170	1	6.769551
C/C(=N\OC	490	1	6.309804
CCOC(=O)c	2030	0	5.692504
CC1(C)CCC	4600	0	5.337242
COC(=O)Nc	89000	0	4.05061
CC(C#Cc1c	1700	0	5.769551
CCOC(=O)c	700	1	6.154902
O=C(c1cccs	380	1	6.420216
O=C1OCc2	70	1	7.154902
Cc1onc(NC	160	1	6.79588
CC(C)(C)NC	10000	0	5
CC(C)=CCc1	100000	0	4
CCCCCCCC	680	1	6.167491
CCOc1ccc2	390	1	6.408935
NC[C@H](	9300	0	5.031517
CCCCCCCC	180	1	6.744727
CCCNC(=O)	34	1	7.468521
CC(C)NC(=	49	1	7.309804
CC(C)Cc1cc	600	1	6.221849
COC1(c2cc	50	1	7.30103
O=C1NCCN	8600	0	5.065502
C[C@H](C(	800	1	6.09691
Nc1nc(-c2c	10000	0	5
CCCC/C(=C	6700	0	5.173925
COc1ccc(C	2	1	8.69897
O=C1CCCC	36000	0	4.443697
CC(c1ccc(-c	1400	0	5.853872
O=c1cc(-c2	0.5	1	9.30103
O=c1c2ccc	15	1	7.823909
CC1Cc2c(O	40	1	7.39794
CC1Cc2c(O	19	1	7.721246
COc1cc(/C=	3000	0	5.522879
CC(C)C(=O)	1600	0	5.79588
O=C(Nc1cc	360	1	6.443697
Cc1cc(OCc2	230	1	6.638272
Cn1c(=O)cc	2300	0	5.638272
O=[N+]([O-	470	1	6.327902
CCCCCCCC	140	1	6.853872
Cc1cc(Nc2r	14.4	1	7.841638
CCCCCCCC	210	1	6.677781
COc1cc(/C=	0.25	1	9.60206

COC1(c2cc(	200	1	6.69897
O=c1c2ccc(	8	1	8.09691
OCc1cc2cc1	2200	0	5.657577
COc1cc(/C=	600	1	6.221849
CN(O)C(=O	180	1	6.744727
COc1cc(O)c	300	1	6.522879
O=C(O)/C(C	43	1	7.366532
COc1cc(-c2	190	1	6.721246
NC[C@H](C	5600	0	5.251812
CC(C)(C)c1c	5880	0	5.230623
COc1cccc(C	28	1	7.552842
CCOc1ccc(/	500	1	6.30103
Cc1cc(NC(=	10000	0	5
Cc1ccc(/C=	290	1	6.537602
COc1ccc(N(	34	1	7.468521
COc1cc(-c2	191	1	6.718967
O=[N+]([O-	700	1	6.154902
C/C(=N\OC	13	1	7.886057
O=C(NO)c1	250	1	6.60206
NS(=O)(=O)	12590	0	4.899974
CCC(=O)NC	100000	0	4
C/C(=C1/SC	10000	0	5
N#Cc1ccc(-	170	1	6.769551
O=C(O)/C=	490	1	6.309804
CCCCCc1cc	3720	0	5.429457
CCCc1cc(Cl	30	1	7.522879
CCCC/C(=C	2300	0	5.638272
Cc1cc(OCC(	526	1	6.279014
NC(=O)N(O	37	1	7.431798
CC(C)(C)c1c	7500	0	5.124939
Cn1cc(NC(=	73	1	7.136677
CC1Cc2c(O	27	1	7.568636
CCOC(=O)c	5600	0	5.251812
CCCCCCOc1	350	1	6.455932
O=C(NO)N(	24500	0	4.610834
CC(C)(C#Cc	150	1	6.823909
Cc1ccc2nc(	5	1	8.30103
COc1ccccc1	100	1	7
Cc1ccc(COc	5900	0	5.229148
O=C(/C=C/(	970	1	6.013228
O=C(O)Cc1	5000	0	5.30103
Cc1cc(OCC(	2215	0	5.654626
O=C(O)/C=	143	1	6.844664
COc1ccc2cc	420	1	6.376751
CCCOc1cc(	900	1	6.045757
CC(C)C(=O)	730	1	6.136677
Cc1cc(C)n(-	1000	0	6
COC(=O)[C(	1.2	1	8.920819
Cc1cc(OCC(	1065	0	5.97265
Cc1ccc(C(=	860	1	6.065502
CCCCC(Sc1	800	1	6.09691

O=C(NC1C(	11300	0	4.946922
Cc1ccc(N2(	3500	0	5.455932
CC(C)(C)NC	4400	0	5.356547
COC1(c2cc(	300	1	6.522879
CCCCc1ccc(	200	1	6.69897
COC(=O)[C	6.67	1	8.175874
Cc1cccc(/C	130	1	6.886057
Cc1cc(-c2c(	70	1	7.154902
COc1ccc(/C	20000	0	4.69897
COc1cc(-c2	842	1	6.074688
O=C1OCc2(	450	1	6.346787
COc1cccc(C	35000	0	4.455932
COc1ccc(-c	17000	0	4.769551
O=C(c1cccs	380	1	6.420216
c1ccn2c(NC	420	1	6.376751
CCCc1cc(/C	86	1	7.065502
CCCC[C@H	2000	0	5.69897
CCC(OC)(c1	160	1	6.79588
CC1=NN(C(	110	1	6.958607
O=C1OCc2(	22	1	7.657577
COc1cccc(N	7100	0	5.148742
COC1(c2cc(	10600	0	4.974694
CCCCCCCC(	1250	0	5.90309
Cn1[nH]c2(	1	1	9
Cc1cc(-c2c(	8.6	1	8.065502
CC1Cc2c(O	30	1	7.522879
CC(c1ccc(C	680	1	6.167491
COc1ccc(Cn	28	1	7.552842
NC(=O)N(C	89	1	7.05061
CC1Cc2c(O	70	1	7.154902
Brc1ccc(CS	100000	0	4
O=C(c1cc(-	50	1	7.30103
COC(=O)c1	3500	0	5.455932
Cc1cc(-c2c(	3.9	1	8.408935
COc1ccc2c(	38000	0	4.420216
CC(=O)N(O	770	1	6.113509
Cc1nccn1-c	2000	0	5.69897
O=C(O)/C=	1200	0	5.920819
COC1=CC(=	1670	0	5.777284
Cc1ccc(C2=	330	1	6.481486
O=C(O)c1c(	50000	0	4.30103
CC(=O)N(C)	6210	0	5.206908
COc1cc(OC	10000	0	5
COc1ccc(-c	5000	0	5.30103
COc1cc(/C=	13900	0	4.856985
CCCCC(Sc1(	1600	0	5.79588
CCCCCC(=C	3100	0	5.508638
COc1ccc(/C	10280	0	4.988007
CC1Oc2ccc	87	1	7.060481
C/C(=N\OC	30	1	7.522879
CC(C)(C)CN	10000	0	5

NC(=O)c1[r	35	1	7.455932
COc1cc(CN	56000	0	4.251812
O=C(OCC#(	10000	0	5
COc1cccc(-	590	1	6.229148
O=c1c2ccc(	58.9	1	7.229885
O=S(=O)(c1	2800	0	5.552842
CC(c1sc2cc	3700	0	5.431798
CC1Cc2c(O	20	1	7.69897
CN(O)C(=O	20	1	7.69897
N=c1c2c(nc	1930	0	5.714443
CCC(C#Cc1	500	1	6.30103
O=[N+]([O-	50000	0	4.30103
COC1(c2cc(	20	1	7.69897
CCOC(=O)c	94	1	7.026872
Fc1ccc(CN2	160	1	6.79588
O=C(O)C1C	67	1	7.173925
CN(C)c1ccc	350	1	6.455932
CCCCCCCC(	270	1	6.568636
CC1Cc2c(O	210	1	6.677781
N#Cc1cccc(	16300	0	4.787812
COc1cc2oc	30000	0	4.522879
Cc1ccccc1N	8200	0	5.086186
O=C1OC(c2	80000	0	4.09691
CCC(O)(c1c	3.7	1	8.431798
O=C(O)/C=	56	1	7.251812
C/C(=N\OC	1400	0	5.853872
Cc1ccc(C2=	2690	0	5.570248
CCCCn1c(C	95	1	7.022276
CCCCCCC(S	400	1	6.39794
COc1cccc(C	200000	0	3.69897
COc1ccc(/C	1950	0	5.709965
O=C1NCCN	37000	0	4.431798
O=C(Oc1cc	7100	0	5.148742
CCCCCCCC(	50	1	7.30103
O=C1OC(O	6000	0	5.221849
CC(C#Cc1c(	900	1	6.045757
COc1cc(OC	3700	0	5.431798
CN(O)C(=O	85	1	7.070581
CC(C)(C)CN	10000	0	5
O=C(Cc1cn	2900	0	5.537602
C=CCSCC=C	83000	0	4.080922
N=C1/C(=N	2780	0	5.555955
CN(C)CCNC	3000	0	5.522879
O=C1OCc2(	3800	0	5.420216
Cc1c(CC(=C	900	1	6.045757
CCCCCC[C@	400	1	6.39794
CC(C)(C)c1(	5000	0	5.30103
CCOc1ccc2	110	1	6.958607
CCCC1Oc2(	25	1	7.60206
C/C(=N\O)(	90	1	7.045757
Cc1cc(NC(=	46	1	7.337242

O=C1NC(O	2600	0	5.585027
CCCCC(Sc1	700	1	6.154902
CCOC(=O)c	130	1	6.886057
CCCCCCc1c	110	1	6.958607
CN1CCC(S(	740	1	6.130768
C/C(=N\OC	33	1	7.481486
COc1ccc(/C	130	1	6.886057
CC(C)(C)c1c	5000	0	5.30103
Oc1ccc([C@	5900	0	5.229148
CC1CN(c2c	5500	0	5.259637
CN(c1ccc(S	160	1	6.79588
CC(=O)NC(	100000	0	4
COc1ccc2[r	100000	0	4
O=C(O)/C=	25	1	7.60206
CC1Cc2c(O	130	1	6.886057
Oc1ccc(-c2	10	1	8
CC(C)C(C#C	500	1	6.30103
C/C(=C\CC/	175	1	6.756962
COC1(c2cc	10	1	8
Clc1ccc(-c2	700	1	6.154902
CN(O)C(=O	130	1	6.886057
O=C(O)c1c	1470	0	5.832683
N#Cc1cc(-c	100	1	7
O=C1NCCn	8.1	1	8.091515
CC1CC(O)(	180	1	6.744727
CCCCCCCC(	28	1	7.552842
CCOC(=O)c	270	1	6.568636
COC1=CC(=	170	1	6.769551
C/C(=N\O)c	130	1	6.886057
COc1cccc(C	100000	0	4
NC[C@H](C	1400	0	5.853872
C[C@H](C#	96	1	7.017729
CCCCCC(=C	100000	0	4
O=C1C(Cc2	1.1	1	8.958607
Cc1nc(COc	380	1	6.420216
CC(=O)N1C	700	1	6.154902
CC(C)(C)CN	9020	0	5.044793
CCOC(=O)[	920	1	6.036212
COc1ccc(-c	1107	0	5.955852
c1ccc2c(c1	20	1	7.69897
O=[N+]([O-	50000	0	4.30103
CCOC(=O)c	782	1	6.106793
CCOC(=O)c	370	1	6.431798
O=C(/C=C/c	16.8	1	7.774691
CCCCCCCC(	30	1	7.522879
COc1ccc(/C	100000	0	4
CCOC(=O)c	97	1	7.013228
O=C1C(Cc2	4.2	1	8.376751
Cc1ccccc1C	1900	0	5.721246
COc1cccc(/	480	1	6.318759
COc1cc(/C=	2800	0	5.552842

NS(=O)(=O)	4960	0	5.304518
Cc1coc(NC	210	1	6.677781
CN1C[C@@	19600	0	4.707744
CCOC(=O)c	1600	0	5.79588
O=C(Nc1cc	30	1	7.522879
O=C1C(C(c	1800	0	5.744727
O=c1c2ccc	15	1	7.823909
CC(C)C(C1=	3200	0	5.49485
O=c1c(O)c(	2700	0	5.568636
O=C1OCc2	380	1	6.420216
CCOC(=O)c	3300	0	5.481486
CC(C#Cc1c	120	1	6.920819
CNc1nnc(C	60600	0	4.217527
NC(=O)N(C	310	1	6.508638
N#Cc1cc(-c	124	1	6.906578
CC(C#Cc1c	160	1	6.79588
C[C@@H](	500	1	6.30103
COc1cc(/C=	13900	0	4.856985
CCCCCCCC	330	1	6.481486
CC(C)C(=O)	6700	0	5.173925
Clc1ccc(-c2	900	1	6.045757
CCCOC(=O)	710	1	6.148742
COc1cccc1	204000	0	3.69037
N/C(=N/O)	190	1	6.721246
NC(=O)C1(	270	1	6.568636
O=c1c2ccc	20	1	7.69897
CCC(O)(c1c	7.9	1	8.102373
CCOC(=O)c	3400	0	5.468521
CC1Cc2c(O	20	1	7.69897
CN(O)C(=O	530	1	6.275724
NC(=O)N(C	700	1	6.154902
CCOC(=O)c	1200	0	5.920819
O=c1oc(-c2	0.64	1	9.19382
Cc1ccncc1C	5	1	8.30103
COc1cc(-c2	473	1	6.325139
Cc1nccn1-c	810	1	6.091515
C[C@@H](	50	1	7.30103
COc1cc(-c2	558	1	6.253366
CCCCC/C=C	1700	0	5.769551
COc1ccc2c	1.96	1	8.707744
N#Cc1cc2c	1960	0	5.707744
CC(C)Cc1cc	2000	0	5.69897
O=C1OCc2	810	1	6.091515
O=C(N[C@	1900	0	5.721246
O=C1CCN(	730	1	6.136677
C/C(=C\CC/	50	1	7.30103
COc1cc(CC	420	1	6.376751
C=CCc1ccc(	26000	0	4.585027
COc1ccc(C	1300	0	5.886057
Fc1ccc(-c2[	550	1	6.259637
CC(/C=C/C(	40	1	7.39794

COC1(c2cc	70	1	7.154902
CCCCCCc1c	2600	0	5.585027
NC(=O)N(C	440	1	6.356547
O=C(/N=c1	380	1	6.420216
Cc1coc(C)c	3500	0	5.455932
Cc1ccc(-c2	380	1	6.420216
CC1Cc2c(O	30	1	7.522879
O=C(O)/C=	290	1	6.537602
Cc1ccc2nc(	1800	0	5.744727
CCCCCCCC	870	1	6.060481
CCCCCCCC	140	1	6.853872
COC1(c2cc	1700	0	5.769551
CC(c1ccc(C	100	1	7
c1ccc(SCc2	70000	0	4.154902
COc1ccc2c	59300	0	4.226945
COc1ccc(C	97	1	7.013228
C=C(C/C=C	24500	0	4.610834
CCCC/C(=C	200	1	6.69897
Cc1ccc(-c2	60	1	7.221849
O=C1CCCN	79000	0	4.102373
CC1Cc2c(O	22	1	7.657577
Cc1ccc(C2=	120	1	6.920819
O=C(/N=c1	380	1	6.420216
COc1cc(OC	20	1	7.69897
CC1Cc2c(O	10	1	8
CC(C#Cc1c	1000	0	6
CC(C)CC(C#	600	1	6.221849
O=C(O)/C=	18	1	7.744727
COC1(c2cc	2500	0	5.60206
CCOC(=O)c	860	1	6.065502
CCCC/C(=C	750	1	6.124939
CC(C)(C)c1	8000	0	5.09691
Cc1ccc(-c2	740	1	6.130768
CC(C)CC(=C	1200	0	5.920819
CSc1cccc(-	930	1	6.031517
CCCCCCC(S	800	1	6.09691
CC1CN(c2c	6000	0	5.221849
NC(=O)N(C	90	1	7.045757
CC(c1ccc(-	350	1	6.455932
COc1ccc2c	237000	0	3.625252
O=C(Nc1cc	10760	0	4.968188
C/C(=N\OC	20	1	7.69897
CC(C)=CCc	2	1	8.69897
Cc1ccc(Cc2	4400	0	5.356547
O=C(NCCN	82000	0	4.086186
CC(C)N(O)C	70	1	7.154902
Clc1ccc2nc	1200	0	5.920819
CCOc1ccc(-	20000	0	4.69897
O=[N+]([O-	5000	0	5.30103
COC(=O)c1	6000	0	5.221849
COc1ccc(N	30000	0	4.522879

COC1=C(O(	10000	0	5
CC1(C)Cc2c	250	1	6.60206
CCCC[C@@	1100	0	5.958607
N#Cc1cc(-c	30	1	7.522879
COc1cc(/C=	300	1	6.522879
CNC(=O)c1	180	1	6.744727
O=C1OCc2	100	1	7
CCCC/C(=C	200	1	6.69897
O=C(Nc1cc	1010	0	5.995679
CCC(OCCc1	20	1	7.69897
COc1ccccc1	422	1	6.374688
N#Cc1cc(-c	27	1	7.568636
COc1ccc(-c	6000	0	5.221849
COc1ccc2[r	240	1	6.619789
CCOC(=O)c	45	1	7.346787
CCC(O)(CC)	0.9	1	9.045757
CCC(OC)(c1	6300	0	5.200659
CCOC(=O)C	1.7	1	8.769551
CCCCCCOc1	88	1	7.055517
CC[C@@](	330	1	6.481486
CC(=O)N(C)	1180	0	5.928118
O=C(NCCC1	9800	0	5.008774
CCCc1cccc(	6500	0	5.187087
CCC(O)(c1c	22000	0	4.657577
Cn1c2ccccc	100	1	7
COc1cc(C(C	80000	0	4.09691
COC(=O)c1	70	1	7.154902
CCCCCCC(S	600	1	6.221849
COc1ccc(Cr	10	1	8
N=C1/C(=N	2310	0	5.636388
CCCCC(Sc1	1500	0	5.823909
COc1ccc(N1	156000	0	3.806875
COc1ccc(/C	650	1	6.187087
CC(c1csc2c	3200	0	5.49485
CCCOc1cc(	3000	0	5.522879
COc1ccc(-n	770	1	6.113509
CSc1ccc(CC	62000	0	4.207608
NC(=O)N(C	185	1	6.732828
Cc1ccc(C2=	280	1	6.552842
CC1=NN(C(	57	1	7.244125
COc1cc(/C=	13900	0	4.856985
O=C(NC1C(	100000	0	4
COc1ccc(C(	0.31	1	9.508638
COc1cc2cc	100000	0	4
O=C(C(=O)I	0.99	1	9.004365
CCCc1cc(F)	7	1	8.154902
CC[C@H](C	120	1	6.920819
Cn1nnc(-c2	120	1	6.920819
Cc1cncc(Cr	15	1	7.823909
CC(C)(C)C#	900	1	6.045757
CCC(Sc1nc(	2000	0	5.69897

COc1cc(/C=	2	1	8.69897
Cc1ccc(C2=	1070	0	5.970616
COC1(c2cc	200	1	6.69897
CCCCC(Cc1	2100	0	5.677781
COc1ccc2c	66400	0	4.177832
Cc1ccc(C2=	330	1	6.481486
O=C(O)/C((	35	1	7.455932
CCCCCc1cc	3000	0	5.522879
CC1=NN(C(	5	1	8.30103
CC1=NN(C(	3	1	8.522879
COC1=CC=(	8	1	8.09691
CC1=NS(=C	10	1	8
CC1=C(C(=(	4	1	8.39794
CS(=O)(=O)C1=CC=C(C=		1	
CC1=C(C(=CC=C1)NC2=		1	
