# Supplementary material for: An Explainable 2D-QSAR Machine Learning Approach for Predicting COX-2 Inhibitory Activity Using Molecular Fingerprints
Source: Pharmaceuticals (Basel). 2026 Apr 29;19(5):698. doi: 10.3390/ph19050698 (PMC13209868; doi:10.3390/ph19050698)
Supplement: Supplementary file 1 [file pharmaceuticals-19-00698-s001.zip › Table S2 external Compounds.pdf]

SMILES	IC50	Binary_Clas	pIC50
Oc1ccc(-c2	8	1	8.09691
C/C(=N\OC	10	1	8
CC(C)c1ccc	10	1	8
CC(=O)N(O	13	1	7.886057
Oc1ccc(-c2	20	1	7.69897
Cc1cc(-c2cc	22	1	7.657577
O=C(O)/C=	22	1	7.657577
CC1Cc2c(O	23	1	7.638272
C/C(=N\OC	29	1	7.537602
CC/C(=N\O	30	1	7.522879
CCOc1ccc(-	30	1	7.522879
COC1(c2cc	40	1	7.39794
C/C(=C\CC/	40	1	7.39794
Cc1nsc(NC(	44	1	7.356547
C/C(=C\CC/	50	1	7.30103
Oc1ccc(-c2	50	1	7.30103
O=[N+]([O-	50	1	7.30103
CCCCC(O/N	52	1	7.283997
O=C(Nc1nc	53	1	7.275724
C/C(=C\CC/	57	1	7.244125
CC1Cc2c(O	60	1	7.221849
CC(C)Cc1cc	60	1	7.221849
O=C1N=C(c	60	1	7.221849
CCCCCCCC(	62	1	7.207608
COC(=O)c1	65	1	7.187087
COc1cc(/C=	70	1	7.154902
C/C(=C\CC/	70	1	7.154902
COc1ccc2cc	70	1	7.154902
CCOC(=O)C	70	1	7.154902
O=C(Nc1cc	71	1	7.148742
O=C(Nc1cc	74	1	7.130768
COc1ccccc1	76	1	7.119186
O=C1N=C(c	80	1	7.09691
O=C1N=C(c	80	1	7.09691
CCCOc1ccc	80	1	7.09691
Cc1c(C)c2c	80	1	7.09691
COc1ccc(/C	80	1	7.09691
COc1ccc(/C	80	1	7.09691
NC(=O)N(O	85	1	7.070581
CC(=O)N(O	86	1	7.065502
COc1ccc(C2	90	1	7.045757
COc1ccc(C2	90	1	7.045757
C/C(=C\CC/	90	1	7.045757
O=C1N=C(c	90	1	7.045757
C/C(=C\CC/	90	1	7.045757
O=C1N=C(c	90	1	7.045757
COc1ccc(/C	90	1	7.045757
CNC(=O)N(	98	1	7.008774
CCc1cc(/C=	100	1	7
COc1cc(/C=	100	1	7

CC(=O)C1(c	100	1	7
NC(=O)N(C	100	1	7
COc1ccc(/C	100	1	7
CCC(OC)(c1	100	1	7
C/C(=C\CC/	110	1	6.958607
C/C(=C\CC/	110	1	6.958607
COc1ccc(-n	110	1	6.958607
COc1cccc(C	115	1	6.939302
COc1ccccc1	119	1	6.924453
C/C(=C\CC/	120	1	6.920819
NC(=O)N(C	120	1	6.920819
CCOC(=O)c	120	1	6.920819
CCCCCCOc1	126	1	6.899629
C/C(=C\CC/	129	1	6.88941
CC(=O)c1cc	130	1	6.886057
COc1ccc(/C	130	1	6.886057
NC(=O)N(C	130	1	6.886057
O=C1N=C(c	130	1	6.886057
COc1ccc(/C	140	1	6.853872
COc1ccc(/C	140	1	6.853872
Cc1nc(NC(=	140	1	6.853872
O=C(NCc1c	148	1	6.829738
C/C(=C\CC/	150	1	6.823909
C/C(=C\CC/	150	1	6.823909
COc1ccc(/C	150	1	6.823909
CC(=O)Oc1	160	1	6.79588
CC(C)(Cc1c	160	1	6.79588
CC(/C=C/c1	160	1	6.79588
CC(=O)c1cc	160	1	6.79588
C/C(=C\CC/	170	1	6.769551
C/C(=C\CC[	170	1	6.769551
COc1ccc(/C	170	1	6.769551
NC(=O)N(C	170	1	6.769551
CCOC(=O)c	170	1	6.769551
C/C(=C\CC/	170	1	6.769551
C/C(=C\CC[	170	1	6.769551
COC(=O)C((	170	1	6.769551
C/C(=C\CC/	170	1	6.769551
CC1(C)C2C(	171	1	6.767004
CC1(C)Cc2c	180	1	6.744727
COc1ccc(/C	180	1	6.744727
C#CCCCCC(	180	1	6.744727
C/C(=C/CC/	180	1	6.744727
CCCCCCCC(	190	1	6.721246
CCOC(=O)C	200	1	6.69897
CC(C)c1cc(/	200	1	6.69897
COc1ccc(/C	200	1	6.69897
COC1(c2cc(	200	1	6.69897
CC(=O)c1cc	210	1	6.677781
COc1ccc(C2	210	1	6.677781
C/C(=C\CC/	210	1	6.677781

COc1ccc(C2	210	1	6.677781
CCCOc1ccc	210	1	6.677781
COc1cccc(C	210	1	6.677781
CCOC(=O)c	220	1	6.657577
CC1Oc2ccc	220	1	6.657577
Cc1nccn1-c	230	1	6.638272
CCOC(=O)c	230	1	6.638272
NC(=O)N(C	230	1	6.638272
CCCCCCCCC	230	1	6.638272
O=C1OCc2c	240	1	6.619789
Cc1nc(NC(=	240	1	6.619789
COc1ccccc1	250	1	6.60206
CCOC(=O)c	257	1	6.590067
COc1cc(CC1	260	1	6.585027
COc1ccc(/C	260	1	6.585027
COC1=CC(=	280	1	6.552842
CCOC(=O)c	290	1	6.537602
COC(=O)c1	300	1	6.522879
C/C(=C\CC/	300	1	6.522879
Fc1ccc(-c2[	300	1	6.522879
CCOC(=O)c	300	1	6.522879
CCCC/C(=C	300	1	6.522879
O=C1C=C(C	310	1	6.508638
C/C(=C\CC/	310	1	6.508638
CC1(C)Oc2c	310	1	6.508638
COc1ccc(/C	320	1	6.49485
O=C(/C=C/c	330	1	6.481486
CCCCCCCCC	340	1	6.468521
Cc1cc(OCC	351	1	6.454693
C/C(=C\CC[	380	1	6.420216
NC(=O)N(C	380	1	6.420216
CCCCCCCCC	380	1	6.420216
NC(=O)N(C	390	1	6.408935
CCCC/C(=C	400	1	6.39794
O=C(Nc1cc	400	1	6.39794
CCCCCCC(S	400	1	6.39794
O=C(O)c1cc	400	1	6.39794
C/C(=C\CC/	400	1	6.39794
Oc1cccc(-c1	400	1	6.39794
CC(=O)c1c(	420	1	6.376751
NC(=O)N(C	430	1	6.366532
C/C(=C\CC[	440	1	6.356547
C/C(=C\CC/	440	1	6.356547
C#CCCCCCC	470	1	6.327902
C/C(=C\CC[	470	1	6.327902
CCOC(=O)c	470	1	6.327902
CS(=O)(=O)	500	1	6.30103
O=C1Nc2cc	500	1	6.30103
O=C(O)C(Sc	500	1	6.30103
CCCCC(Sc1	500	1	6.30103
CCCCCCC(S	500	1	6.30103

CC1=NN(c2	520	1	6.283997
O=C(/C=C/	530	1	6.275724
Cc1ccc(/C=	540	1	6.267606
COc1ccc(C(	560	1	6.251812
O=C(Nc1cc	570	1	6.244125
COc1ccc(C2	570	1	6.244125
O=C1C=C(C	580	1	6.236572
CCCCC(Oc1	600	1	6.221849
CC1C(=O)O	600	1	6.221849
CCCCCCC(S	600	1	6.221849
CCCCC(Sc1	600	1	6.221849
CCCCCCC(S	600	1	6.221849
O=C(O)c1c	600	1	6.221849
CC(=O)Oc1	600	1	6.221849
Cc1ccc(S(=	600	1	6.221849
COc1cc(/C=	600	1	6.221849
c1cn2c(NC	610	1	6.21467
CCCCCCCC(	620	1	6.207608
O=C(O/N=(	620	1	6.207608
CS(=O)(=O)	620	1	6.207608
COc1ccc(/C	630	1	6.200659
CC(=O)c1cc	630	1	6.200659
Cc1nc(NC(=	640	1	6.19382
CCOC(=O)c	657	1	6.182435
CC(C)CC(=C	660	1	6.180456
O=C(/C=C/	660	1	6.180456
CC(C)(C)c1	690	1	6.161151
CC(C)(C)c1	700	1	6.154902
CCCC/C(=C	700	1	6.154902
COc1cc(/C=	700	1	6.154902
CCCCCCC(S	700	1	6.154902
CCOC(=O)c	700	1	6.154902
Oc1n[nH]c	700	1	6.154902
Cc1ccc(C2=	730	1	6.136677
COC1(c2cc	740	1	6.130768
NC1=NN(c2	750	1	6.124939
CCC(=O)Nc	760	1	6.119186
CCOC(=O)c	780	1	6.107905
O=C(/C=C/	790	1	6.102373
O=C1N=C(	790	1	6.102373
CCCC/C(=C	800	1	6.09691
CCOC(=O)c	800	1	6.09691
O=C(O)c1c	800	1	6.09691
O=C1C=C(c	800	1	6.09691
COc1cc(C)c	800	1	6.09691
CCCOc1cc(	800	1	6.09691
CCCC/C(=C	800	1	6.09691
CCOC(=O)c	800	1	6.09691
CCCOc1cc(	800	1	6.09691
CCCCC(Sc1	800	1	6.09691
CCCCCC[C@	800	1	6.09691

O=C(O)/C=	800	1	6.09691
CCOC(=O)c	800	1	6.09691
C/C(=C\CC	810	1	6.091515
CC(=O)N(C	840	1	6.075721
COc1ccc(S	850	1	6.070581
O=C(/C=C/	890	1	6.05061
Oc1ccc(-c2	900	1	6.045757
CCCCC1CC	900	1	6.045757
COc1ccc(C	900	1	6.045757
COc1cc(C)c	900	1	6.045757
COc1cc(C(=	900	1	6.045757
CCOC(=O)c	900	1	6.045757
CCCCC(Cc1	900	1	6.045757
CCCOc1cc(	900	1	6.045757
CCCC/C(=C	900	1	6.045757
CCCC/C(=C	900	1	6.045757
COc1ccc(C	900	1	6.045757
C/C(=C\CC	920	1	6.036212
C#CCCCCO	920	1	6.036212
CC(C)C(=O)	980	1	6.008774
C/C(=C\CC/	998	1	6.000869
CCc1cc(/C=	1000	1	6
O=C(NCC1	1000	1	6
Cc1cc(CCc2	1000	1	6
CC(C)c1cc(/	1000	1	6
COC(=O)CC	1000	1	6
CCCC/C(=C	1000	1	6
Cc1ccc(/C=	1000	1	6
Cc1ccc(CN(	1000	1	6
COc1ccc(C	1000	1	6
C/C(=C\CC/	1000	1	6
Cc1cc(O)cc	1000	1	6
NC(=O)C1(	1000	1	6
Cc1cc(Cc2c	1000	1	6
Cc1cc(/C=C	1000	1	6
CCc1cc(/C=	1000	1	6
Cc1cc(/C=C	1000	1	6
Cc1cc(O)cc	1000	1	6
Cc1ccc(/C=	1000	1	6
O=C(Nc1cc	1000	1	6
Cc1cc(/C=C	1000	1	6
Cc1cc(/C=C	1000	1	6
Cc1cc(CCC(	1000	1	6
Cc1nccn1-c	1000	1	6
COC(=O)/C	1000	1	6
C/C(=C\CC/	1000	1	6
Cc1cc(OCC	1000	1	6
COc1cc(/C=	1000	1	6
Cc1cc(/C=C	1000	1	6
COC(=O)C(	1000	1	6
CCCCCCCC	1000	1	6

CCCCCc1cc	1090	0	5.962574
Oc1ccc(CC(	1100	0	5.958607
C=CCc1ccc(	1100	0	5.958607
CCCCC(Sc1	1100	0	5.958607
O=C(O)c1cc	1100	0	5.958607
CCCCNC(=C	1200	0	5.920819
CC(C)(C)c1c	1200	0	5.920819
CCOC(=O)C	1200	0	5.920819
CCOC(=O)c	1200	0	5.920819
CCC(OC)(c1	1300	0	5.886057
CCCCCC(=C	1300	0	5.886057
O=C(O)/C=	1300	0	5.886057
COc1cccc(C	1300	0	5.886057
CC(=O)Oc1	1300	0	5.886057
Cc1ccc(Sc2	1400	0	5.853872
CCCc1ccc(C	1400	0	5.853872
CCCCCCC(S	1400	0	5.853872
CCCCCCC1=	1400	0	5.853872
Cc1ccc2nc(	1467	0	5.83357
CC(C)(C)CN	1480	0	5.829738
CCCCCC[C@	1500	0	5.823909
C=CCc1ccc(	1500	0	5.823909
CCCCCCN(c	1500	0	5.823909
CCCCCCC(S	1500	0	5.823909
Cc1ccc2nc(	1540	0	5.812479
CC(C)(C)c1c	1600	0	5.79588
CC1CN(c2c	1600	0	5.79588
CCCCCCCC(	1600	0	5.79588
CCCC/C(=C	1600	0	5.79588
CCCCCC(=C	1600	0	5.79588
CC(C)(C)c1c	1700	0	5.769551
CCCCCCC(S	1700	0	5.769551
Cc1ccccc1C	1700	0	5.769551
O=C1Nc2c(	1700	0	5.769551
CCOC(=O)c	1700	0	5.769551
O=C(O)c1cc	1700	0	5.769551
CC(=O)c1c(	1700	0	5.769551
CCCc1ccc(C	1700	0	5.769551
Cc1ccc(-c2c	1720	0	5.764472
CCOC(=O)C	1800	0	5.744727
CCOC(=O)c	1800	0	5.744727
O=C(O)C(Sc	1800	0	5.744727
C#CCCOC(=	1800	0	5.744727
c1cn2c(NC	1800	0	5.744727
O=C(O)/C=	1800	0	5.744727
CC(C)=CCC/	1800	0	5.744727
COc1ccc(/C	1860	0	5.730487
O=C(O)c1cc	1900	0	5.721246
Cc1ccc(-c2c	2000	0	5.69897
CC1C(=O)O	2000	0	5.69897
O=C(O)/C=	2000	0	5.69897

CCCCC/C=C	2000	0	5.69897
CCCCCCC(S	2000	0	5.69897
CCCCC(Cc1	2000	0	5.69897
C/C=C(/C=C	2000	0	5.69897
Oc1c(F)c(F)	2000	0	5.69897
COc1c(CC=	2000	0	5.69897
C=CCc1ccc(	2100	0	5.677781
O=C(O)c1cc	2100	0	5.677781
Oc1ccc(-c2	2100	0	5.677781
O=C(/C=C/c	2100	0	5.677781
O=C(O)/C=	2100	0	5.677781
CCCOc1ccc	2140	0	5.669586
CCCCC1=C(	2200	0	5.657577
O=C(O)/C=	2200	0	5.657577
C=CCc1ccc(	2200	0	5.657577
CCOC(=O)c	2300	0	5.638272
O=C(c1cccc	2300	0	5.638272
CCOC(=O)c	2300	0	5.638272
O=C(/C=C/c	2300	0	5.638272
CCOC(=O)c	2300	0	5.638272
Oc1ccc(Cc2	2300	0	5.638272
COc1c(CC=	2300	0	5.638272
CCCCNc1cc	2400	0	5.619789
CC(=O)c1c(	2400	0	5.619789
Cc1ccc(S(=	2400	0	5.619789
CCCCC(Sc1	2400	0	5.619789
CCOC(=O)C	2400	0	5.619789
COc1ccc(-c	2400	0	5.619789
CCOC(=O)c	2400	0	5.619789
CC(C)(C)c1c	2500	0	5.60206
O=C(O)/C=	2500	0	5.60206
O=C(O)/C=	2500	0	5.60206
C=C(C)[C@	2500	0	5.60206
O=C(O)C(Sc	2500	0	5.60206
CC(C)CCNc	2500	0	5.60206
CC(C)=CC/	2500	0	5.60206
CC(c1cc2c(	2500	0	5.60206
CCOC(=O)C	2600	0	5.585027
O=C1Nc2cc	2600	0	5.585027
CC(C)(C)c1c	2600	0	5.585027
CC1(C)Cc2c	2600	0	5.585027
CC(=O)Oc1	2600	0	5.585027
O=C(O)c1cc	2600	0	5.585027
CC(C)(C)c1c	2700	0	5.568636
COc1cc(/C=	2700	0	5.568636
CCCCCC(=C	2700	0	5.568636
Cc1cc(Oc2c	2700	0	5.568636
CC(=O)O[C	2700	0	5.568636
Cc1ccc(-c2c	2700	0	5.568636
CC(Nc1[nH	2800	0	5.552842
Cc1ccc(-c2c	2800	0	5.552842

CC(C)C(C1=	2800	0	5.552842
Cc1ccc(-n2	2800	0	5.552842
CN(C)c1ccc	2800	0	5.552842
Oc1ccc(-c2	2800	0	5.552842
CC(C)(C)c1c	2800	0	5.552842
CCCCCCc1c	2800	0	5.552842
O=C(O)/C=	2800	0	5.552842
CC(C)(C)c1c	2900	0	5.537602
CCOC(=O)c	2900	0	5.537602
Oc1nc(-c2c	2900	0	5.537602
Cc1ccc(-c2c	2900	0	5.537602
CC(C)(C)c1c	2900	0	5.537602
COc1cc(/C=	3000	0	5.522879
CCCOc1cc(	3000	0	5.522879
CCCCC/C=C	3000	0	5.522879
CCCCCCCC(	3000	0	5.522879
CC(C)(C)c1c	3000	0	5.522879
CC1C(=O)O	3000	0	5.522879
C[C@H]1[C	3000	0	5.522879
O=C(O)C(Sc	3100	0	5.508638
CCOC(=O)c	3100	0	5.508638
O=C(c1cccc	3100	0	5.508638
O=C1Nc2c(	3100	0	5.508638
O=C(O)c1cc	3200	0	5.49485
O=C(O)c1cc	3200	0	5.49485
COc1cc(CC(	3200	0	5.49485
CCOC(=O)c	3200	0	5.49485
CCOC(=O)C	3200	0	5.49485
COc1ccc(-n	3300	0	5.481486
O=C1Nc2c(	3300	0	5.481486
O=C(O)c1cc	3300	0	5.481486
COc1ccc(C(	3300	0	5.481486
COc1ccc(/C	3340	0	5.476254
CS(=O)(=O)	3400	0	5.468521
COC(=O)CN	3500	0	5.455932
Cc1ccc(S(=(	3500	0	5.455932
Cc1ccc(-c2c	3500	0	5.455932
COc1ccc(C2	3550	0	5.449772
CC(=O)c1c(	3600	0	5.443697
CCOCc1ccc	3700	0	5.431798
CCOC(=O)c	3800	0	5.420216
O=C(NC(=S	3800	0	5.420216
O=C(O)CSc	3900	0	5.408935
COc1cc(C)c	4000	0	5.39794
CCCCCCC1=	4000	0	5.39794
CCOC(=O)C	4000	0	5.39794
CCCCN(c1c	4000	0	5.39794
COc1ccccc1	4000	0	5.39794
COC1=CC(=	4100	0	5.387216
Cc1ccsc1C(	4100	0	5.387216
O=C(O)/C=	4200	0	5.376751

CCOC(=O)c	4200	0	5.376751
O=C1NCc2	4200	0	5.376751
C=CCc1ccc	4200	0	5.376751
O=C(O)c1c	4400	0	5.356547
O=C(O)C(S	4400	0	5.356547
CCCCCCCC	4500	0	5.346787
COc1c(CC=	4500	0	5.346787
C=CCc1ccc	4500	0	5.346787
O=C(O)/C=	4500	0	5.346787
O=C1C=C(c	4600	0	5.337242
COc1ccc(/	4660	0	5.331614
CCCCC(Cc1	4700	0	5.327902
Cc1ccc(NS(	4700	0	5.327902
CC(C)(C)c1	4800	0	5.318759
CCCCC(Sc1	4800	0	5.318759
Cc1ccc(S(=	4800	0	5.318759
CCCCC(Sc1	4800	0	5.318759
CCCCC(Sc1	4800	0	5.318759
Cc1ccc(/C=	4860	0	5.313364
Oc1ccc(/C=	4900	0	5.309804
CCOC(=O)c	5000	0	5.30103
CCOC(=O)c	5000	0	5.30103
C=C(C)C1C	5000	0	5.30103
Cc1cccc(N	5000	0	5.30103
O=C(O)c1c	5000	0	5.30103
O=C(O)/C=	5100	0	5.29243
CCCCC(Sc1	5200	0	5.283997
Nc1ccc(-c2	5300	0	5.275724
COc1c(CC=	5300	0	5.275724
COc1c(CC=	5300	0	5.275724
COc1cc(CC	5400	0	5.267606
COC(=O)[C	5460	0	5.262807
CC(C)=CCC	5900	0	5.229148
CCOC(=O)c	5900	0	5.229148
CCOCCn1c	5920	0	5.227678
OC1(c2ccc	6000	0	5.221849
O=Cc1ccc(-	6000	0	5.221849
COc1ccc(C(	6000	0	5.221849
COc1c(CC=	6000	0	5.221849
O=C(O)/C=	6000	0	5.221849
CCCCC(Cc1	6000	0	5.221849
CC1(C)Cc2	6100	0	5.21467
CN(C)c1ccc	6100	0	5.21467
Oc1ccc(-c2	6300	0	5.200659
O=C(O)c1c	6700	0	5.173925
O=C(O)c1c	6800	0	5.167491
O=C(Cc1cc	6800	0	5.167491
COc1ccc2c	7000	0	5.154902
C=CCN(CC=	7100	0	5.148742
CC(=O)Oc1	7600	0	5.119186
O=C(O)CSc	7600	0	5.119186

CC(C)(C)c1c	8000	0	5.09691
CC1C(=O)O	8000	0	5.09691
COc1ccc(-n	8000	0	5.09691
Cc1ccc(-c2c	8000	0	5.09691
CCCC(Sc1n	8000	0	5.09691
COc1cc(C)c	8000	0	5.09691
CC(=O)c1c(	8100	0	5.091515
CC(C)C(=O)	8100	0	5.091515
O=Nc1c(-c2	8200	0	5.086186
C#CCCNC(=	8230	0	5.0846
COC(=O)CC	8330	0	5.079355
O=C(O)c1c	8400	0	5.075721
O=C(O)/C=	8500	0	5.070581
CC(C)C(C1=	8600	0	5.065502
FC(F)(F)c1c	8800	0	5.055517
CCCCCc1cc	8900	0	5.05061
O=C1C=C(C	9000	0	5.045757
O=C(O)/C=	9200	0	5.036212
OC[C@@H	9200	0	5.036212
CC1(C)CC(=	9200	0	5.036212
O=C(OCc1c	9300	0	5.031517
CC(C)=CCC	9300	0	5.031517
COc1cc(O)c	9300	0	5.031517
Oc1ccc(Cc2	9400	0	5.026872
CCCCC(Sc1	9500	0	5.022276
COC1=CC(=	10000	0	5
O=C(O)c1c	10000	0	5
O=C(O)c1c	10000	0	5
O=[N+]([O-	10000	0	5
O=C(/C=C/c	10000	0	5
CS(=O)(=O)	10000	0	5
O=C(O)c1c	10000	0	5
O=C1C=C(C	10000	0	5
CC(C)=CCc1	10000	0	5
COc1cc2c(c	10000	0	5
CCOC(=O)c	10000	0	5
CCCCC(Sc1	10000	0	5
CCCCC(Sc1	10000	0	5
CS(=O)(=O)	10000	0	5
CCOC(=O)c	10000	0	5
O=C1C=C(C	10000	0	5
Cc1cccc(OC	10000	0	5
Cc1cccc(OC	10000	0	5
Cc1ccc(-c2c	10000	0	5
CC(C)=CCC	10000	0	5
COC1=CC(=	10000	0	5
CC(C)C(C1=	10000	0	5
COCCOc1c	10000	0	5
CCCCC(Sc1	10000	0	5
CCCCC1=C(	10000	0	5
C#CCCOC(=	10000	0	5

Oc1ccc(-c2	10000	0	5
Nc1ccc(-c2	10000	0	5
O=C(O)c1c	10000	0	5
CCCCC1=C(	10000	0	5
CCCCC(Sc1	10000	0	5
CCCCCCC1	10000	0	5
CC(=O)c1c(	10000	0	5
CCCCC(Sc1	10000	0	5
COc1cc(-c2	10000	0	5
CC(C)C(=O)	10000	0	5
COc1cccc(	10000	0	5
COc1cccc(	10000	0	5
O=C(O)c1c	10000	0	5
CCCCCc1cc	10000	0	5
COc1ccc(-c	10000	0	5
CCCCC(Cc1	10000	0	5
CC(=O)Oc1	10000	0	5
Cc1nnsc1C	10000	0	5
CC(=O)Oc1	10000	0	5
CCOC(=O)c	10000	0	5
O=C1COCC	10000	0	5
COc1cc(F)c	10000	0	5
COc1ccc(-n	10000	0	5
CCOC(=O)C	10000	0	5
Cc1occc(=S	11000	0	4.958607
OCCc1ccc(	13000	0	4.886057
COc1cc(/C=	13900	0	4.856985
CC(C)=CCC	14000	0	4.853872
Cc1cccc(C2	14200	0	4.847712
C=CCc1ccc	15000	0	4.823909
O=C([O-])/	17000	0	4.769551
CC(C)Cc1cc	17000	0	4.769551
C/C=C/c1cc	18400	0	4.735182
COC1(c2cc	19000	0	4.721246
CCCCC/C=C	20000	0	4.69897
CCCCC/C=C	20000	0	4.69897
CN(C)c1ccc	20000	0	4.69897
COc1cc(-c2	20900	0	4.679854
NS(=O)(=O)	21310	0	4.671417
COc1cc(-c2	22800	0	4.642065
COc1ccc(N	23500	0	4.628932
Nc1ccccc1-	24300	0	4.614394
CC1(C)[C@	24800	0	4.605548
CC1(C)[C@	25700	0	4.590067
COc1ccc(-c	27200	0	4.565431
CCCCC/C=C	28000	0	4.552842
Cc1ccc(/C=	30000	0	4.522879
O=C1/C(=C	30000	0	4.522879
Oc1ccc(-c2	30000	0	4.522879
c1ccc(CCCr	30000	0	4.522879
COc1ccc(/C	30000	0	4.522879

O=C1N=C(N	30000	0	4.522879
Cc1ccccc1C	30000	0	4.522879
c1ccc(CCCr	30000	0	4.522879
c1ccc(CCCr	30000	0	4.522879
Cc1ccccc1C	30000	0	4.522879
CC1(C)CC[C	30400	0	4.517126
CC1(C)CC[C	30600	0	4.514279
CCCCCCC(S	31000	0	4.508638
CC1(C)[C@	31800	0	4.497573
O=c1/c(=C/	33800	0	4.471083
CCCCCCC(S	34000	0	4.468521
CC1(C)CC[C	40000	0	4.39794
CC1(C)CC[C	40000	0	4.39794
CC1(C)CC[C	40000	0	4.39794
C[C@H]1[C	40000	0	4.39794
C[C@H]1[C	40000	0	4.39794
C[C@H]1[C	40000	0	4.39794
CC1(C)CC[C	40000	0	4.39794
CC1(C)CC[C	40000	0	4.39794
CC(=O)O[C	40000	0	4.39794
CC1(C)CC[C	40000	0	4.39794
CC(=O)O[C	40000	0	4.39794
C[C@H]1[C	40000	0	4.39794
C[C@H]1[C	40000	0	4.39794
CC1(C)CC[C	40000	0	4.39794
COc1cc(O)c	41400	0	4.383
CC1(C)CC[C	42000	0	4.376751
CC1(C)CC[C	42000	0	4.376751
CC1(C)[C@	42000	0	4.376751
C[C@H]1[C	42000	0	4.376751
O=C(O)c1cc	50000	0	4.30103
CCCNC(=O)	50000	0	4.30103
O=C(/C=C/	54000	0	4.267606
CCCCC/C=C	65000	0	4.187087
COc1cc(Cl)	68000	0	4.167491
O=C(O)c1cc	75000	0	4.124939
COc1ccc2c	81400	0	4.089376
