# Supplementary material for: An Explainable 2D-QSAR Machine Learning Approach for Predicting COX-2 Inhibitory Activity Using Molecular Fingerprints
Source: Pharmaceuticals (Basel). 2026 Apr 29;19(5):698. doi: 10.3390/ph19050698 (PMC13209868; doi:10.3390/ph19050698)
Supplement: Supplementary file 1 [file pharmaceuticals-19-00698-s001.zip › Table S3 External Validation Dataset.pdf]

```
label;SMILES;Prediction;Probability (Active);AD_similarity;In_AD;AD_warning;MolWt
1;Oc1ccc(-c2cccc3c2SC2C=CC=CC2S3)cc1O;Active;0.83;0.353;No;Outside AD: prediction
1;C/C(=N\OCC#N)c1ccc(Sc2cc([C@@]3(C)COC(C)(C)O3)cs2)cc1;Active;0.807;0.696;Yes;;4
1;CC(C)c1ccc2c(c1)c(SC(C)(C)C)c(CC(C)(C)C(=O)O)n2Cc1ccc(Cl)cc1;Active;0.647;0.58;
1;CC(=O)N(O)C/C=C/c1cccc(Oc2ccccc2)c1;Active;0.917;0.493;Yes;;283.33;3.73;49.77;1
1;Oc1ccc(-c2cccc(Cl)c2)cc1O;Active;0.89;0.54;Yes;;220.66;3.42;40.46;2;2;1;0.723;0
1;Cc1cc(-c2ccc(C(=O)[C@@H]3CCCC[C@H]3C(=O)Nc3cnn4c3C(=O)N(C)CC4)cc2)n[nH]1;Active
1;O=C(O)/C=N/OC(c1ccc(OCc2nc3ccccc3s2)cc1Br)C1CCCCC1;Active;0.917;0.705;Yes;;503.
1;CC1Cc2c(OCc3ccc(-c4ccccc4)cn3)ccc3c2c(c(CCOc2ccccc2CC(=O)O)n3Cc2cccc(F)c2)S1;Ac
1;C/C(=N\OC(CC1CCCCC1)c1ccc(OCc2ccc3ccccc3n2)cc1)C(=O)O;Active;0.983;0.754;Yes;;4
1;CC/C(=N\OC(c1ccc(OCc2ccc3ccccc3n2)cc1)C1CCCCC1)C(=O)O;Active;1.0;0.813;Yes;;446
1;CCOc1ccc(-c2csc(Nc3ccc(O)cc3)n2)cc1;Active;0.828;0.652;Yes;;312.39;4.66;54.38;2
1;COC1(c2cc(F)cc(OCc3ccc4c(c3)sc(=O)n4C)c2)CCOCC1;Active;0.853;0.703;Yes;;403.48;
1;C/C(=C\CC/C(C)=C/CC[C@]1(C)CCc2c(C)c(O)c(C)c(C)c2O1)CCC=C(CO)CO;Active;0.927;0.
1;Cc1nsc(NC(=O)C2CCCCC2C(=O)c2ccc(-c3ccn[nH]3)cc2)c1C#N;Active;0.965;0.578;Yes;;4
1;C/C(=C\CC/C(C)=C/CC[C@]1(C)CCc2cc(O)c(C)c(C)c2O1)CCC=C(CO)CO;Active;0.9;0.474;Y
1;Oc1ccc(-c2nc(-c3ccc(F)cc3)c(-c3ccncc3)[nH]2)cc1;Active;0.895;0.714;Yes;;331.35;
1;O=[N+]([O-])c1ccc(-c2nc(-c3ccc(F)cc3)c(-c3ccncc3)[nH]2)cc1;Active;0.65;0.666;Ye
1;CCCCC(O/N=C/C(=O)O)c1ccc(OCc2ccc3ccccc3n2)cc1;Active;0.96;0.693;Yes;;392.46;5.1
1;O=C(Nc1nc(C2CC2)ns1)C1CCCCC1C(=O)c1ccc(-c2ccn[nH]2)cc1;Active;0.988;0.602;Yes;;
1;C/C(=C\CC/C(C)=C/CC[C@]1(C)CCc2cc(O)cc(C)c2O1)CC/C=C(\C)C(=O)O;Active;0.935;0.6
1;CC1Cc2c(OCc3ccc(-c4ccccc4)cn3)ccc3c2c(c(CCOc2ccccc2CC(=O)O)n3Cc2ccc(Cl)cc2)S1;A
1;CC(C)Cc1ccc(C(C)c2nc3cc(CCC(=O)O)ccc3n2Cc2ccccc2Cl)cc1;Active;0.527;0.447;Yes;;
1;O=C1N=C(c2ccc3ccccc3c2)S/C1=C\c1ccc(Oc2ccccc2)cc1;Active;0.843;0.565;Yes;;407.4
1;CCCCCCCCc1c(-c2ccc(O)c(O)c2)oc2c(OC)c(OC)cc(OC)c2c1=O;Active;1.0;1.0;Yes;;456.5
1;COC(=O)c1ccc2c(c1)Nc1ccccc1O2;Active;0.547;0.617;Yes;;241.25;3.32;47.56;1;4;1;0
1;COc1cc(/C=C/c2ccc(O)c(OC)c2)cc(OC)c1;Active;0.565;0.67;Yes;;286.33;3.59;47.92;1
1;C/C(=C\CC/C(C)=C/CC[C@]1(C)CCc2c(CN(C)C)c(O)cc(C)c2O1)CC/C=C(\C)C(=O)O;Active;0
1;COc1ccc2cc(/C=C3\SC(c4ccc(Cl)cc4)=NC3=O)ccc2c1;Active;0.93;0.699;Yes;;379.87;5.
1;CCOC(=O)C1(c2cccc(OCc3ccc4c(ccc(=O)n4C)c3)c2)CCOCC1;Active;0.668;0.707;Yes;;421
1;O=C(Nc1ccc2c(c1)CCN2Cc1ccc(F)cc1)NC12C[C@H]3C[C@@H](C1)C[C@@H](C2)C3;Active;0.7
1;O=C(Nc1ccc2c(c1)CCN2Cc1ccc(F)cc1)C12C[C@H]3C[C@@H](C1)C[C@@H](C2)C3;Active;0.59
1;COc1ccccc1CNC(=O)/C(C)=C/CC/C(C)=C/CC/C(C)=C/CC[C@]1(C)CCc2cc(O)cc(C)c2O1;Activ
1;O=C1N=C(c2ccc(Cl)cc2)S/C1=C\c1ccc(OCc2ccccc2)cc1;Active;0.86;0.645;Yes;;405.91;
1;O=C1N=C(c2ccc(Cl)cc2)S/C1=C\c1ccc(Oc2ccccc2)cc1;Active;0.895;0.686;Yes;;391.88;
1;CCCOc1ccc(/C=C2\SC(c3ccc(Cl)cc3)=NC2=O)cc1;Active;0.89;0.663;Yes;;357.86;5.19;3
1;Cc1c(C)c2c(c(C)c1O)CC[C@@](C)(CCCC(C)CCCC(C)CCCC(C)C(=O)O)O2;Active;0.772;0.398
1;COc1ccc(/C=C2\SC(c3ccc4ccccc4c3)=NC2=O)cc1;Active;0.897;0.707;Yes;;345.42;4.91;
1;COc1ccc(/C=C2\SC(c3ccc4ccccc4c3)=NC2=O)cc1O;Active;0.858;0.535;Yes;;361.42;4.62
1;NC(=O)N(O)C[C@H]1COc2ccc(Oc3ccc(F)cc3)cc2O1;Active;0.825;0.562;Yes;;334.3;2.53;
1;CC(=O)N(O)CC1=Cc2cc(Oc3ccccc3)ccc2OC1;Active;0.955;0.775;Yes;;311.34;3.49;59.0;
1;COc1ccc(C2=NC(=O)/C(=C/c3ccc(Cl)cc3)S2)cc1;Active;0.993;0.866;Yes;;329.81;4.41;
1;COc1ccc(C2OCC(=CCC/C(C)=C/CC/C(C)=C/CC[C@]3(C)CCc4c(C)c(O)c(CO)c(C)c4O3)CO2)cc1
1;C/C(=C\CC/C(C)=C/CC[C@]1(C)CCc2cc(OC(=O)CCC(=O)O)cc(C)c2O1)CCC=C(CO)CO;Active;0
1;O=C1N=C(c2ccc(Cl)cc2)S/C1=C\c1cccc(Oc2ccccc2)c1;Active;0.905;0.59;Yes;;391.88;6
1;C/C(=C\CC/C(C)=C/CC[C@]1(C)CCc2c(C)c(O)cc(C)c2O1)CCC=C(CO)CO;Active;0.915;0.615
1;O=C1N=C(c2ccc(Oc3ccccc3)cc2)S/C1=C\c1ccc(Oc2ccccc2)cc1;Active;0.91;0.674;Yes;;4
1;COc1ccc(/C=C2\SC(c3ccc(Br)cc3)=NC2=O)cc1;Active;0.945;0.775;Yes;;374.26;4.52;38
1;CNC(=O)N(O)CC1COc2ccc(Oc3ccccc3)cc2O1;Active;0.835;0.609;Yes;;330.34;2.65;80.26
1;CCc1cc(/C=C/c2cccs2)cc(CC)c1O;Active;0.73;0.641;Yes;;258.39;4.75;20.23;1;2;4;0.
1;COc1cc(/C=C/c2cccs2)cc(OC)c1O;Active;0.892;0.702;Yes;;262.33;3.64;38.69;1;4;4;0
1;CC(=O)C1(c2cccc(OCc3ccc4c(ccc(=O)n4C)c3)c2)CCOCC1;Active;0.641;0.748;Yes;;391.4
1;NC(=O)N(O)CC1COc2ccc(Oc3ccc(F)cc3)cc2O1;Active;0.825;0.562;Yes;;334.3;2.53;94.2
1;COc1ccc(/C=C2\SC(c3cccc(Cl)c3)=NC2=O)cc1;Active;0.925;0.739;Yes;;329.81;4.41;38
```

1;CCC(OC)(c1cccc(OCc2ccc3ccccc3c2)c1)c1nccs1;Inactive;0.128;0.767;Yes;;389.52;6.1
1;C/C(=C\CC/C(C)=C/CC[C@]1(C)CCc2c(CN3CCCCC3)c(O)cc(C)c2O1)CCC=C(CO)CO;Active;0.8
1;C/C(=C\CC/C(C)=C/CC[C@]1(C)CCc2c(CN(C)C)c(O)cc(C)c2O1)CCC=C(CO)CO;Active;0.878;
1;COc1ccc(-n2nc(C#CC(C)N(O)C(C)=O)cc2-c2ccc(Cl)cc2)cc1;Active;0.743;0.373;Yes;;40
1;COc1cccc(CNC(=O)/C(C)=C/CC/C(C)=C/CC/C(C)=C/CC[C@]2(C)CCc3cc(O)cc(C)c3O2)c1;Act
1;COc1ccccc1CNC(=O)/C(C)=C/CC/C(C)=C/CC/C(C)=C/CC[C@]1(C)CCc2c(C)c(O)cc(C)c2O1;Ac
1;C/C(=C\CC/C(C)=C/CC[C@]1(C)CCc2c(CN3CCOCC3)c(O)cc(C)c2O1)CCC=C(CO)CO;Active;0.8
1;NC(=O)N(O)CC1COc2ccc(Oc3ccccc3)cc2O1;Active;0.765;0.627;Yes;;316.31;2.39;94.25;
1;CCOC(=O)c1c(-c2cc(C)cc(C)c2)[nH]c2c1cc(O)c1ccccc12;Active;0.963;0.76;Yes;;359.4
1;CCCCCCOc1c(-c2ccc(O)c(O)c2)oc2cc(OC)c(OC)c(OC)c2c1=O;Active;0.82;0.831;Yes;;444
1;C/C(=C\CC/C(C)=C/CC[C@]1(C)CCc2cc(O)cc(C)c2O1)CC/C=C(\C)C(=O)N1CCCCC1;Active;0.
1;CC(=O)c1cc(-c2ccccc2)c2ccc(OCc3cccc(C4(O)CCOCC4)c3)cc2c1;Active;0.713;0.771;Yes
1;COc1ccc(/C=C2\SC(c3ccc(Cl)cc3)=NC2=O)c2ccccc12;Active;0.797;0.58;Yes;;379.87;5.
1;NC(=O)N(O)CC1=Cc2cc(OCCc3ccccc3)ccc2OC1;Active;0.81;0.677;Yes;;354.41;3.24;85.
1;O=C1N=C(c2ccc(Oc3ccccc3)cc2)S/C1=C\c1ccc2ccccc2c1;Active;0.858;0.564;Yes;;407.4
1;COc1ccc(/C=C2\SC(c3cccc(NC(=O)C45CC6CC(CC(C6)C4)C5)c3)=NC2=O)cc1;Active;0.833;0
1;COc1ccc(/C=C2\SC(c3ccc(Oc4ccccc4)cc3)=NC2=O)c2ccccc12;Active;0.775;0.567;Yes;;4
1;Cc1nc(NC(=O)c2ccccc2)sc1C(=O)/C=C/c1ccc(F)cc1;Active;0.685;0.512;Yes;;366.42;4.
1;O=C(NCc1cccc2ccccc12)Nc1ccc2c(c1)CCN2Cc1ccc(F)cc1;Active;0.755;0.609;Yes;;425.5
1;C/C(=C\CC/C(C)=C/CC[C@]1(C)CCc2c(C)c(O)cc(C)c2O1)CC/C=C(\C)C(=O)O;Active;0.95;0
1;C/C(=C\CC/C(C)=C/CC[C@]1(C)CCc2cc(O)cc(C)c2O1)CCC=C(CO)CO;Active;0.877;0.61;Yes
1;COc1ccc(/C=C2\SC(c3ccc(C(=O)c4ccccc4)cc3)=NC2=O)cc1;Active;0.887;0.741;Yes;;399
1;CC(=O)Oc1ccc(/C=C/C(=O)OCCc2cnnn2CCCOC(=O)C2(C)CCc3c(C)c(OC(C)=O)c(C)c(C)c3O2)c
1;CC(C)(Cc1c2c3c(c(OCc4ccc(-c5ccccc5)cn4)ccc3n1Cc1ccc(Cl)cc1)CCCS2)C(=O)O;Active;
1;CC(/C=C/c1ccc(Oc2ccc(F)cc2)o1)N(O)C(N)=O;Active;0.932;0.698;Yes;;306.29;3.38;88
1;CC(=O)c1ccc(/C=C2\SC(c3ccc(C)cc3)=NC2=O)cc1;Active;0.748;0.761;Yes;;321.4;4.26;
1;C/C(=C\CC/C(C)=C/CC[C@]1(C)CCc2c(C=O)c(O)cc(C)c2O1)CCC=C(CO)CO;Active;0.855;0.5
1;C/C(=C\CC[C@]1(C)CCc2cc(O)cc(C)c2O1)CC/C=C(\C)CCCC(C)C(=O)O;Active;0.833;0.597;
1;COc1ccc(/C=C2\SC(c3ccc4ccccc4c3)=NC2=O)c2ccccc12;Active;0.77;0.538;Yes;;395.48;
1;NC(=O)N(O)C[C@@H]1COc2ccc(Oc3ccc(F)cc3)cc2O1;Active;0.825;0.562;Yes;;334.3;2.53
1;CCOC(=O)c1c(CSc2c(C)cccc2C)[nH]c2c1cc(O)c1ccccc12;Active;0.973;0.781;Yes;;405.5
1;C/C(=C\CC/C(C)=C/CC[C@]1(C)CCc2c(CN3CCCC3)c(O)cc(C)c2O1)CCC=C(CO)CO;Active;0.80
1;C/C(=C\CC[C@]1(C)CCc2cc(O)cc(C)c2O1)CCC=C(CO)CO;Active;0.877;0.61;Yes;;360.49;4
1;COC(=O)C(C)CCC/C(C)=C/CC/C(C)=C/CC[C@]1(C)CCc2cc(O)cc(C)c2O1;Active;0.825;0.59;
1;C/C(=C\CC/C(C)=C/CC[C@]1(C)CCc2c(C)c(O)c(C=O)c(C)c2O1)CCC=C(CO)CO;Active;0.868;
1;CC1(C)C2CC[C@@]1(CS(=O)(=O)Nc1ccc3c(c1)CCN3Cc1ccc(F)cc1)C(=O)C2;Inactive;0.458;
1;CC1(C)Cc2c(-c3ccccc3)c(-c3ccc(Cl)cc3)c(CC(=O)O)n2C1;Inactive;0.49;0.679;Yes;;37
1;COc1ccc(/C=C2\SC(c3ccccc3Cl)=NC2=O)cc1;Active;0.833;0.671;Yes;;329.81;4.41;38.6
1;C#CCCCCCCCCOC(=O)/C=C/c1ccc(OC(C)=O)c(OC(C)=O)c1;Active;0.578;0.523;Yes;;400.47
1;C/C(=C/CC/C(C)=C/CC/C(C)=C/CC[C@]1(C)CCc2cc(O)c(C)c(C)c2O1)C(=O)O;Active;0.93;0
1;CCCCCCCCCCCCCCCCC1=C(O)C(=O)C=C(O)C1=O;Active;0.983;1.0;Yes;;364.53;6.26;74.6;2
1;CCOC(=O)C1(c2cc(F)cc(OCc3ccc(-n4ccnc4C)cc3)c2)CCOCC1;Active;0.635;0.813;Yes;;43
1;CC(C)c1cc(/C=C/c2cccnc2)cc(C(C)C)c1O;Active;0.685;0.399;Yes;;281.4;5.2;33.12;1;
1;COc1ccc(/C=C2\SC(c3ccc(F)cc3)=NC2=O)cc1;Active;0.95;0.775;Yes;;313.35;3.9;38.66
1;COC1(c2cc(F)cc(OCc3ccc4ncncc4c3)c2)CCOCC1;Active;0.928;0.667;Yes;;368.41;4.0;53
1;CC(=O)c1cc2c3c(c(C)cc2oc1=O)O[C@](C)(CC/C=C(\C)CC/C=C(\C)CCC=C(CO)CO)CC3;Active
1;COc1ccc(C2OCC(=CCC/C(C)=C/CC/C(C)=C/CC[C@]3(C)CCc4cc(O)cc(C)c4O3)CO2)cc1OC;Acti
1;C/C(=C\CC/C(C)=C/CC[C@]1(C)CCc2c(CO)c(O)cc(C)c2O1)CCC=C1COC(c2ccc([N+](=O)[O-])
1;COc1ccc(C2=NC(=O)/C(=C/c3ccc(C)cc3)S2)cc1;Active;0.943;0.832;Yes;;309.39;4.06;3
1;CCCOc1ccc(C2=NC(=O)/C(=C/c3ccc(OC)cc3)S2)cc1;Active;0.925;0.712;Yes;;353.44;4.5
1;COc1cccc(CCOC(=O)/C=C/c2cc(OC)c(O)c(OC)c2)c1;Active;0.891;0.76;Yes;;358.39;3.22
1;CCOC(=O)c1c(CSc2cc(Cl)cc(Cl)c2)[nH]c2c1cc(O)c1ccccc12;Active;0.953;0.745;Yes;;4
1;CC1Oc2ccc(OCc3ccccc3)cc2C=C1CN(O)C(N)=O;Active;0.773;0.58;Yes;;340.38;3.2;85.02
1;Cc1nccn1-c1ccc(Sc2cccc(C3(C(N)=O)CCOCC3)c2)cc1;Active;0.657;0.706;Yes;;393.51;3

1;CCOC(=O)c1c(CSc2c(C)cc(C)cc2C)n(C)c2ccc(O)cc12;Inactive;0.419;0.395;Yes;;383.51
1;NC(=O)N(O)CC1=Cc2cc(-c3ccccc3)ccc2OC1;Active;0.78;0.655;Yes;;296.33;2.9;75.79;2
1;CCCCCCCCCCCCCCCCC1=C(O)C(=O)C=C(O)C1=O;Active;0.983;1.0;Yes;;350.5;5.87;74.6;2;4
1;O=C1OCc2c1ccc1cc(OCc3cccc(C4(O)CC5COC(C4)O5)c3)ccc1c2-c1ccccc1;Active;0.958;0.78
1;Cc1nc(NC(=O)c2ccccc2)sc1C(=O)/C=C/c1cccc([N+](=O)[O-])c1;Active;0.618;0.484;Yes
1;COc1ccccc1CCOC(=O)/C=C/c1cc(OC)c(O)c(OC)c1;Active;0.926;0.77;Yes;;358.39;3.22;7
1;CCOC(=O)c1c(CSc2cccc(Cl)c2)[nH]c2c1cc(O)c1ccccc12;Active;0.963;0.738;Yes;;411.9
1;COc1cc(CC2C(=O)N(c3c(Cl)cccc3Cl)c3ccccc32)cc(OC)c1OC;Active;0.637;0.491;Yes;;45
1;COc1ccc(/C=C2\SC(c3ccc(NC(=O)CCc4ccccc4)cc3)=NC2=O)cc1;Active;0.79;0.603;Yes;;4
1;COC1=CC(=O)C(Oc2ccc3ccccc3c2)=CC1=O;Active;0.557;0.329;No;Outside AD: predictio
1;CCOC(=O)c1c(CSc2cc(C)cc(C)c2)[nH]c2c1cc(O)c1ccccc12;Active;0.963;0.747;Yes;;405
1;COC(=O)c1c(Nc2cccc(Cl)c2)[nH]c2ccc(O)cc12;Inactive;0.11;0.682;Yes;;316.74;4.06;
1;C/C(=C\CC/C(C)=C/CC[C@]1(C)CCc2cc(O)c(C)c(C)c2O1)CC/C=C(\C)C(=O)O;Active;0.93;0
1;Fc1ccc(-c2[nH]c(-c3ccccc3)nc2-c2ccncc2)cc1;Active;0.805;0.731;Yes;;315.35;4.94;
1;CCOC(=O)c1c(Nc2cccc(Cl)c2)[nH]c2ccc(O)cc12;Inactive;0.177;0.669;Yes;;330.77;4.4
1;CCCC/C(=C\c1cc(OCCc2ccc(C(F)(F)F)cc2)ccc1OCCc1ccc(C(F)(F)F)cc1)C(=O)O;Active;0.
1;O=C1C=C(Cc2ccc3ccccc3c2)C(=O)C=C1O;Inactive;0.443;0.311;No;Outside AD: predicti
1;C/C(=C\CC/C(C)=C/CC[C@]1(C)CCc2c(CN3CCN(C)CC3)c(O)cc(C)c2O1)CCC=C(CO)CO;Active;
1;CC1(C)Oc2ccc(Oc3ccccc3)cc2C=C1CN(O)C(N)=O;Active;0.94;0.602;Yes;;340.38;3.8;85.
1;COc1ccc(/C=C2\SC(c3ccc(OC)cc3)=NC2=O)cc1;Active;0.985;0.876;Yes;;325.39;3.76;47
1;O=C(/C=C/c1cc(O)ccc1O)OCCc1ccccc1;Active;0.59;0.529;Yes;;284.31;2.9;66.76;2;4;5
1;CCCCCCCCCCCCCc1c(-c2ccc(O)c(O)c2)oc2c(OC)c(OC)cc(O)c2c1=O;Active;1.0;1.0;Yes;;49
1;Cc1cc(OCCCCCN2CCN(C(c3ccccc3)c3ccccc3)CC2)c(C)c(C)c1O.Cl;Inactive;0.274;0.98;Ye
1;C/C(=C\CC[C@]1(C)CCc2cc(O)cc(C)c2O1)CCC(O)C(C)(O)CCC=C(CO)CO;Active;0.786;0.479
1;NC(=O)N(O)CC1=Cc2cc(OCc3ccccc3)ccc2OC1;Active;0.81;0.696;Yes;;326.35;2.81;85.02
1;CCCCCCCCC1=C(O)C(=O)C=C(O)C1=O;Active;0.983;1.0;Yes;;252.31;3.14;74.6;2;4;7;0.5
1;NC(=O)N(O)CC1COc2cc(Oc3ccccc3)ccc2O1;Active;0.765;0.63;Yes;;316.31;2.39;94.25;2
1;CCCC/C(=C\c1cc(OCCC2CCCCC2)ccc1OCc1ccc(C(F)(F)F)cc1)C(=O)O;Active;0.583;0.752;Y
1;O=C(Nc1ccc(-c2nc3ccccn3c2NC2CCCCC2)cc1)c1cccs1;Active;0.695;0.462;Yes;;416.55;6
1;CCCCCCC(Sc1nc(Cl)cc(Nc2ccc(-c3ccc(C#N)cc3)cc2)n1)C(=O)O;Active;0.868;0.632;Yes;
1;O=C(O)c1ccc(N(Cc2cccc(OCc3ccccc3)c2)C(=O)c2ccc(-c3ccccc3)cc2)cc1;Active;0.812;0
1;C/C(=C\CC/C(C)=C/CC[C@]1(C)CCc2c(C)c(O)c(C)c(C)c2O1)CC/C=C(\C)C(=O)O;Active;0.9
1;Oc1cccc(-c2ccc(-c3cn(CSc4ccccc4)nn3)cc2)c1;Inactive;0.495;0.299;No;Outside AD:
1;CC(=O)c1c(O)cc(O)c(C/C=C(\C)CCC=C(C)C)c1O;Inactive;0.43;0.459;Yes;;304.39;4.24;
1;NC(=O)N(O)CC1=Cc2cc(OCc3ccc(C(F)(F)F)cc3)ccc2OC1;Active;0.735;0.628;Yes;;394.35
1;C/C(=C\CC[C@]1(C)CCc2c(C)c(O)c(C)c(C)c2O1)CC/C=C(\C)CCC(=O)O;Active;0.915;0.639
1;C/C(=C\CC/C(C)=C/CC[C@]1(C)CCc2cc(O)cc(C)c2O1)CC/C=C/C(=O)O;Active;0.855;0.63;Y
1;C#CCCCCCCOC(=O)/C=C/c1ccc(OC(C)=O)c(OC(C)=O)c1;Active;0.578;0.523;Yes;;358.39;3.
1;C/C(=C\CC[C@]1(C)CCc2cc(O)cc(C)c2O1)CC/C=C(\C)CCC(=O)O;Active;0.877;0.657;Yes;;
1;CCOC(=O)c1cc2c3c(c(C)cc2oc1=O)O[C@](C)(CC/C=C(\C)CC/C=C(\C)CCC=C(CO)CO)CC3;Acti
1;CS(=O)(=O)NCc1ccc(-c2nc(-c3ccc(F)cc3)c(-c3ccncc3)[nH]2)cc1;Active;0.845;0.677;Y
1;O=C1Nc2cc(Br)ccc2/C1=C1/Nc2ccccc2/C1=N\O;Inactive;0.136;0.38;Yes;;356.18;3.42;7
1;O=C(O)C(Sc1nc(Cl)cc(Nc2ccc3ccccc3c2)n1)c1cccc2ccccc12;Active;0.97;0.82;Yes;;471
1;CCCCC(Sc1nc(OCCc2ccc(OC(F)(F)F)cc2)cc(OCCc2ccc(OC(F)(F)F)cc2)n1)C(=O)O;Active;0
1;CCCCCCC(Sc1nc(Cl)cc(Nc2cc(C)c(-c3ccccc3)cc2OC)n1)C(=O)O;Active;0.823;0.614;Yes;
1;CC1=NN(c2ccccc2C(=O)O)C(=O)/C1=N/Nc1ccc(S(N)(=O)=O)cc1;Active;0.583;0.572;Yes;;
1;O=C(/C=C/c1cc(O)ccc1O)CCc1ccccc1;Inactive;0.487;0.427;Yes;;268.31;3.31;57.53;2;
1;Cc1ccc(/C=C/C(=O)c2sc(NC(=O)c3ccc(F)cc3)nc2C)cc1;Active;0.715;0.498;Yes;;380.44
1;COc1ccc(C(=O)Nc2nc(C)c(C(=O)/C=C/c3ccc(F)cc3)s2)cc1;Active;0.74;0.519;Yes;;396.
1;O=C(Nc1ccc(OCc2nc3ccccc3s2)cc1)Nc1ccc(Cl)c(Cl)c1;Active;0.73;0.793;Yes;;444.34;
1;COc1ccc(C2=NC(=O)/C(=C/c3cc4ccccc4[nH]3)S2)cc1;Active;0.64;0.558;Yes;;334.4;4.2
1;O=C1C=C(O)C(=O)C(CC2CCC3CCCCC3C2)=C1O;Active;0.522;0.4;Yes;;290.36;3.39;74.6;2;
1;CCCCC(Oc1cc(OCCc2ccccc2)cc(OCCc2ccccc2)c1)C(=O)O;Active;0.725;0.51;Yes;;448.56;
1;CC1C(=O)OC2C1CCC1(C)Cc3sc(NC(=O)Nc4ccc(F)cc4F)nc3C(C)C21;Active;0.619;0.196;No;

1;CCCCCCC(Sc1nc(Cl)cc(Nc2ccc3ncccc3c2)n1)C(=O)OCC;Active;0.72;0.503;Yes;;459.02;6.
1;CCCCC(Sc1nc(OCCc2ccccc2)cc(OCCc2ccccc2)n1)C(=O)O;Active;0.88;0.875;Yes;;466.6;5.
1;CCCCCCC(Sc1nc(Cl)cc(Nc2nc(-c3ccccc3)c(C)s2)n1)C(=O)O;Active;0.943;0.794;Yes;;47
1;O=C(O)c1ccc(S(=O)(=O)n2cc(-c3ccc(-c4ccccc4COc4cccc5ccccc45)cc3)nn2)cc1;Inactive
1;CC(=O)Oc1ccc(/C=C/C(=O)OCCCn2cc(CCOC(=O)/C=C/c3ccc(OC(C)=O)c(OC(C)=O)c3)nn2)cc1
1;Cc1ccc(S(=O)(=O)n2cc(C(=O)C(=O)N3CCCC3)c3ccccc32)cc1;Active;0.973;0.62;Yes;;396.
1;COc1cc(/C=C/C(=O)/C=C/c2cc(F)c(O)c(OC)c2)cc(F)c1O;Active;0.887;0.59;Yes;;362.33
1;c1cn2c(NC3CCCCC3)c(-c3ccc(N4CCOCC4)cc3)nc2s1;Active;0.618;0.632;Yes;;382.53;4.6
1;CCCCCCCCCCCCCCC1=C(OC)C(OC)=CC(=O)C1=O;Active;1.0;1.0;Yes;;378.55;6.05;52.6;0;
1;O=C(O/N=C/c1ccc(Br)cc1)c1cc([N+](=O)[O-])cc([N+](=O)[O-])c1;Inactive;0.273;0.41
1;CS(=O)(=O)c1cccc(-c2nc(-c3ccc(F)cc3)c(-c3ccncc3)[nH]2)c1;Active;0.895;0.67;Yes;
1;COc1ccc(/C=C2\SC(c3ccc(N)cc3)=NC2=O)cc1;Active;0.968;0.775;Yes;;310.38;3.34;64.
1;CC(=O)c1cc2c(C)c3c(c(C)c2oc1=O)CC[C@@](C)(CC/C=C(\C)CC/C=C(\C)CCC=C(CO)CO)O3;Ac
1;Cc1nc(NC(=O)c2ccccc2)sc1C(=O)/C=C/c1ccc(N(C)C)cc1;Active;0.7;0.488;Yes;;391.5;4.
1;CCOC(=O)c1c(CSc2cccc(Cl)c2Cl)[nH]c2c1cc(O)c1ccccc12;Active;0.955;0.774;Yes;;446.
1;CC(C)CC(=O)c1c(O)c(C(CC(C)C)C2=C(O)C(C)(C)C(=O)C(C)(C)C2=O)c(O)c(C(CC(C)C)C2=C(
1;O=C(/C=C/c1ccc(O)c(O)c1)OCc1cn(CC(Cn2cc(COC(=O)/C=C/c3ccc(O)c(O)c3)nn2)(Cn2cc(C
1;CC(C)(C)c1cc(/C=C2\CCOC2=O)cc(C(C)(C)C)c1O;Inactive;0.362;0.384;Yes;;302.41;4.3
1;CC(C)(C)c1ccc(CN(C(=O)c2ccc(C(C)(C)C)cc2)c2ccc(C(=O)O)cc2)cc1;Active;0.733;0.59
1;CCCC/C(=C\c1cc(OCCc2ccccc2)cc(OCCc2ccccc2)c1)C(=O)O;Active;0.698;0.468;Yes;;444.
1;COc1cc(/C=C/C(=O)CC(=O)/C=C/c2cc(CC=C(C)C)c(O)c(OC)c2)cc(CC=C(C)C)c1O;Active;0.
1;CCCCCCC(Sc1nc(Cl)cc(Nc2ccc(-c3ccccc3)cc2)n1)C(=O)O;Active;0.927;0.75;Yes;;456.0
1;CCOC(=O)c1c(CCc2ccc(Cl)cc2)[nH]c2ccc(O)cc12;Inactive;0.352;0.721;Yes;;343.81;4.
1;Oc1n[nH]c2ccccc12;Inactive;0.295;0.276;No;Outside AD: prediction should be inte
1;Cc1ccc(C2=NC(=O)/C(=C/c3ccc(OCC(=O)N4CCOCC4)cc3)S2)cc1;Active;0.77;0.674;Yes;;4
1;COC1(c2cc(F)cc(OCc3cc(-c4ccccc4)n(-c4ccc(S(N)(=O)=O)cc4)n3)c2)CCOCC1;Active;0.9
1;NC1=NN(c2cccc(C(F)(F)F)c2)CC1;Inactive;0.365;0.323;No;Outside AD: prediction sh
1;CCC(=O)Nc1ccc(C2=NC(=O)/C(=C/c3ccc(OC)cc3)S2)cc1;Active;0.85;0.665;Yes;;366.44;
1;CCOC(=O)c1c(-c2ccccc2Cl)oc2ccc(O)cc12;Inactive;0.133;0.729;Yes;;316.74;4.64;59.
1;O=C(/C=C/c1ccc(O)c(O)c1)OCc1cn(CC(CO)(Cn2cc(COC(=O)/C=C/c3ccc(O)c(O)c3)nn2)Cn2c
1;O=C1N=C(c2ccc3ccccc3c2)S/C1=C\c1ccc2[nH]ccc2c1;Active;0.645;0.447;Yes;;354.43;5.
1;CCCC/C(=C\c1cc(OCCC2CCCCC2)ccc1OCCC1CCCCC1)C(=O)O;Inactive;0.485;0.557;Yes;;456.
1;CCOC(=O)c1c(CSc2c(C)cccc2C)n(C)c2ccc(O)cc12;Inactive;0.382;0.404;Yes;;369.49;4.
1;O=C(O)c1ccc(N2C(=O)/C(=C\c3ccc(-c4ccc(Br)cc4)o3)C=C2c2ccccc2)cc1;Inactive;0.495
1;O=C1C=C(c2cccc3c2oc2ccccc23)C(=O)C=C1O;Active;0.525;0.337;No;Outside AD: predic
1;COc1cc(C)c2c(c1C)CC(C(C)C(=O)Nc1ccccn1)CC2;Active;0.798;0.524;Yes;;338.45;4.09;
1;CCCOc1cc(C)c2c(c1C)CC(C(C)C(=O)Nc1ncc(C)s1)CC2;Active;0.91;0.656;Yes;;386.56;5.
1;CCCC/C(=C\c1cccc(OCCc2ccccc2)c1OCCc1ccccc1)C(=O)O;Active;0.637;0.489;Yes;;444.5
1;CCOC(=O)c1c(CSc2c(Cl)cccc2Cl)n(C)c2ccc(O)cc12;Inactive;0.411;0.441;Yes;;410.32;
1;CCCOc1cc(C)c2c(c1C)CC(C(C)C(=O)Nc1nccs1)CC2;Active;0.915;0.67;Yes;;372.53;4.93;
1;CCCCC(Sc1ccc(OCCCOc2ccc3ncccc3c2)cc1)C(=O)OCC;Inactive;0.451;0.511;Yes;;453.6;6.
1;CCCCCC[C@@H](Sc1nc(Cl)cc(Nc2ccc(-c3ccc(C#N)cc3)cc2)n1)C(=O)O;Active;0.868;0.632
1;O=C(O)/C=C/c1c(-c2ccc(S(=O)(=O)c3ccccc3)cc2)c(-c2ccccc2)c2n1CCC2;Inactive;0.46;
1;CCOC(=O)c1c(CSc2ccccc2C(F)(F)F)n(C)c2ccc(O)cc12;Inactive;0.428;0.381;Yes;;409.4
1;C/C(=C\CC[C@]1(C)CCc2c(C)c(O)cc(C)c2O1)CC/C=C(\C)CCCC(C)C(=O)O;Active;0.868;0.6
1;CC(=O)N(C)c1ccc(S(=O)(=O)c2cc(C3(C)COC(C)(C)O3)cs2)cc1;Active;0.808;0.636;Yes;;
1;COc1ccc(Sc2cccc(CN(C)c3cc(C(=O)O)nc4cc(Cl)cc(Cl)c34)c2)cc1;Active;0.552;0.281;N
1;O=C(/C=C/c1cccc(O)c1O)OCCc1ccccc1;Active;0.643;0.525;Yes;;284.31;2.9;66.76;2;4;
1;Oc1ccc(-c2ccc(-c3cn(CSc4ccccc4)nn3)cc2)cc1;Active;0.54;0.289;No;Outside AD: pre
1;CCCCC1CCc2cc(O)cc3c(CCN4CCN(c5cc(C)ccn5)CC4)c(C)n1c23;Inactive;0.4;0.34;No;Outs
1;COc1ccc(C2=C/C(=C/c3ccc(O)cc3O)C(=O)O2)cc1;Inactive;0.278;0.384;Yes;;310.31;3.0
1;COc1cc(C)c2c(c1C)CC(C(C)C(=O)NCc1ccco1)CC2;Active;0.534;0.466;Yes;;341.45;3.96;
1;COc1cc(C(=O)Nc2nc3c(s2)C[C@]2(C)CC[C@@H]4[C@H](OC(=O)[C@H]4C)[C@H]2[C@@H]3C)cc(
1;CCOC(=O)c1c(CSc2ccc(OC(F)(F)F)cc2)n(C)c2ccc(O)cc12;Inactive;0.376;0.354;Yes;;42

1;CCCCC(Cc1cc(OCCc2ccccc2)ccc1OCCc1ccccc1)C(=O)O;Active;0.675;0.506;Yes;;446.59;6
1;CCCOc1cc(C)c2c(c1C)CC(C(C)C(=O)Nc1ccccn1)CC2;Active;0.875;0.659;Yes;;366.51;4.8
1;CCCC/C(=C\c1cc(OCCc2ccccc2)ccc1OCCc1ccccc1)C(=O)O;Active;0.693;0.636;Yes;;444.5
1;CCCC/C(=C\c1cc(OCc2ccc(OCCN3CCOCC3)cc2)ccc1OCc1ccc(C(F)(F)F)cc1)C(=O)O;Active;0
1;COc1ccc(C2=NC(=O)/C(=C/c3ccc(OC)c4ccccc34)S2)cc1;Active;0.795;0.615;Yes;;375.45
1;C/C(=C\CC[C@]1(C)CCc2cc(O)cc(C)c2O1)CC/C=C/C(=O)O;Active;0.821;0.616;Yes;;344.4
1;C#CCCCCOC(=O)/C=C/c1ccc(OC(C)=O)c(OC(C)=O)c1;Active;0.521;0.522;Yes;;344.36;2.9
1;CC(C)C(=O)c1c(O)c([C@@H](C2=C(O)C(C)(C)C(=O)C(C)(C)C2=O)C(C)C)c(O)c2c1OC1=C(C(=
1;C/C(=C\CC/C(C)=C/CC[C@]1(C)CCc2c(C)c(O)c(C)c(C)c2O1)CC/C=C(\C)C(=O)N1CCCCC1;Act
1;CCc1cc(/C=C/c2ccc(OC)cc2)cc(CC)c1O;Active;0.613;0.549;Yes;;282.38;4.7;29.46;1;2
1;O=C(NCC12C[C@H]3C[C@@H](C1)C[C@@H](C2)C3)Nc1ccc2c(c1)CCN2Cc1ccc(C(=O)O)cc1;Inac
1;Cc1cc(CCc2ccc(F)cc2)cc(C)c1O;Active;0.793;0.421;Yes;;244.31;3.93;20.23;1;1;3;0.
1;CC(C)c1cc(/C=C/c2cccs2)cc(C(C)C)c1O;Active;0.642;0.541;Yes;;286.44;5.87;20.23;1
1;COC(=O)CCCn1ccc2cc(NC(=O)NCC34C[C@H]5C[C@@H](C3)C[C@@H](C4)C5)ccc21;Active;0.62
1;CCCC/C(=C\c1cc(C)c(O)c(C)c1)c1ccc(F)cc1;Active;0.633;0.429;Yes;;298.4;5.88;20.2
1;Cc1ccc(/C=C/c2cc(C)c(O)c(C)c2)s1;Active;0.9;0.552;Yes;;244.36;4.55;20.23;1;2;2;
1;Cc1ccc(CN(c2ccc(C(=O)O)cc2)S(=O)(=O)c2ccc(-c3ccccc3)cc2)cc1;Active;0.51;0.601;Y
1;COc1ccc(C2OCC(=CCC/C(C)=C/CC/C(C)=C/CC[C@]3(C)CCc4cc(OCCCC(=O)O)cc(C)c4O3)CO2)c
1;C/C(=C\CC/C(C)=C/CC[C@]1(C)CCc2c(CN3CCN(C)CC3)c(O)c(CN3CCN(C)CC3)c(C)c2O1)CCC=C
1;Cc1cc(O)cc2c1O[C@](C)(CC/C=C/C(=O)O)CC2;Active;0.689;0.505;Yes;;276.33;3.21;66.
1;NC(=O)C1(c2cccc(Sc3ccc(-n4cnnc4)cc3)c2)CCOCC1;Active;0.579;0.573;Yes;;380.47;2.
1;Cc1cc(Cc2cccnc2)cc(C)c1O;Active;0.635;0.39;Yes;;213.28;2.99;33.12;1;2;2;0.831;1
1;Cc1cc(/C=C/c2ccc(F)cc2F)cc(C)c1O;Active;0.882;0.552;Yes;;260.28;4.46;20.23;1;1;
1;CCc1cc(/C=C/c2cccnc2)cc(CC)c1O;Active;0.672;0.433;Yes;;253.34;4.08;33.12;1;2;4;
1;Cc1cc(/C=C/c2ccccc2Br)cc(C)c1O;Active;0.873;0.581;Yes;;303.2;4.94;20.23;1;1;2;0
1;Cc1cc(O)cc2c1O[C@](C)(CCCC(C)CCCC(C)CCCC(C)C(=O)O)CC2;Active;0.66;0.417;Yes;;43
1;Cc1ccc(/C=C/c2cc(C(C)(C)C)c(O)c(C(C)(C)C)c2)cc1;Active;0.668;0.47;Yes;;322.49;6
1;O=C(Nc1ccc2c(ccn2Cc2ccc(F)cc2)c1)NC12C[C@H]3C[C@@H](C1)C[C@@H](C2)C3;Active;0.5
1;Cc1cc(/C=C/C(=O)c2ccc(F)cc2)cc(C)c1O;Active;0.593;0.533;Yes;;270.3;4.04;37.3;1;
1;Cc1cc(/C=C/c2cccc3ccccc23)cc(C)c1O;Active;0.802;0.549;Yes;;274.36;5.33;20.23;1;
1;Cc1cc(CCC(=O)c2ccc(F)cc2)cc(C)c1O;Active;0.657;0.379;Yes;;272.32;3.96;37.3;1;2;
1;Cc1nccn1-c1ccc(COc2cc(F)cc(C3(O)CCOCC3)c2)cc1;Active;0.63;0.788;Yes;;382.44;3.9
1;COC(=O)/C(C)=C/CC/C(C)=C/CC/C(C)=C/CC[C@]1(C)CCc2c(c(C)cc(O)c2C(=O)OC)O1;Active
1;C/C(=C\CC/C(C)=C/CC[C@]1(C)CCc2c(C)c(O)cc(C)c2O1)CC/C=C(\C)C(=O)N1CCCCC1;Active
1;Cc1cc(OCCN(C)C)c(C)c2c1CCC(C(C)C(=O)Nc1nccs1)C2;Active;0.88;0.562;Yes;;401.58;4
1;COc1cc(/C=C/c2cccs2)cc(F)c1O;Active;0.873;0.644;Yes;;250.29;3.77;29.46;1;3;3;0.
1;Cc1cc(/C=C/c2cc(F)cc(F)c2)cc(C)c1O;Active;0.912;0.593;Yes;;260.28;4.46;20.23;1;
1;COC(=O)C(C)CCC/C(C)=C/CC/C(C)=C/CC[C@]1(C)CCc2cc(OC)cc(C)c2O1;Active;0.802;0.50
1;CCCCCCCCCCCCCCCC1=C(O)C(=O)C=C(OC)C1=O;Active;0.993;1.0;Yes;;378.55;6.35;63.6;
0;CCCCCc1cc(OC(C)=O)cc(OC)c1OC(C)=O;Inactive;0.449;0.552;Yes;;294.35;3.28;61.83;0
0;Oc1ccc(CCC2CCC(c3ccc(O)cc3)CO2)cc1;Inactive;0.366;0.276;No;Outside AD: predicti
0;C=CCc1ccc(O)c(-c2ccc(OC)c(/C=C\C)c2)c1;Active;0.545;0.348;No;Outside AD: predic
0;CCCCC(Sc1nc(Cl)cc(Nc2ccc3ncccc3c2)n1)C(=O)OCC;Active;0.703;0.462;Yes;;430.96;5.
0;O=C(O)c1ccc(N(Cc2ccc3c(c2)OCCO3)S(=O)(=O)c2ccc(-c3ccccc3)cc2)cc1;Inactive;0.399
0;CCCCNC(=O)N(O)CC1COc2ccccc2O1;Inactive;0.258;0.583;Yes;;280.32;2.03;71.03;2;4;5
0;CC(C)(C)c1cc(/C=C/c2cccs2)cc(C(C)(C)C)c1O;Active;0.62;0.571;Yes;;314.49;6.22;20
0;CCOC(=O)C(Sc1nc(Cl)cc(Nc2ccc3ncccc3c2)n1)c1ccccc1;Active;0.733;0.475;Yes;;450.9
0;CCOC(=O)c1c(NC(C)c2ccccc2)[nH]c2ccc(O)cc12;Inactive;0.229;0.526;Yes;;324.38;4.2
0;CCC(OC)(c1cccc(COc2ccc3ccccc3c2)c1)c1nccs1;Inactive;0.215;0.693;Yes;;389.52;6.1
0;CCCCCC(=O)c1c(O)c(C(C2=C(O)C(C)(C)C(=O)C(C)(C)C2=O)C(C)C)c(O)c2c1OC1=C(C(=O)C(C
0;O=C(O)/C=C/c1c(-c2ccc(S(=O)(=O)Cc3ccccc3)cc2)c(-c2ccccc2)c2n1CCC2;Inactive;0.44
0;COc1cccc(CN(C(=O)c2ccc(-c3ccccc3)cc2)c2ccc(C(=O)O)cc2)c1;Active;0.81;0.593;Yes;
0;CC(=O)Oc1ccc(/C=C/C(=O)CCCN=[N+]=[N-])cc1OC(C)=O;Inactive;0.473;0.402;Yes;;331.
0;Cc1ccc(Sc2nc([N+](=O)[O-])nn2CC(=O)c2ccccc2)cc1;Inactive;0.424;0.269;No;Outside

```
0;CCCc1ccc(O)c(-c2ccc(OC)c(CCC)c2)c1;Active;0.59;0.359;Yes;;284.4;4.97;29.46;1;2;
0;CCCCCCC(Sc1nc(Cl)cc(NCc2ccc3ncccc3c2)n1)C(=O)OCC;Active;0.633;0.442;Yes;;473.04
0;CCCCCCC1=CC(=O)C(O)=CC1=O;Active;0.627;0.556;Yes;;208.26;2.48;54.37;1;3;5;0.557
0;Cc1ccc2nc(-c3ccc(OCC4CCN(C(=O)Nc5ccc(Cl)cc5)CC4)cc3)c(NC3CCCCC3)n2c1;Active;0.7
0;CC(C)(C)CNC(=S)Nc1ccc2c(c1)CCN2Cc1ccc(-c2ccccc2)cc1;Inactive;0.405;0.763;Yes;;4
0;CCCCCC[C@H](Sc1nc(Cl)cc(Nc2ccc(-c3ccc(C#N)cc3)cc2)n1)C(=O)O;Active;0.868;0.632;
0;C=CCc1ccc(O)c(-c2ccc(OC)c(CC=C)c2)c1;Active;0.573;0.374;Yes;;280.37;4.52;29.46;
0;CCCCCCN(c1ccc(C(=O)O)cc1)S(=O)(=O)c1ccc(-c2ccccc2)cc1;Active;0.781;0.575;Yes;;4
0;CCCCCCC(Sc1nc(Cl)cc(Nc2cc(OCC(F)(F)F)cc(OCC(F)(F)F)c2)n1)C(=O)O;Active;0.661;0.
0;Cc1ccc2nc(-c3ccc(OCC4CCN(C(=O)Nc5ccc(Cl)cc5)CC4)cc3)c(NC(C)C)n2c1;Active;0.72;0
0;CC(C)(C)c1ccc(CN2CCN(C(=O)CCc3cc(-c4ccc(F)cc4)n(-c4ccc(Cl)nn4)n3)CC2)cc1;Active
0;CC1CN(c2cccc(Cl)n2)NC(=O)N1;Inactive;0.037;0.69;Yes;;226.67;1.16;57.26;2;3;1;0.
0;CCCCCCCCCCCCCCCC1=C(OC)C(=O)C=C(OC)C1=O;Active;1.0;1.0;Yes;;392.58;6.44;52.6;0
0;CCCC/C(=C\c1ccc(OCCCOc2cccc(C)c2C)cc1)C(=O)O;Inactive;0.497;0.497;Yes;;382.5;5.
0;CCCCCC(=O)c1c(O)cc(O)c(C(C2=C(O)C(C)(C)C(=O)C(C)(C)C2=O)C(C)C)c1O;Inactive;0.16
0;CC(C)(C)c1cc(/C=C/c2cccnc2)cc(C(C)(C)C)c1O;Active;0.662;0.429;Yes;;309.45;5.55;
0;CCCCCCC(Sc1nc(Cl)cc(Oc2ccc3ncccc3c2)n1)C(=O)OCC;Active;0.717;0.419;Yes;;460.0;6.
0;Cc1ccccc1CCn1cc(-c2ccc(-c3ccc(N)cc3)cc2)nn1;Active;0.574;0.255;No;Outside AD: p
0;O=C1Nc2c(Br)cccc2/C1=C1/Nc2ccccc2/C1=N\O;Inactive;0.289;0.31;No;Outside AD: pre
0;CCOC(=O)c1c(CCc2cccc(Cl)c2)[nH]c2ccc(O)cc12;Inactive;0.163;0.724;Yes;;343.81;4.
0;O=C(O)c1ccc(N(Cc2ccc3ccccc3c2)C(=O)c2ccc(-c3ccccc3)cc2)cc1;Active;0.715;0.631;Y
0;CC(=O)c1c(O)cc(O)c(C(CC(C)C)c2c(O)cc(O)c(C(C)=O)c2O)c1O;Inactive;0.242;0.356;Ye
0;CCCc1ccc(O)c(-c2ccc(O)c(CCC)c2)c1;Active;0.557;0.287;No;Outside AD: prediction
0;Cc1ccc(-c2cc(C(F)(F)F)nn2-c2ccc(S(N)(=O)=O)cc2)cc1;Active;1.0;0.977;Yes;;381.38
0;CCOC(=O)C(Sc1nc(Cl)cc(Nc2cccc(C)c2C)n1)c1ccccc1;Active;0.744;0.446;Yes;;427.96;
0;CCOC(=O)c1c(CSc2cc(C)cc(C)c2)n(C)c2ccc(O)cc12;Inactive;0.398;0.38;Yes;;369.49;4.
0;O=C(O)C(Sc1nc(Cl)cc(NCCCc2ccccc2)n1)c1cccc2ccccc12;Active;0.87;0.672;Yes;;463.9
0;C#CCCOC(=O)/C=C/c1ccc(OC(C)=O)c(OC(C)=O)c1;Active;0.543;0.499;Yes;;316.31;2.12;
0;c1cn2c(NC3CCCCC3)c(-c3ccc(N4CCOCC4)cc3)nc2cn1;Inactive;0.49;0.686;Yes;;377.49;3.
0;O=C(O)/C=C/c1c(-c2ccc([S+]([O-])c3ccccc3)cc2)c(-c2ccccc2)c2n1CCC2;Inactive;0.36
0;CC(C)=CCC/C(C)=C/CC1=C(O)C(=O)C=C(O)C1=O;Active;0.603;0.57;Yes;;276.33;3.48;74.
0;COc1ccc(/C=C2\OC(c3ccc(C)cc3)=NC2=O)cc1;Inactive;0.371;0.552;Yes;;293.32;3.35;4
0;O=C(O)c1cc(-c2ccc(-c3ccc(-c4cc(C(=O)O)c5cc(Cl)ccc5n4)cc3)cc2)nc2ccc(Cl)cc12;Ina
0;Cc1ccc(-c2cc(CCC(=O)Oc3ccc(C(C)C)cc3)nn2-c2ccccc2)cc1;Active;0.67;0.405;Yes;;42
0;CC1C(=O)OC2C1CCC1(C)Cc3sc(NC(=O)c4ccc5c(c4)OCO5)nc3C(C)C21;Active;0.531;0.233;N
0;O=C(O)/C=C/c1c(-c2ccc(Nc3ccccc3)cc2)c(-c2ccccc2)c2n1CCC2;Inactive;0.497;0.308;N
0;CCCCC/C=C\C/C=C\C=C\C=C\[C@@H]1O[C@H]1CCCC(=O)O;Inactive;0.38;0.545;Yes;;318.46
0;CCCCCCC(Sc1nc(Cl)cc(Nc2cccc(C)c2C)n1)C(=O)O;Active;0.79;0.629;Yes;;407.97;6.01;
0;CCCCC(Cc1ccc(OCCCOc2cccc(C)c2C)cc1)C(=O)O;Inactive;0.258;0.597;Yes;;384.52;5.58
0;C/C=C(/C=O)[C@@H](CC=O)CC(=O)OCCc1ccc(O)c(O)c1;Active;0.68;0.324;No;Outside AD:
0;Oc1c(F)c(F)cc(-c2nc(-c3ccc(-c4ccsc4)cc3)no2)c1F;Active;0.591;0.239;No;Outside A
0;COc1c(CC=C(C)C)c(O)c(C(CC(C)C)c2c(O)c(C(C)=O)c(O)c3c2OC(C)(C)C(O)C3)c(O)c1C(C)=
0;C=CCc1ccc(O)c(-c2ccc(OC)c(/C=C/C)c2)c1;Active;0.545;0.348;No;Outside AD: predic
0;O=C(O)c1ccc(N(Cc2cnc3ccccc3c2)S(=O)(=O)c2ccc(-c3ccccc3)cc2)cc1;Active;0.53;0.59
0;Oc1ccc(-c2cccc3c2Sc2ccccc2S3)c(O)c1;Inactive;0.359;0.253;No;Outside AD: predict
0;O=C(/C=C/C=C/c1ccc(O)c(O)c1)N1CCCCC1;Active;0.591;0.357;Yes;;273.33;2.68;60.77;
0;O=C(O)/C=C/c1c(-c2ccc(Cc3ccccc3)cc2)c(-c2ccccc2)c2n1CCC2;Inactive;0.455;0.295;N
0;CCCOc1ccc(/C=C2\SC(c3ccc(OC)cc3)=NC2=O)cc1;Active;0.925;0.712;Yes;;353.44;4.55;
0;CCCCC1=C(OC)C(OC)=CC(=O)C1=O;Active;0.693;0.879;Yes;;224.26;1.76;52.6;0;4;5;0.5
0;O=C(O)/C=C/c1c(-c2ccc([S+]([O-])Cc3ccccc3)cc2)c(-c2ccccc2)c2n1CCC2;Inactive;0.4
0;C=CCc1ccc(OC(C)=O)c(-c2ccc(OC)c(CC=C)c2)c1;Inactive;0.47;0.328;No;Outside AD: p
0;CCOC(=O)c1c(CSc2ccc(OC)cc2)n(C)c2ccc(O)cc12;Inactive;0.381;0.368;Yes;;371.46;4.
0;O=C(c1ccccc1)c1sc(N2CCOCC2)nc1-c1ccccc1;Active;0.679;0.617;Yes;;350.44;3.88;42.
0;CCOC(=O)c1c(N2CCN(c3ccc(Cl)cc3)CC2)[nH]c2ccc(O)cc12;Inactive;0.255;0.569;Yes;;3
```

0;O=C(/C=C/C=C/c1cc(O)c(O)c(O)c1)N1CCCCC1;Inactive;0.476;0.256;No;Outside AD: pre
0;CCOC(=O)c1c(Nc2ccccc2)[nH]c2ccc(O)cc12;Inactive;0.18;0.565;Yes;;296.33;3.79;74.
0;Oc1ccc(Cc2ccc3ccccc3c2)c(O)c1;Inactive;0.395;0.343;No;Outside AD: prediction sh
0;COc1c(CC=C(C)C)c(O)c(C(CC(C)C)c2c(O)c(CC=C(C)C)c(O)c(C(C)=O)c2O)c(O)c1C(C)=O;In
0;CCCCNc1cc(Cl)nc(SC(C(=O)O)c2cccc3ccccc23)n1;Active;0.843;0.658;Yes;;401.92;5.41
0;CC(=O)c1c(O)c(C(CC(C)C)c2c(O)c3c(c(C(C)=O)c2O)OC(C)(C)C=C3)c(O)c2c1OC(C)(C)C=C2
0;Cc1ccc(S(=O)(=O)N(Cc2ccc(Cl)c(Cl)c2)c2ccc(C(=O)O)cc2)cc1;Active;0.575;0.484;Yes
0;CCCCC(Sc1nc(OCCC2CCCCC2)cc(OCCC2CCCCC2)n1)C(=O)O;Active;0.735;0.838;Yes;;506.
0;CCOC(=O)C1CCN(C(=O)CCc2cc(-c3ccc(C)cc3)n(-c3ccccc3)n2)CC1;Active;0.653;0.392;Ye
0;COc1ccc(-c2nc(O)c(-c3ccccc3)s2)cc1;Active;0.775;0.694;Yes;;283.35;4.19;42.35;1;
0;CCOC(=O)c1c(CSc2ccccc2)n(C)c2ccc(O)cc12;Inactive;0.378;0.414;Yes;;341.43;4.35;5
0;CC(C)(C)c1ccc(CN2CCN(C(=O)CCc3cc(-c4ccc(F)cc4)n(-c4ccccc4)n3)CC2)cc1;Active;0.7
0;O=C(O)/C=C/c1c(-c2ccc(Sc3ccccc3)cc2)c(-c2ccccc2)c2n1CCC2;Inactive;0.455;0.295;N
0;O=C(O)/C=C/c1c(-c2ccc(CCc3ccccc3)cc2)c(-c2ccccc2)c2n1CCC2;Inactive;0.43;0.295;N
0;C=C(C)[C@@H](O)Cc1c(O)c(C(C)=O)c(O)c(C(CC(C)C)c2c(O)c(CC=C(C)C)c(OC)c(C(C)=O)c2
0;O=C(O)C(Sc1nc(Cl)cc(NCCc2ccccc2)n1)c1cccc2ccccc12;Active;0.86;0.695;Yes;;449.96
0;CC(C)CCNc1cc(Cl)nc(SC(C(=O)O)c2cccc3ccccc23)n1;Active;0.865;0.663;Yes;;415.95;5
0;CC(C)=CCC/C(C)=C/CC/C(C)=C/CC1=C(O)C(=O)C=C(O)C1=O;Active;0.56;0.578;Yes;;344.4
0;CC(c1cc2c(Cl)cccc2s1)N(O)C(N)=O;Inactive;0.245;0.629;Yes;;270.74;3.39;66.56;2;3
0;CCOC(=O)CSc1nc(Cl)cc(Nc2ccc3ncccc3c2)n1;Active;0.642;0.372;Yes;;374.85;4.08;77.
0;O=C1Nc2ccc(Br)cc2/C1=C1/Nc2ccccc2/C1=N\O;Inactive;0.151;0.347;No;Outside AD: pr
0;CC(C)(C)c1ccc(CN2CCN(C(=O)CCc3cc(-c4ccc(F)cc4)n(-c4ccc(Cl)cc4)n3)CC2)cc1;Active
0;CC1(C)Cc2c(c(C(=O)Nc3ccc(C(F)(F)F)cc3)cc3nc(Nc4c(Cl)cccc4Cl)[nH]c23)O1;Active;0.
0;CC(=O)Oc1c(C)c(C)c2c(c1C)CCC(C)(C(=O)OCCCN=[N+]=[N-])O2;Active;0.637;0.254;No;O
0;O=C(O)c1ccc(N(CC2CCCCC2)S(=O)(=O)c2ccc(Oc3ccccc3)cc2)cc1;Active;0.66;0.445;Yes;
0;CC(C)(C)c1cc(/C=C/c2ccc(Cl)s2)cc(C(C)(C)C)c1O;Active;0.59;0.361;Yes;;348.94;6.8
0;COc1cc(/C=C2\SC(c3ccc(C)cc3)=NC2=O)cc(Cl)c1O;Active;0.677;0.689;Yes;;359.83;4.4
0;CCCCCC(=O)c1c(O)c(C(C2=C(O)CC(C)(C)CC2=O)C(C)C)c(O)c(C(C2=C(O)CC(C)(C)CC2=O)C(C
0;Cc1cc(Oc2ccc(Cl)cc2NC(=O)Nc2cccc(C(F)(F)F)c2)cc(S(=O)(=O)O)c1Cl;Active;0.605;0.
0;CC(=O)O[C@@H]1CC[C@@]2(C)[C@@H](CC[C@]3(C)[C@@H]2C(=O)C=C2[C@@H]4[C@@H](C)[C@H]
0;Cc1ccc(-c2cc(CCC(=O)N3CCN(Cc4ccc(C(C)(C)C)cc4)CC3)nn2-c2ccccc2)cc1;Active;0.691
0;CC(Nc1[nH]c2ccc(O)cc2c1C#N)c1ccccc1;Inactive;0.239;0.28;No;Outside AD: predicti
0;Cc1ccc(-c2cc(CCC(=O)N3CCN(Cc4ccc(C(C)(C)C)cc4)CC3)nn2-c2ccc(Cl)nn2)cc1;Active;0.
0;CC(C)C(C1=C(O)CC(C)(C)CC1=O)c1c(O)c(C(C2=C(O)CC(C)(C)CC2=O)C(C)C)c(O)c(C(C2=C(O
0;Cc1ccc(-n2c(=O)c3cc4c(=O)n(-c5ccc(C)c(C)c5)c(=O)c4c(C(=O)c4ccc(C(=O)O)cc4)c3c2=
0;CN(C)c1cccc2c(S(=O)(=O)Nc3ccc(-c4ccc(-c5cn(CCCc6ccccc6)nn5)cc4)cc3)cccc12;Activ
0;Oc1ccc(-c2ccccc2)c(O)c1;Inactive;0.297;0.351;No;Outside AD: prediction should b
0;CC(C)(C)c1ccc(CN2CCN(C(=O)CCc3cc(-c4ccc(F)cc4)n(-c4ccc5ccccc5n4)n3)CC2)cc1;Acti
0;CCCCCCc1ccc(O)cc1O;Inactive;0.485;0.456;Yes;;194.27;3.22;40.46;2;2;5;0.706;1.08
0;O=C(O)/C=C/c1c(-c2ccc(SCc3ccccc3)cc2)c(-c2ccccc2)c2n1CCC2;Inactive;0.43;0.273;N
0;CC(C)(C)c1ccc(CN2CCN(C(=O)CCc3cc(-c4ccc(C(F)(F)F)cc4)n(-c4ccccc4)n3)CC2)cc1;Act
0;CCOC(=O)c1c(CSc2ccccc2OC)n(C)c2ccc(O)cc12;Inactive;0.363;0.424;Yes;;371.46;4.36
0;Oc1nc(-c2cccnc2)sc1-c1ccccc1;Active;0.67;0.512;Yes;;254.31;3.58;46.01;1;4;2;0.7
0;Cc1ccc(-c2cc(CCC(=O)N3CCN(Cc4ccc(C(C)(C)C)cc4)CC3)nn2-c2ccc(Cl)cc2)cc1;Active;0.
0;CC(C)(C)c1ccc(CN2CCN(C(=O)CCc3cc(-c4ccc(C(F)(F)F)cc4)n(-c4ccc(Cl)nn4)n3)CC2)cc1
0;COc1cc(/C=C2\SC(c3ccccc3)=NC2=O)cc([N+](=O)[O-])c1O;Active;0.85;0.589;Yes;;356.
0;CCCOc1cc(C)c2c(c1C)CC(C(C)C(=O)NCc1cccs1)CC2;Inactive;0.312;0.637;Yes;;385.57;5
0;CCCCC/C=C\C/C=C\C=C\C=C\C1CC1CCCC(=O)O;Active;0.818;0.671;Yes;;316.49;6.07;37.3
0;CCCCCCCCC1=C(O)C(=O)C=C(OC)C1=O;Active;0.993;1.0;Yes;;266.34;3.23;63.6;1;4;8;0.
0;CC(C)(C)c1ccc(CN2CCN(C(=O)CCc3cc(-c4ccc(C(F)(F)F)cc4)n(-c4ccc5ccccc5n4)n3)CC2)c
0;CC1C(=O)OC2C1CCC1(C)Cc3sc(NC(=O)NCc4ccccc4)nc3C(C)C21;Inactive;0.433;0.245;No;O
0;C[C@H]1[C@H](C)CC[C@]2(C)CC[C@]3(C)C(=CC(=O)[C@@H]4[C@@]5(C)CC[C@@H](O)[C@](C)(
0;O=C(O)C(Sc1nc(Cl)cc(Nc2ccc(F)cc2)n1)c1cccc2ccccc12;Active;0.873;0.816;Yes;;439.
0;CCOC(=O)c1c(N2CCN(c3ccccc3)CC2)[nH]c2ccc(O)cc12;Inactive;0.208;0.531;Yes;;365.4

0;O=C(c1ccccc1)c1sc(N2CCCCC2)nc1-c1ccccc1;Active;0.607;0.472;Yes;;348.47;5.03;33.
0;O=C1Nc2c(Cl)cccc2/C1=C1/Nc2ccccc2/C1=N\O;Inactive;0.357;0.295;No;Outside AD: pr
0;O=C(O)c1ccc(N(CC2CCCCC2)S(=O)(=O)c2ccc(-c3ccccc3)cc2)cc1;Active;0.54;0.483;Yes;
0;O=C(O)c1ccc(N(Cc2ccc3c(c2)OCO3)S(=O)(=O)c2ccc(-c3ccccc3)cc2)cc1;Inactive;0.412;
0;COc1cc(CCC(=O)CC(=O)CCc2ccc(O)c(OC)c2)ccc1O;Inactive;0.426;0.525;Yes;;372.42;3.
0;CCOC(=O)c1c(CSc2ccc(F)cc2F)n(C)c2ccc(O)cc12;Inactive;0.418;0.383;Yes;;377.41;4.
0;CCOC(=O)CSc1nc(Cl)cc(Nc2cccc3c2CCC3)n1;Active;0.597;0.355;Yes;;363.87;4.02;64.1
0;COc1ccc(-n2nc(CCC(=O)N3CCN(Cc4ccc(C(C)(C)C)cc4)CC3)cc2-c2ccc(C)cc2)nn1;Active;0.
0;O=C1Nc2c(F)cccc2/C1=C1/Nc2ccccc2/C1=N\O;Inactive;0.446;0.29;No;Outside AD: pred
0;O=C(O)c1ccc(N(Cc2ccccc2)S(=O)(=O)c2ccc(-c3ccccc3)cc2)cc1;Inactive;0.49;0.643;Ye
0;COc1ccc(C(=O)NC(=S)N2CCCCC2)cc1;Inactive;0.485;0.344;No;Outside AD: prediction
0;COc1ccc(/C(C)=C2\SC(c3ccc(N)cc3)=NC2=O)cc1;Active;0.555;0.525;Yes;;324.41;3.73;
0;CS(=O)(=O)Nc1ccc(-c2cn3c(c2-c2ccccc2)CCC3)cc1;Inactive;0.302;0.514;Yes;;352.46;
0;COC(=O)CNC(=O)C(=O)c1c[nH]c2ccccc12;Inactive;0.31;0.363;Yes;;260.25;0.64;88.26;
0;Cc1ccc(S(=O)(=O)N(Cc2ccc(Oc3ccccc3)cc2)c2ccc(C(=O)O)cc2)cc1;Active;0.713;0.542;
0;Cc1ccc(-c2cc(CCC(=O)N3CCN(Cc4ccc(C(C)(C)C)cc4)CC3)nn2-c2ccc3ccccc3n2)cc1;Active
0;COc1ccc(C2=NC(=O)/C(=C/c3ccc4ccccc4c3)S2)cc1;Active;0.902;0.704;Yes;;345.42;4.9
0;CC(=O)c1c(O)c(C/C=C(/C)CCC=C(C)C)c(O)c(C(CC(C)C)c2c(O)c(C/C=C(\C)CCC=C(C)C)c(O)
0;CCOCc1ccc(C(C)N(O)C(N)=O)o1;Active;0.615;0.592;Yes;;228.25;1.65;88.93;2;4;5;0.5
0;CCOC(=O)c1c(CSc2cccc(Cl)c2)n(C)c2ccc(O)cc12;Inactive;0.378;0.454;Yes;;375.88;5.
0;O=C(NC(=S)N1CCCCC1)c1ccc(F)cc1;Inactive;0.497;0.33;No;Outside AD: prediction sh
0;O=C(O)CSc1nc(Cl)cc(Nc2cccc3c2CCC3)n1;Active;0.576;0.397;Yes;;335.82;3.54;75.11;
0;COc1cc(C)c2c(c1C)CC(C(C)C(=O)NC(C)(C)C)CC2;Active;0.526;0.444;Yes;;317.47;3.97;
0;CCCCCCC1=C(O)C(=O)C=C(O)C1=O;Active;0.983;1.0;Yes;;224.26;2.36;74.6;2;4;5;0.555
0;CCOC(=O)C1CCN(C(=O)CCc2cc(-c3ccc(C)cc3)n(-c3ccc4ccccc4n3)n2)CC1;Active;0.63;0.3
0;CCCCN(c1ccc(C(=O)O)cc1)S(=O)(=O)c1ccc(-c2ccccc2)cc1;Active;0.706;0.569;Yes;;409
0;COc1ccccc1CC(=O)Nc1nc2c(s1)CC1(C)CCC3C(C)C(=O)OC3C1C2C;Inactive;0.4;0.246;No;Ou
0;COC1=CC(=O)C(O)=C(C/C=C(\C)CCC=C(C)C)C1=O;Inactive;0.4;0.776;Yes;;290.36;3.56;6
0;Cc1ccsc1C(C)N(O)C(N)=O;Inactive;0.217;0.521;Yes;;200.26;1.89;66.56;2;3;2;0.565;
0;O=C(O)/C=C/c1c(-c2ccccc2)c(-c2ccccc2)c2n1CCC2;Inactive;0.292;0.328;No;Outside A
0;CCOC(=O)c1c(CSc2ccc(C(F)(F)F)cc2)n(C)c2ccc(O)cc12;Inactive;0.403;0.365;Yes;;409
0;O=C1NCc2c1cc1cc3c(cc1c2-c1ccc2c(c1)OCO2)OCO3;Active;0.72;0.57;Yes;;347.33;3.21;
0;C=CCc1ccc(O)c(-c2ccc(O)c(CC=C)c2)c1;Active;0.552;0.308;No;Outside AD: predictio
0;O=C(O)c1ccc(N(Cc2cccs2)S(=O)(=O)c2ccc(-c3ccccc3)cc2)cc1;Inactive;0.485;0.545;Ye
0;O=C(O)C(Sc1nc(Cl)cc(Nc2ccc3ncccc3c2)n1)c1cccc2ccccc12;Active;0.92;0.736;Yes;;47
0;CCCCCCCCN(c1ccc(C(=O)O)cc1)S(=O)(=O)c1ccc(C)cc1;Active;0.696;0.45;Yes;;403.54;5
0;COc1c(CC=C(C)C)c(O)c(C(CC(C)C)c2c(O)c3c(c(C(C)=O)c2O)OC(C(C)(C)O)C3)c(O)c1C(C)=
0;C=CCc1ccc(OC(C)=O)c(-c2ccc(OC(C)=O)c(CC=C)c2)c1;Inactive;0.432;0.282;No;Outside
0;O=C(O)/C=C/c1c(-c2ccc(-c3ccccc3)cc2)c(-c2ccccc2)c2n1CCC2;Inactive;0.443;0.321;N
0;O=C1C=C(c2cccc([N+](=O)[O-])c2)C(=O)C=C1O;Inactive;0.395;0.323;No;Outside AD: p
0;COc1ccc(/C=C2\SC(c3ccc([N+](=O)[O-])c(O)c3)=NC2=O)cc1;Active;0.752;0.616;Yes;;3
0;CCCCC(Cc1cc(OCc2ccccc2)cc(OCc2ccccc2)c1)C(=O)O;Active;0.71;0.529;Yes;;446.59;
0;Cc1ccc(NS(=O)(=O)c2cc(C(=O)Nc3ccccc3C(=O)O)ccc2Br)cc1;Inactive;0.436;0.276;No;O
0;CC(C)(C)c1cc(C(=O)c2cccs2)cc(C(C)(C)C)c1O;Inactive;0.22;0.419;Yes;;316.47;5.28;
0;CCCCC(Sc1ccc(OCCCOc2cccc(OC)c2)cc1)C(=O)O;Inactive;0.19;0.715;Yes;;404.53;5.28;
0;Cc1ccc(S(=O)(=O)N(Cc2ccc3ccccc3c2)c2ccc(C(=O)O)cc2)cc1;Inactive;0.475;0.636;Yes
0;CCCCC(Sc1ccc(OCCCOc2ccccc2C)cc1)C(=O)O;Inactive;0.163;0.809;Yes;;388.53;5.58;55
0;CCCCC(Sc1ccc(OCCCOc2cccc(C)c2)cc1)C(=O)O;Inactive;0.237;0.727;Yes;;388.53;5.58;
0;Cc1ccc(/C=C/C(=O)NCCCCN2CCN(C(c3ccccc3)c3ccccc3)CC2)cn1;Active;0.594;0.416;Yes;
0;Oc1ccc(/C=C/c2cc(O)cc(O)c2)cc1;Inactive;0.427;0.595;Yes;;228.25;2.97;60.69;3;3;
0;CCOC(=O)c1c(Nc2ccc(C)cc2)[nH]c2ccc(O)cc12;Inactive;0.287;0.53;Yes;;310.35;4.1;7
0;CCOC(=O)c1c(CSc2ccc(F)cc2)n(C)c2ccc(O)cc12;Inactive;0.453;0.387;Yes;;359.42;4.4
0;C=C(C)C1Cc2c(O)c(C(CC(C)C)c3c(O)c4c(c(C(C)=O)c3O)OC(C(=C)C)C4)c(O)c(C(C)=O)c2O1
0;Cc1cccc(Nc2cc(Cl)nc(SC(C(=O)O)c3cccc4ccccc34)n2)c1C;Active;0.912;0.702;Yes;;449

0;O=C(O)c1ccc(-c2ccc(-c3cn(Cc4ccc(C(=O)O)cc4[N+](=O)[O-])nn3)cc2)cc1;Inactive;0.4
0;O=C(O)/C=C/c1c(-c2ccc(Nc3ccc(Cl)cc3)cc2)c(-c2ccccc2)c2n1CCC2;Active;0.517;0.307
0;CCCCC(Sc1ccc(OCCCOc2ccc(C)cc2)cc1)C(=O)O;Inactive;0.253;0.725;Yes;;388.53;5.58;
0;Nc1ccc(-c2c(-c3ccccc3)c3n(c2/C=C/C(=O)O)CCC3)cc1;Inactive;0.438;0.301;No;Outsid
0;COc1c(CC=C(C)C)c(O)c(C(CC(C)C)c2c(O)c(C(C)=O)c(O)c3c2OC(C(C)(C)O)C3)c(O)c1C(C)=
0;COc1c(CC=C(C)C)c(O)c(C(CC(C)C)c2c(O)c3c(c(C(C)=O)c2O)OC(C)(C)C(O)C3)c(O)c1C(C)=
0;COc1cc(CCC(=O)CCCCc2ccccc2)ccc1O;Active;0.613;0.55;Yes;;312.41;4.32;46.53;1;3;9
0;COC(=O)[C@H]1CCCN1C(=O)C(=O)c1c[nH]c2ccccc12;Inactive;0.485;0.356;Yes;;300.31;1.
0;CC(C)=CCC1(O)C(=O)C(CC=C(C)C)(CC=C(C)C)C(=O)C(C(=O)C(C)C)=C1O;Inactive;0.425;0.
0;CCOC(=O)c1c(NC(C)c2ccccc2)[nH]c2ccccc12;Inactive;0.289;0.444;Yes;;308.38;4.52;5
0;CCOCCn1cc(C(=O)Nc2ccc(-n3nc(-c4cccnc4)cc3C(F)(F)F)cn2)ccc1=O;Active;0.582;0.285
0;OC1(c2cccc(COc3ccc4c(-c5ccoc5)cc(-c5nc6ccccc6s5)cc4c3)c2)CCOCC1;Active;0.807;0.
0;O=Cc1ccc(-c2c(Cc3ccccc3)nnn2CSc2ccccc2)cc1;Active;0.615;0.239;No;Outside AD: pr
0;COc1ccc(C(=O)Nc2nc3c(s2)CC2(C)CCC4C(C)C(=O)OC4C2C3C)cc1;Active;0.537;0.272;No;O
0;COc1c(CC=C(C)C)c(O)c(C(CC(C)C)c2c(O)c3c(c(C(C)=O)c2O)OC(C)(C)C=C3)c(O)c1C(C)=O;
0;O=C(O)/C=C/c1c(-c2ccc(Nc3cccc(C(F)(F)F)c3)cc2)c(-c2ccccc2)c2n1CCC2;Active;0.562
0;CCCCC(Cc1ccc(OCCc2ccccc2)c(OCCc2ccccc2)c1)C(=O)O;Active;0.686;0.5;Yes;;446.59;6.
0;CC1(C)Cc2c(c(C(=O)Nc3ccc(C(F)(F)F)cc3)cc3nc(Nc4c(F)cccc4Cl)[nH]c23)O1;Active;0.
0;CN(C)c1cccc2cc(S(=O)(=O)Nc3ccc(-c4ccc(-c5cn(Cc6ccc(C(=O)O)cc6[N+](=O)[O-])nn5)cc
0;Oc1ccc(-c2cccc3c2oc2ccccc23)c(O)c1;Inactive;0.305;0.394;Yes;;276.29;4.66;53.6;2
0;O=C(O)c1ccc(Cn2cc(-c3ccc(-c4ccccc4)cc3)nn2)c([N+](=O)[O-])c1;Inactive;0.43;0.43
0;O=C(O)c1ccc(Cn2cc(-c3ccc(-c4ccc(O)cc4)cc3)nn2)c([N+](=O)[O-])c1;Inactive;0.455;
0;O=C(Cc1ccc2ccccc2c1)c1ccc(O)cc1O;Inactive;0.29;0.324;No;Outside AD: prediction
0;COc1ccc2c(c1)c(CC(=O)O)c(C)n2C(=O)c1ccc(Cl)cc1;Inactive;0.372;0.57;Yes;;357.79;
0;C=CCN(CC=C)c1[nH]c2ccc(O)cc2c1C(=O)OCC;Inactive;0.211;0.54;Yes;;300.36;3.23;65.
0;CC(=O)Oc1ccc(/C=C/C(=O)OCCCn2cc(CCO)nn2)cc1OC(C)=O;Active;0.511;0.577;Yes;;417.
0;O=C(O)CSc1nc(OCCc2ccccc2)cc(OCCc2ccccc2)n1;Inactive;0.487;0.55;Yes;;410.5;3.9;8
0;CC(C)(C)c1ccc(CN2CCN(C(=O)CCc3cc(-c4ccc(C(F)(F)F)cc4)n(-c4ccc(Cl)cc4)n3)CC2)cc1
0;CC1C(=O)OC2C1CCC1(C)Cc3sc(NC(=O)C4CCCC4)nc3C(C)C21;Inactive;0.479;0.184;No;Outs
0;COc1ccc(-n2nc(CCC(=O)N3CCN(Cc4ccc(C(C)(C)C)cc4)CC3)cc2-c2ccc(C(F)(F)F)cc2)nn1;A
0;Cc1ccc(-c2cc(CCC(=O)Oc3ccc(C(C)C)cc3)nn2-c2ccc(Cl)nn2)cc1;Active;0.671;0.309;No
0;CCCC(Sc1nc2ccc(NC(=O)c3ccc(C(C)(C)C)cc3)cc2s1)C(=O)O;Active;0.535;0.313;No;Outs
0;COc1cc(C)c2c(c1C)CC(C(C)C(=O)NCc1cccs1)CC2;Inactive;0.356;0.526;Yes;;357.52;4.4
0;CC(=O)c1c(O)c(C(C2=C(O)CC(C)(C)CC2=O)C(C)C)c(O)c(C(C2=C(O)CC(C)(C)CC2=O)C(C)C)c
0;CC(C)C(=O)c1c(O)cc(O)c(C(C2=C(O)C(C)(C)C(=O)C(C)(C)C2=O)C(C)C)c1O;Inactive;0.32
0;O=Nc1c(-c2c(O)[nH]c3ccccc23)[nH]c2ccccc12;Inactive;0.294;0.261;No;Outside AD: p
0;C#CCCNC(=O)/C=C/c1ccc(OC(C)=O)c(OC(C)=O)c1;Active;0.517;0.383;Yes;;315.33;1.69;
0;COC(=O)CC[C@@H](NC(=O)C(=O)c1c[nH]c2ccccc12)C(=O)OC;Inactive;0.458;0.419;Yes;;3
0;O=C(O)c1ccc(N(Cc2ccoc2)S(=O)(=O)c2ccc(-c3ccccc3)cc2)cc1;Inactive;0.469;0.557;Ye
0;O=C(O)/C=C/c1ccc(O)c(O)c1;Active;0.655;0.487;Yes;;180.16;1.2;77.76;3;3;2;0.472;
0;CC(C)C(C1=C(O)CC(C)(C)CC1=O)c1c(O)c(C(=O)c2ccccc2)c(O)c(C(C2=C(O)CC(C)(C)CC2=O)
0;FC(F)(F)c1ccc(Cl)c(OCc2ccccc2-c2ccc(-c3cn(CCCc4ccccc4)nn3)cc2)c1;Inactive;0.479
0;CCCCCc1ccc(S(=O)(=O)NCCc2c(-c3ccc(OC)cc3)[nH]c3ccccc23)cc1;Inactive;0.142;0.653
0;O=C1C=C(Cc2ccc3cc(C(=O)O)ccc3c2)C(=O)C=C1O;Inactive;0.414;0.273;No;Outside AD:
0;O=C(O)/C=C/c1c(-c2ccc(O)cc2)c(-c2ccccc2)c2n1CCC2;Inactive;0.399;0.306;No;Outsid
0;OC[C@@H]1[C@@H]([C@H](O)c2ccc(O)c(O)c2)CO[C@@H]1c1ccc(O)c(O)c1;Inactive;0.357;0.
0;CC1(C)CC(=O)C2=C(C1)N(CCC(=O)O)C1=C(C(=O)CC(C)(C)C1)C2c1ccc(OCc2ccc(Cl)cc2)c(Br
0;O=C(OCc1cn(CSc2ccccc2)nn1)c1ccccc1;Inactive;0.473;0.339;No;Outside AD: predicti
0;CC(C)=CCC1=C(O)[C@](O)(CC=C(C)C)C(=O)C(C(=O)CC(C)C)=C1O;Inactive;0.402;0.251;No
0;COc1cc(O)c2c(c1)CCC21C=CC(=O)CC1;Inactive;0.305;0.28;No;Outside AD: prediction
0;Oc1ccc(Cc2ccccc2)c(O)c1;Inactive;0.472;0.301;No;Outside AD: prediction should b
0;CCCCC(Sc1ccc(OCCCOc2ccccc2)cc1)C(=O)O;Inactive;0.237;0.779;Yes;;374.5;5.27;55.7
0;COC1=CC(=O)C(O)=C(Cc2ccccc2)C1=O;Active;0.615;0.535;Yes;;244.25;1.72;63.6;1;4;3
0;O=C(O)c1ccc(Cn2cc(-c3ccc(-c4ccccc4C(=O)O)cc3)nn2)c([N+](=O)[O-])c1;Inactive;0.4

0;O=C(O)c1cccc(-c2ccc(-c3cn(Cc4ccc(C(=O)O)cc4[N+](=O)[O-])nn3)cc2)c1;Inactive;0.4
0;O=[N+]([O-])c1cccc(-c2ccc(O)cc2O)c1;Inactive;0.273;0.344;No;Outside AD: predict
0;O=C(/C=C/c1ccc(F)c(F)c1)OCCc1cnnn1CCCO;Active;0.514;0.595;Yes;;337.33;1.74;77.2
0;CS(=O)(=O)c1ccc(C2=C(c3ccccc3)C(=O)OC2)cc1;Active;0.729;0.384;Yes;;314.36;2.56;
0;O=C(O)c1ccc(Cn2cc(-c3ccc(-c4cccs4)cc3)nn2)c([N+](=O)[O-])c1;Inactive;0.405;0.37
0;O=C1C=C(Cc2ccccc2)C(=O)C=C1O;Active;0.545;0.264;No;Outside AD: prediction shoul
0;CC(C)=CCc1c(O)cc(O)c2c1O[C@H](c1ccc(O)cc1)CC2=O;Inactive;0.492;0.445;Yes;;340.3
0;COc1cc2c(c(O)c1C(=O)/C=C/c1ccc(O)cc1)C=CC(C)(C)O2;Inactive;0.302;0.409;Yes;;352.
0;CCOC(=O)c1c(CSc2ccccc2Cl)n(CC)c2c1cc(OCC)c1ccccc12;Inactive;0.295;0.51;Yes;;468.
0;CCCCC(Sc1ccc(OCCCOc2ccc(-c3ccccc3)cc2)cc1)C(=O)OCC;Active;0.723;0.639;Yes;;478.
0;CCCCC(Sc1ccc(OCCCOc2ccc(Cl)cc2OC)cc1)C(=O)O;Inactive;0.307;0.618;Yes;;438.97;5.
0;CS(=O)(=O)c1ccc(-n2nc(COc3cccc([N+](=O)[O-])c3)cc2-c2ccccc2)cc1;Inactive;0.113;
0;CCOC(=O)c1c(CSc2ccccc2Cl)n(C)c2c1cc(OC)c1ccccc12;Inactive;0.233;0.546;Yes;;439.
0;O=C1C=C(O)C(=O)C(CC2CCCCC2)=C1O;Active;0.575;0.439;Yes;;236.27;2.36;74.6;2;4;2;
0;Cc1cccc(OCCCOc2ccc(SCC(=O)O)cc2)c1C;Inactive;0.319;0.53;Yes;;346.45;4.33;55.76;
0;Cc1cccc(OCCCOc2ccc(CCC(=O)O)cc2)c1C;Inactive;0.433;0.472;Yes;;328.41;4.17;55.76
0;Cc1ccc(-c2cc(CCC(=O)Oc3ccc(C(C)C)cc3)nn2-c2ccc3ccccc3n2)cc1;Active;0.65;0.326;N
0;CC(C)=CCC1=C2O[C@@H](C(C)(C)O)C[C@@]2(O)C(=O)C(C(=O)CC(C)C)=C1O;Inactive;0.408;
0;COC1=CC(=O)C(O)=C(Cc2ccc3ccccc3c2)C1=O;Inactive;0.493;0.469;Yes;;294.31;2.88;63.
0;CC(C)C(C1=C(O)C(C)(C)C(=O)C(C)(C)C1=O)c1c(O)c(C(=O)c2ccccc2)c(O)c(C(C2=C(O)C(C)
0;COCCOc1cc(C)c2c(c1C)CC(C(C)C(=O)NC1CCCC1)CC2;Active;0.599;0.498;Yes;;373.54;4.1
0;CCCCC(Sc1ccc(OCCCOc2ccc3ncccc3c2)cc1)C(=O)O;Inactive;0.395;0.62;Yes;;425.55;5.8
0;CCCCC1=C(O)C(=O)C=C(O)C1=O;Active;0.777;0.867;Yes;;196.2;1.58;74.6;2;4;3;0.672;
0;C#CCCOC(=O)/C=C/c1ccc(F)c(F)c1;Active;0.601;0.382;Yes;;236.22;2.54;26.3;0;2;4;0.
0;Oc1ccc(-c2csc3ccccc23)c(O)c1;Inactive;0.278;0.289;No;Outside AD: prediction sho
0;Nc1ccc(-c2ccc(-c3cn(Cc4ccc(C(=O)O)cc4[N+](=O)[O-])nn3)cc2)cc1;Inactive;0.48;0.3
0;O=C(O)c1ccc(Cn2cc(-c3ccc(-c4cccc(C(=O)N5CCOCC5)c4)cc3)nn2)c([N+](=O)[O-])c1;Ina
0;CCCCC1=C(O)C(=O)C=C(OC)C1=O;Active;0.68;0.882;Yes;;210.23;1.67;63.6;1;4;4;0.717
0;CCCCC(Sc1ccc(OCCCOc2ccccc2OC)cc1)C(=O)O;Inactive;0.26;0.746;Yes;;404.53;5.28;64.
0;CCCCCCC1=C(O)C(=O)C=C(OC)C1=O;Active;0.993;1.0;Yes;;238.28;2.45;63.6;1;4;6;0.57
0;CC(=O)c1c(O)c(CC=C(C)C)c(O)c(C(CC(C)C)c2c(O)c(CC=C(C)C)c(O)c(C(C)=O)c2O)c1O;Ina
0;CCCCC(Sc1ccc(OCCCOc2cccc3c2CCC3)cc1)C(=O)OCC;Inactive;0.188;0.633;Yes;;456.65;
0;COc1cc(-c2ccc(-c3cn(Cc4ccc(C(=O)O)cc4[N+](=O)[O-])nn3)cc2)cc(OC)c1OC;Inactive;0.
0;CC(C)C(=O)c1c2c(c(O)c3c1OC1=C(C(=O)C(C)(C)C(=O)C1(C)C)C3C(C)C)C(C(C)C)C1=C(O2)C
0;COc1cccc(C(=O)N[C@H](CO)CCCCNC(=O)c2cccc(OC)c2O)c1;Inactive;0.284;0.514;Yes;;41
0;COc1cccc(C(=O)NCCCC[C@@H](CO)NC(=O)c2cccc(O)c2)c1O;Inactive;0.195;0.547;Yes;;40
0;O=C(O)c1ccc(Cn2cc(-c3ccc(-c4cccc(O)c4)cc3)nn2)c([N+](=O)[O-])c1;Inactive;0.435;
0;CCCCCc1ccc(S(=O)(=O)NCCc2nc(-c3cccs3)[nH]c2-c2ccc(OC)cc2)cc1;Inactive;0.443;0.4
0;COc1ccc(-c2ccc(-c3cn(Cc4ccc(C(=O)O)cc4[N+](=O)[O-])nn3)cc2)cc1CN1CCOCC1;Inactiv
0;CCCCC(Cc1ccc(OCCc2ccccc2)cc1OCCc1ccccc1)C(=O)O;Active;0.672;0.498;Yes;;446.59;6.
0;CC(=O)Oc1ccc(/C=C/C(=O)O)cc1OC(C)=O;Inactive;0.427;0.429;Yes;;264.23;1.64;89.9;
0;Cc1nnsc1C(=O)Nc1nc2c(s1)CC1(C)CCC3C(C)C(=O)OC3C1C2C;Active;0.533;0.191;No;Outsi
0;CC(=O)Oc1ccc(/C=C/C(=O)NCCc2cnnn2CCCO)cc1OC(C)=O;Inactive;0.388;0.643;Yes;;416.
0;CCOC(=O)c1c(CSc2ccccc2Cl)n(C)c2c1cc(O)c1ccccc12;Active;0.765;0.533;Yes;;425.94;
0;O=C1COCCN1c1ccc(Nc2nc(Cl)nc(N3CCCC3)n2)cc1;Active;0.537;0.27;No;Outside AD: pre
0;COc1cc(F)cc(OCc2cc(-c3ccccc3)n(-c3ccc(S(C)(=O)=O)cc3)n2)c1;Inactive;0.435;0.776
0;COc1ccc(-n2nc(CCC(=O)N3CCN(Cc4ccc(C(C)(C)C)cc4)CC3)cc2-c2ccc(F)cc2)nn1;Active;0.
0;CCOC(=O)C1CCN(C(=O)CCc2cc(-c3ccc(C(F)(F)F)cc3)n(-c3ccc(Cl)nn3)n2)CC1;Active;0.7
0;Cc1occc(=S)c1O;Inactive;0.417;0.263;No;Outside AD: prediction should be interpr
0;OCCc1ccc(O)c(O)c1;Active;0.642;0.37;Yes;;154.16;0.63;60.69;3;3;2;0.547;0.708;Pa
0;COc1cc(/C=C/c2cc(=O)oc3c2ccc2oc(-c4cccc(Cl)c4)cc23)cc(OC)c1;Inactive;0.145;0.78
0;CC(C)=CCC[C@@]1(C)CCC[C@]2(C)[C@@H](Cc3cc(C(=O)O)ccc3O)C(C)=CC[C@@H]12;Inactive
0;Cc1cccc(C2=N/C(=C\c3ccc(-c4cccc(Cl)c4)o3)C(=O)O2)c1;Inactive;0.478;0.348;No;Out
0;C=CCc1ccc(OC)c(-c2ccc(OC)c(CC=C)c2)c1;Inactive;0.463;0.316;No;Outside AD: predi

0;O=C([O-])/C=C(/c1ccc(F)cc1)c1ccc(Sc2cc(F)cc(C(O)(C(F)(F)F)C(F)(F)F)c2)cc1[O-].[
0;CC(C)Cc1ccc(C(C)C(=O)O)cc1;Inactive;0.255;0.454;Yes;;206.28;3.07;37.3;1;1;4;0.8
0;C/C=C/c1ccc2c(c1)[C@@H](C)[C@H](c1ccc(O)cc1)O2;Inactive;0.212;0.464;Yes;;266.34
0;COC1(c2ccc(OCc3ccc4ccccc4c3)cc2)CCOCC1;Active;0.517;0.734;Yes;;348.44;5.07;27.6
0;CCCCC/C=C\C/C=C\C/C=C\C=C\C(=O)CCCC(=O)OC;Inactive;0.13;0.6;Yes;;332.48;5.48;43.
0;CCCCC/C=C\C/C=C\C=C\C=C\C1CC1CCCCC(=O)O;Active;0.738;0.647;Yes;;330.51;6.46;37.
0;CN(C)c1ccc(/C=C2\SC(N3CCCCC3)=NC2=O)cc1;Active;0.638;0.42;Yes;;315.44;3.21;35.9
0;COc1cc(-c2cc3ccccc3c(=O)o2)cc(OC)c1OC;Inactive;0.407;0.438;Yes;;312.32;3.49;57.
0;NS(=O)(=O)c1ccc(N2C(=O)CC(C3CCCCC3=O)C2=O)cc1;Inactive;0.117;0.727;Yes;;350.4;0.
0;COc1cc(-c2cc3ccccc3c(=O)o2)ccc1O;Active;0.585;0.521;Yes;;268.27;3.17;59.67;1;4;
0;COc1ccc(NC2=NS(=O)(=O)c3cc([N+](=O)[O-])ccc32)cc1OC;Inactive;0.362;0.685;Yes;;3
0;Nc1ccccc1-c1ccc(-c2nnc(NC(=O)c3ccccc3Br)s2)cc1;Inactive;0.422;0.307;No;Outside
0;CC1(C)[C@@H](O)CC[C@]2(C)[C@H]3C(=O)C=C4[C@@H]5C[C@@](C)(C(=O)O)CC[C@]5(C)CC[C@
0;CC1(C)[C@@H](O)CC[C@]2(C)[C@H]3C(=O)C=C4[C@@H]5C[C@@](C)(CO)CC[C@]5(C)CC[C@@]4(
0;COc1ccc(-c2cc3cc(CCCO)ccc3o2)c(O)c1;Inactive;0.327;0.422;Yes;;298.34;3.74;62.83
0;CCCCC/C=C\C/C=C\C=C\C=C\C1CC1CC(=O)O;Active;0.575;0.564;Yes;;288.43;5.29;37.3;1
0;Cc1ccc(/C=C2\SC(Nc3ccc4ccccc4c3)=NC2=O)cc1;Active;0.66;0.453;Yes;;344.44;5.23;4
0;O=C1/C(=C/C=C/c2ccco2)S/C(=N\c2ccccc2)N1c1ccccc1;Inactive;0.431;0.248;No;Outsid
0;Oc1ccc(-c2ccc(-c3cn(CCCc4ccccc4)nn3)cc2)cc1;Active;0.578;0.304;No;Outside AD: p
0;c1ccc(CCCn2cc(-c3ccc(-c4ccc(Oc5ccccc5)cc4)cc3)nn2)cc1;Active;0.551;0.292;No;Out
0;COc1ccc(/C=C2\SC(N3CCCC3)=NC2=O)cc1;Active;0.685;0.575;Yes;;288.37;2.76;41.9;0;
0;O=C1N=C(Nc2ccccc2)S/C1=C\c1ccccc1;Active;0.647;0.441;Yes;;280.35;3.77;41.46;1;3
0;Cc1ccccc1CCn1cc(-c2ccc(-c3ccccc3COc3cc(C(F)(F)F)ccc3Cl)cc2)nn1;Inactive;0.484;0
0;c1ccc(CCCn2cc(-c3ccc(-c4cc5ccccc5c5ccccc45)cc3)nn2)cc1;Active;0.501;0.304;No;Ou
0;c1ccc(CCCn2cc(-c3ccc(-c4ccccc4COc4cccc5ccccc45)cc3)nn2)cc1;Active;0.552;0.369;Y
0;Cc1ccccc1CCn1cc(-c2ccc(-c3ccccc3COc3cccc4ccccc34)cc2)nn1;Inactive;0.497;0.348;N
0;CC1(C)CC[C@]2(C(=O)O)CC[C@]3(C)C(=CC[C@@H]4[C@@]5(C)C[C@H](O)[C@H](O)[C@@](C)(C
0;CC1(C)CC[C@]2(C)CC[C@]3(C)C(=CC[C@@H]4[C@@]5(C)CC[C@H](O)[C@](C)(CO)[C@@H]5CC[C
0;CCCCCCC(Sc1nc(Cl)cc(NCc2ccc3ncccc3c2)n1)C(=O)O;Active;0.685;0.533;Yes;;444.99;5.
0;CC1(C)[C@@H](O)CC[C@]2(C)[C@H]3CC=C4[C@@H]5C[C@@](C)(CO)CC[C@]5(C)CC[C@@]4(C)[C
0;O=c1/c(=C/c2cc(I)c(O)c([N+](=O)[O-])c2)sc2nc3ccccc3n12;Active;0.618;0.274;No;Ou
0;CCCCCCC(Sc1nc(Cl)cc(Nc2ccc3ncccc3c2)n1)C(=O)O;Active;0.752;0.608;Yes;;430.96;5.
0;CC1(C)CC[C@]2(C)CC[C@]3(C)C(=CC[C@@H]4[C@@]5(C)CC[C@H](O)C(C)(C)[C@@H]5CC[C@]43
0;CC1(C)CC[C@]2(C)CC[C@]3(C)C(=CC(=O)[C@@H]4[C@@]5(C)CC[C@H](O)C(C)(C)[C@@H]5CC[C
0;CC1(C)CC[C@]2(CO)CC[C@]3(C)C(=CC[C@@H]4[C@@]5(C)CC[C@H](O)C(C)(C)[C@@H]5CC[C@]4
0;C[C@H]1[C@H](C)CC[C@]2(C(=O)O)CC[C@]3(C)C(=CC[C@@H]4[C@@]5(C)C[C@@H](O)[C@H](O)
0;C[C@H]1[C@H](C)CC[C@]2(C)CC[C@]3(C)C(=CC[C@@H]4[C@@]5(C)CC[C@H](O)C(C)(C)[C@@H]
0;C[C@H]1[C@H](C)CC[C@]2(C(=O)O)CC[C@]3(C)C(=CC[C@@H]4[C@@]5(C)CC[C@H](O)C(C)(C)[
0;CC1(C)CC[C@]2(C(=O)O)CC[C@]3(C)C(=CC[C@@H]4[C@@]5(C)CC[C@H](O)[C@@](C)(CO)[C@@H
0;CC1(C)CC[C@]2(C(=O)O)CC[C@]3(C)C(=CC[C@@H]4[C@@]5(C)CC[C@H](O)[C@@](C)(C(=O)O)[
0;CC(=O)O[C@@H]1CC[C@@]2(C)[C@@H](CC[C@]3(C)[C@@H]2CC=C2[C@@H]4[C@@H](C)[C@H](C)C
0;CC1(C)CC[C@]2(C(=O)O)CC[C@]3(C)C(=CC[C@@H]4[C@@]5(C)C[C@H](O)[C@H](O)[C@@](C)(C
0;CC(=O)O[C@@H]1CC[C@@]2(C)[C@@H](CC[C@]3(C)[C@@H]2CC=C2[C@@H]4CC(C)(C)CC[C@]4(C)
0;C[C@H]1[C@H](C)CC[C@]2(C(=O)O)CC[C@]3(C)C(=CC[C@@H]4[C@@]5(C)C[C@@H](O)[C@H](O)
0;C[C@H]1[C@H](C)CC[C@]2(C)CC[C@]3(C)C(=CC[C@@H]4[C@@]5(C)CC[C@@H](O)[C@](C)(C(=O
0;CC1(C)CC[C@]2(C(=O)O)CC[C@]3(C)C(=CC[C@@H]4[C@@]5(C)C[C@@H](O)[C@H](O)C(C)(C)[C
0;COc1cc(O)ccc1-c1cc2cc(CCCO)ccc2o1;Inactive;0.339;0.41;Yes;;298.34;3.74;62.83;2;
0;CC1(C)CC[C@]2(C(=O)O)CC[C@]3(C)C(=CC[C@@H]4[C@@]5(C)C[C@@H](O)[C@H](O)[C@@](C)(
0;CC1(C)CC[C@]2(C)CC[C@]3(C)C(=CC[C@@H]4[C@@]5(C)CC[C@@H](O)[C@](C)(C(=O)O)[C@@H]
0;CC1(C)[C@@H](O)CC[C@]2(C)[C@H]3CC=C4[C@@H]5C[C@@](C)(C(=O)O)CC[C@]5(C)CC[C@@]4(
0;C[C@H]1[C@H](C)CC[C@]2(CO)CC[C@]3(C)C(=CC[C@@H]4[C@@]5(C)CC[C@H](O)C(C)(C)[C@@H
0;O=C(O)c1cccc(C(=O)Nc2ccccc2)c1;Inactive;0.172;0.486;Yes;;241.25;2.64;66.4;2;2;3
0;CCCNC(=O)c1cc(C(C)(C)C)c2c(c1)C(C)(C)CO2;Inactive;0.035;0.686;Yes;;289.42;3.79;
0;O=C(/C=C/C=C/c1ccc2c(c1)OCO2)N1CCCCC1;Inactive;0.456;0.266;No;Outside AD: predi

0;CCCCC/C=C\C/C=C\C=C\C=C\C1SC1CCCC(=O)OC;Inactive;0.468;0.511;Yes;;348.55;6.01;2[illegible]
0;COc1cc(Cl)cc(/C=C2/SC(=O)N(Cc3ccc(F)cc3)C2=O)c1O;Active;0.645;0.347;No;Outside [illegible]
0;O=C(O)c1cc(O)ccc1O;Inactive;0.14;0.323;No;Outside AD: prediction should be inte[illegible]
0;COc1ccc2c(c1)c1c(n2C(=O)c2ccc(S(=O)(=O)/N=C/N(C)C)cc2)CCN(CCCOc2cc(F)cc(C3(OC)C[illegible]

;LogP;TPSA;HBD;HBA;RotatableBonds;QED;SA_score;Lipinski;Violations
should be interpreted with caution.;326.44;4.83;40.46;2;4;1;0.737;1.553;Pass;0
02.54;5.16;63.84;0;7;6;0.379;2.005;Fail;1
Yes;;472.09;8.01;42.23;1;3;7;0.355;2.244;Fail;1
;3;5;0.672;1.467;Pass;0
.941;Pass;0
;0.983;0.696;Yes;;460.54;3.3;112.98;2;6;5;0.567;2.421;Pass;0
42;6.35;81.01;1;6;8;0.286;2.607;Fail;2
tive;0.978;0.799;Yes;;658.8;8.76;73.58;1;6;12;0.142;3.918;Fail;2
46.55;6.3;81.01;1;5;9;0.308;2.593;Fail;1
.55;6.3;81.01;1;5;9;0.308;2.593;Fail;1
;5;5;0.673;1.725;Pass;0
3.97;49.69;0;6;5;0.647;2.107;Pass;0
592;Yes;;456.67;6.55;69.92;3;4;11;0.33;2.413;Fail;1
19.51;4.34;111.53;2;6;5;0.598;2.139;Pass;0
es;;442.64;6.24;69.92;3;4;11;0.355;2.385;Fail;1
4.65;61.8;2;3;3;0.574;1.763;Pass;0
s;;360.35;4.85;84.71;1;4;4;0.416;1.921;Pass;0
3;81.01;1;5;10;0.382;2.385;Fail;1
421.53;4.43;100.63;2;6;6;0.573;2.443;Pass;0
96;Yes;;426.6;7.05;66.76;2;3;10;0.311;2.253;Fail;1
ctive;1.0;0.871;Yes;;675.25;9.27;73.58;1;6;12;0.14;3.951;Fail;2
475.03;7.11;55.12;1;3;9;0.281;2.65;Fail;1
9;6.69;38.66;0;3;4;0.354;2.215;Fail;1
4;5.8;98.36;2;7;11;0.278;2.613;Fail;1
.664;1.182;Pass;0
;4;5;0.853;1.473;Pass;0
.908;0.577;Yes;;483.69;7.11;70.0;2;4;12;0.245;2.567;Fail;1
56;38.66;0;3;3;0.559;1.86;Fail;1
.49;3.73;66.76;0;6;6;0.568;2.243;Pass;0
35;0.658;Yes;;419.54;5.48;44.37;2;2;4;0.689;2.639;Fail;1
;0.668;Yes;;404.53;5.54;32.34;1;2;4;0.725;2.609;Fail;1
e;0.955;0.728;Yes;;545.76;8.29;67.79;2;4;13;0.196;2.992;Fail;2
5.98;38.66;0;3;5;0.49;2.112;Fail;1
6.19;38.66;0;3;4;0.497;1.984;Fail;1
8.66;0;3;5;0.691;1.816;Fail;1
;Yes;;460.7;7.9;66.76;2;3;13;0.314;2.621;Fail;1
38.66;0;3;3;0.633;1.791;Pass;0
;58.89;1;4;3;0.692;1.823;Pass;0
94.25;2;5;4;0.662;1.669;Pass;0
1;4;4;0.693;1.623;Pass;0
38.66;0;3;3;0.783;1.56;Pass;0
OC;Active;0.816;0.453;Yes;;620.83;8.51;86.61;2;7;13;0.217;3.342;Fail;2
.831;0.487;Yes;;528.69;5.99;113.29;3;6;15;0.145;2.957;Fail;2
.19;38.66;0;3;4;0.497;1.984;Fail;1
;Yes;;442.64;6.24;69.92;3;4;11;0.355;2.385;Fail;1
49.53;7.33;47.89;0;4;6;0.289;2.499;Fail;1
.66;0;3;3;0.742;1.649;Pass;0
;2;5;4;0.665;1.661;Pass;0
839;1.317;Pass;0
.914;1.325;Pass;0
7;3.75;57.53;0;5;5;0.665;2.083;Pass;0
5;2;5;4;0.662;1.669;Pass;0
.66;0;3;3;0.783;1.56;Pass;0

8;31.35;0;4;7;0.378;2.279;Fail;1
08;0.475;Yes;;525.77;6.91;73.16;3;5;13;0.244;2.952;Fail;2
0.498;Yes;;485.71;5.99;73.16;3;5;13;0.299;2.671;Fail;1
9.87;4.18;67.59;1;5;4;0.399;1.82;Pass;0
ive;0.978;0.791;Yes;;545.76;8.29;67.79;2;4;13;0.196;2.992;Fail;2
tive;0.963;0.765;Yes;;559.79;8.6;67.79;2;4;13;0.19;3.02;Fail;2
1;0.462;Yes;;527.75;5.76;82.39;3;6;13;0.283;2.955;Fail;2
2;5;4;0.667;1.633;Pass;0
3;5.49;62.32;2;3;3;0.476;1.819;Fail;1
.48;4.86;107.59;2;8;10;0.337;2.489;Pass;0
874;0.644;Yes;;493.73;7.98;49.77;1;3;10;0.265;2.587;Fail;1
;;452.55;6.29;55.76;1;4;6;0.35;2.505;Fail;1
56;38.66;0;3;3;0.559;1.86;Fail;1
02;2;4;7;0.454;2.009;Pass;0
9;6.69;38.66;0;3;4;0.354;2.215;Fail;1
.551;Yes;;472.61;5.91;67.76;1;4;5;0.549;2.845;Fail;1
37.52;6.7;47.89;0;4;5;0.326;2.375;Fail;1
74;59.06;1;4;5;0.524;1.833;Pass;0
1;5.86;44.37;2;2;5;0.42;2.351;Fail;1
.711;Yes;;440.62;7.36;66.76;2;3;10;0.295;2.281;Fail;1
;;428.61;5.93;69.92;3;4;11;0.382;2.357;Fail;1
.47;4.99;55.73;0;4;5;0.448;2.099;Pass;0
c1OC(C)=O;Active;0.545;0.543;Yes;;691.73;4.5;171.44;0;14;13;0.106;3.483;Fail;2
0.98;0.772;Yes;;597.18;8.68;64.35;1;5;9;0.184;3.294;Fail;2
.93;2;4;5;0.654;1.513;Pass;0
46.5;0;3;3;0.627;1.543;Pass;0
22;Yes;;456.62;5.74;86.99;3;5;12;0.276;2.513;Fail;1
Yes;;428.61;7.13;66.76;2;3;11;0.368;2.357;Fail;1
6.06;38.66;0;3;3;0.396;2.091;Fail;1
;94.25;2;5;4;0.662;1.669;Pass;0
2;6.11;62.32;2;4;5;0.31;2.111;Fail;1
8;0.477;Yes;;511.75;6.52;73.16;3;5;13;0.263;2.923;Fail;2
.2;69.92;3;4;8;0.608;1.921;Pass;0
Yes;;442.64;7.22;55.76;1;4;11;0.29;2.385;Fail;1
0.511;Yes;;470.65;6.05;86.99;3;5;12;0.256;2.541;Fail;1
0.468;Yes;;456.58;4.53;66.48;1;4;6;0.694;2.513;Pass;0
9.89;5.68;42.23;1;2;4;0.633;1.96;Fail;1
6;0;3;3;0.783;1.56;Pass;0
;4.46;78.9;0;6;12;0.169;2.201;Pass;0
.564;Yes;;440.62;7.36;66.76;2;3;10;0.295;2.281;Fail;1
;4;15;0.265;2.429;Fail;1
8.5;4.51;62.58;0;6;7;0.508;2.377;Pass;0
2;4;0.835;1.363;Fail;1
;0;3;3;0.807;1.527;Pass;0
.47;0;5;5;0.681;2.037;Pass;0
;0.798;0.449;Yes;;522.68;6.53;96.97;2;6;12;0.187;2.845;Fail;2
ve;0.8;0.507;Yes;;576.77;8.71;66.38;1;6;12;0.255;3.154;Fail;2
cc2)OC1;Active;0.748;0.44;Yes;;591.75;8.09;111.29;2;7;12;0.146;3.183;Fail;2
8.66;0;3;3;0.802;1.519;Pass;0
5;47.89;0;4;6;0.715;1.907;Pass;0
;74.22;1;6;8;0.577;1.917;Pass;0
46.36;6.8;62.32;2;4;5;0.261;2.193;Fail;1
;2;4;5;0.646;1.781;Pass;0
.87;70.14;1;5;5;0.714;2.087;Pass;0

;5.28;51.46;1;5;5;0.481;1.867;Fail;1
;3;3;0.675;1.493;Pass;0
;14;0.303;2.301;Fail;1
1;Yes;;494.54;5.48;74.22;1;6;5;0.371;2.889;Fail;1
;;393.42;4.51;102.2;1;6;6;0.287;1.987;Pass;0
4.22;1;6;8;0.577;1.917;Pass;0
1;6.15;62.32;2;4;5;0.305;2.124;Fail;1
8.34;6.02;48.0;0;4;6;0.453;2.317;Fail;1
42.54;5.33;67.76;1;4;7;0.503;2.385;Fail;1
n should be interpreted with caution.;280.28;2.78;52.6;0;4;3;0.811;1.461;Pass;0
.52;6.11;62.32;2;4;5;0.31;2.111;Fail;1
74.35;3;4;3;0.637;1.533;Pass;0
.564;Yes;;440.62;7.36;66.76;2;3;10;0.295;2.281;Fail;1
41.57;1;2;3;0.581;1.731;Pass;0
5;74.35;3;4;4;0.618;1.662;Pass;0
765;0.805;Yes;;580.57;8.63;55.76;1;3;13;0.162;3.061;Fail;2
on should be interpreted with caution.;264.28;2.9;54.37;1;3;2;0.848;1.329;Pass;0
0.81;0.462;Yes;;540.79;5.68;76.4;3;6;13;0.28;2.982;Fail;2
02;2;4;4;0.655;1.681;Pass;0
.89;0;4;4;0.803;1.651;Pass;0
;0.503;1.469;Pass;0
8.62;7.06;109.36;3;7;14;0.161;2.997;Fail;1
s;;509.13;6.7;35.94;1;4;10;0.309;2.818;Fail;2
;Yes;;462.63;4.09;110.38;5;6;12;0.299;2.525;Pass;0
;2;4;5;0.653;1.753;Pass;0
39;1.405;Pass;0
;5;4;0.667;1.633;Pass;0
es;;504.59;8.29;55.76;1;3;12;0.296;2.809;Fail;2
.06;58.43;2;5;5;0.416;2.333;Fail;1
;481.02;6.93;98.9;2;6;11;0.133;2.662;Fail;1
.61;Yes;;513.59;7.48;66.84;1;3;9;0.222;2.927;Fail;2
58;0.679;Yes;;454.65;7.67;66.76;2;3;10;0.281;2.309;Fail;1
prediction should be interpreted with caution.;359.45;5.07;50.94;1;5;5;0.506;2.01[illegible]
77.76;3;4;6;0.542;1.409;Pass;0
;3.83;85.02;2;4;5;0.596;1.889;Pass;0
;Yes;;414.59;6.72;66.76;2;3;9;0.44;2.129;Fail;1
es;;412.57;6.66;66.76;2;3;10;0.333;2.225;Fail;1
29;78.9;0;6;9;0.222;1.817;Pass;0
386.53;6.1;66.76;2;3;9;0.503;2.073;Fail;1
ve;0.787;0.433;Yes;;552.71;6.51;106.2;2;7;13;0.166;3.005;Fail;2
es;;422.49;3.99;87.74;2;4;6;0.492;2.245;Pass;0
3.72;3;4;0;0.385;1.512;Pass;0
.97;7.1;75.11;2;5;6;0.156;2.544;Fail;1
.83;0.747;Yes;;634.6;7.25;100.0;1;8;16;0.099;3.469;Fail;2
;500.06;7.37;84.34;2;6;12;0.116;2.8;Fail;2
401.4;1.22;154.52;3;7;5;0.64;1.903;Pass;0
3;5;0.646;1.437;Pass;0
;5.05;59.06;1;4;5;0.498;1.861;Fail;1
44;4.75;68.29;1;5;6;0.482;1.993;Pass;0
6.83;63.25;2;4;5;0.355;2.189;Fail;1
4;54.45;1;3;3;0.729;1.769;Pass;0
4;2;0.764;1.381;Pass;0
5.95;64.99;1;4;14;0.33;2.897;Fail;1
Outside AD: prediction should be interpreted with caution.;461.53;5.32;80.32;2;5;[illegible]

.42;77.0;1;7;11;0.117;2.618;Fail;1
.46;81.54;1;6;14;0.25;2.933;Fail;1
7.06;6.82;88.0;2;7;11;0.136;2.654;Fail;1
;0.383;0.463;Yes;;561.62;6.28;111.38;1;7;8;0.233;3.123;Fail;2
OC(C)=O;Active;0.526;0.562;Yes;;663.64;3.43;188.51;0;15;15;0.1;3.427;Fail;2
.47;2.99;76.45;0;5;4;0.502;1.993;Pass;0
;3.69;75.99;2;5;6;0.767;1.725;Pass;0
4;41.8;1;6;4;0.713;2.165;Pass;0
4;16;0.186;2.557;Fail;1
2;Yes;;394.14;3.46;124.94;0;7;5;0.331;1.688;Pass;0
;393.44;4.35;75.71;1;4;4;0.559;1.987;Pass;0
68;1;4;3;0.697;1.521;Pass;0
tive;0.831;0.45;Yes;;536.71;6.84;96.97;2;6;12;0.175;2.873;Fail;2
.67;62.3;1;5;6;0.49;1.983;Pass;0
.36;6.8;62.32;2;4;5;0.261;2.193;Fail;1
O)C(C)(C)C(=O)C(C)(C)C2=O)c1O;Active;0.708;0.689;Yes;;710.91;8.32;186.5;5;10;11;0.
OC(=O)/C=C/c3ccc(O)c(O)c3)nn2)Cn2cc(COC(=O)/C=C/c3ccc(O)c(O)c3)nn2)nn1;Active;0.8[illegible]
2;46.53;1;3;1;0.621;1.105;Pass;0
;Yes;;443.59;6.83;57.61;1;2;5;0.47;1.987;Fail;1
.57;6.59;55.76;1;3;13;0.302;2.789;Fail;1
748;0.643;Yes;;504.62;6.39;93.06;2;6;12;0.198;2.609;Fail;2
1;7.06;75.11;2;5;11;0.139;2.612;Fail;1
49;62.32;2;3;5;0.671;1.788;Pass;0
rpreted with caution.;134.14;1.27;48.91;2;2;0;0.57;0.668;Pass;0
22.51;3.29;68.2;0;5;5;0.692;2.145;Pass;0
4;0.823;Yes;;537.61;4.56;105.67;1;7;8;0.353;2.875;Fail;1
ould be interpreted with caution.;229.2;2.19;41.62;1;3;1;0.802;0.958;Pass;0
4.1;67.76;1;4;5;0.809;1.833;Pass;0
67;1;4;3;0.715;1.533;Pass;0
c(COC(=O)/C=C/c3ccc(O)c(O)c3)nn2)nn1;Active;0.804;0.508;Yes;;865.81;2.34;312.64;7;
.38;45.22;1;2;2;0.491;1.909;Fail;1
.67;8.04;55.76;1;3;13;0.305;2.813;Fail;1
97;51.46;1;5;5;0.51;1.839;Pass;0
;0.263;No;Outside AD: prediction should be interpreted with caution.;512.36;6.88;[illegible]
tion should be interpreted with caution.;290.27;3.56;67.51;1;4;1;0.696;1.481;Pass;
51.22;1;3;4;0.913;1.677;Pass;0
24;51.22;1;4;6;0.739;1.973;Fail;1
7;6.59;55.76;1;3;13;0.302;2.789;Fail;1
5.66;51.46;1;5;5;0.436;1.921;Fail;1
51.22;1;4;6;0.782;1.945;Pass;0
.3;57.65;0;6;13;0.172;2.807;Fail;1
;Yes;;481.02;6.93;98.9;2;6;11;0.133;2.662;Fail;1
0.305;No;Outside AD: prediction should be interpreted with caution.;469.56;5.7;76.
3;5.37;51.46;1;5;5;0.455;1.919;Fail;1
02;Yes;;442.64;7.44;66.76;2;3;11;0.35;2.385;Fail;1
409.53;3.56;72.91;0;6;4;0.772;1.819;Pass;0
o;Outside AD: prediction should be interpreted with caution.;499.42;7.04;62.66;1;[illegible]
5;0.503;1.469;Pass;0
diction should be interpreted with caution.;359.45;5.07;50.94;1;5;5;0.506;2.019;F[illegible]
ide AD: prediction should be interpreted with caution.;446.64;5.4;44.53;1;5;7;0.5[illegible]
9;75.99;2;5;3;0.673;1.521;Pass;0
51.47;1;3;5;0.895;1.783;Pass;0
OC)c1OC;Inactive;0.471;0.243;No;Outside AD: prediction should be interpreted with
5.43;5.25;60.69;1;6;6;0.432;2.051;Fail;1

.36;55.76;1;3;14;0.313;2.893;Fail;1
7;51.22;1;3;6;0.794;1.933;Pass;0
7;6.59;55.76;1;3;13;0.302;2.789;Fail;1
.738;0.717;Yes;;613.67;7.23;77.46;1;6;15;0.181;3.527;Fail;2
;4.92;47.89;0;4;4;0.608;1.951;Pass;0
5;4.93;66.76;2;3;7;0.543;1.789;Pass;0
;78.9;0;6;8;0.237;1.689;Pass;0
O)C(C)(C)C(=O)C1(C)C)[C@@H]2C(C)C;Inactive;0.243;0.516;Yes;;650.81;7.28;155.27;3;9
ive;0.901;0.628;Yes;;521.79;8.59;49.77;1;3;10;0.248;2.644;Fail;2
;5;0.806;1.465;Pass;0
tive;0.46;0.621;Yes;;459.59;5.29;81.67;3;3;6;0.549;2.919;Fail;1
867;1.189;Pass;0
;2;4;0.745;1.373;Fail;1
8;0.305;No;Outside AD: prediction should be interpreted with caution.;423.56;4.93;
3;1;1;5;0.683;1.497;Fail;1
0.821;1.089;Pass;0
es;;457.55;5.76;74.68;1;3;7;0.383;2.415;Fail;1
c1OC;Active;0.757;0.421;Yes;;662.86;9.24;92.68;1;7;17;0.132;3.826;Fail;2
(CO)CO;Active;0.823;0.417;Yes;;652.97;5.42;82.88;3;8;15;0.217;3.606;Fail;2
76;2;3;4;0.829;1.353;Pass;0
95;83.03;1;6;5;0.736;2.061;Pass;0
.027;Pass;0
2;0.79;1.121;Pass;0
0.89;1.307;Pass;0
.779;1.206;Pass;0
2.65;7.29;66.76;2;3;13;0.343;2.565;Fail;1
.47;20.23;1;1;2;0.628;1.245;Fail;1
8;0.414;Yes;;417.53;5.92;46.06;2;2;4;0.544;2.635;Fail;1
2;3;0.675;1.241;Pass;0
1;2;0.626;1.349;Fail;1
4;0.855;1.345;Pass;0
;56.51;1;5;5;0.727;2.065;Pass;0
;0.825;0.583;Yes;;498.66;6.92;82.06;1;6;11;0.202;2.497;Fail;1
;0.889;0.649;Yes;;507.76;8.28;49.77;1;3;10;0.256;2.616;Fail;2
.08;54.46;1;5;7;0.76;2.103;Pass;0
898;1.201;Pass;0
1;2;0.79;1.121;Pass;0
6;Yes;;456.67;7.52;44.76;0;4;12;0.241;2.513;Fail;1
1;4;16;0.251;2.557;Fail;1
;5;7;0.439;1.489;Pass;0
on should be interpreted with caution.;298.38;3.99;49.69;2;3;4;0.897;1.597;Pass;0
tion should be interpreted with caution.;280.37;4.83;29.46;1;2;5;0.795;1.461;Pass;
64;77.0;1;7;9;0.204;2.362;Fail;1
;0.568;Yes;;501.56;5.22;93.14;1;5;7;0.373;2.703;Fail;2
;0.492;1.461;Pass;0
.23;1;2;2;0.697;1.229;Fail;1
5;5.82;77.0;1;7;7;0.164;2.402;Fail;1
2;74.35;3;4;5;0.614;1.749;Pass;0
8;31.35;0;4;7;0.378;2.279;Fail;1
)(C)C(=O)C1(C)C)C2C(C)C;Inactive;0.128;0.571;Yes;;678.86;8.21;155.27;3;9;9;0.133;3
;0.286;No;Outside AD: prediction should be interpreted with caution.;483.59;5.84;7
;437.5;5.91;66.84;1;3;7;0.39;2.375;Fail;1
33;3.21;118.43;0;6;8;0.138;1.663;Pass;0
AD: prediction should be interpreted with caution.;354.39;3.53;90.92;0;7;6;0.382;

6;0.811;1.569;Pass;0
;6.28;77.0;1;7;12;0.109;2.746;Fail;1
;1.117;Pass;0
48;0.565;Yes;;572.15;8.03;70.9;2;5;7;0.235;3.044;Fail;2
29.63;6.25;27.3;2;2;5;0.469;2.159;Fail;1
Yes;;481.02;6.93;98.9;2;6;11;0.133;2.662;Fail;1
1;2;6;0.788;1.561;Pass;0
37.56;5.83;74.68;1;3;10;0.405;2.475;Fail;1
572;Yes;;575.96;7.27;93.57;2;7;14;0.08;2.952;Fail;2
.448;Yes;;532.09;7.11;70.9;2;5;7;0.266;2.764;Fail;2
;0.724;0.296;No;Outside AD: prediction should be interpreted with caution.;561.11;
707;0.953;Pass;0
;4;17;0.211;2.685;Fail;1
81;55.76;1;3;11;0.394;2.265;Fail;1
9;0.641;Yes;;460.57;5.32;132.13;4;7;8;0.234;2.121;Fail;1
33.12;1;2;2;0.788;1.219;Fail;1
.46;74.2;0;7;11;0.107;2.62;Fail;1
rediction should be interpreted with caution.;354.46;4.75;56.73;1;4;5;0.526;2.009;
diction should be interpreted with caution.;356.18;3.42;73.72;3;4;0;0.385;1.512;Pa
49;62.32;2;3;5;0.671;1.788;Pass;0
es;;457.53;7.05;57.61;1;2;6;0.297;2.515;Fail;1
s;;404.42;3.5;155.52;6;8;6;0.4;1.809;Fail;1
should be interpreted with caution.;270.37;4.67;40.46;2;2;5;0.828;1.441;Pass;0
;3.51;77.98;1;4;3;0.754;1.663;Pass;0
5.89;64.11;1;6;7;0.219;2.156;Fail;1
.97;51.46;1;5;5;0.51;1.839;Pass;0
9;6.25;75.11;2;5;9;0.132;2.628;Fail;1
78.9;0;6;6;0.263;1.433;Pass;0
.98;54.69;1;6;4;0.745;2.155;Pass;0
5;0.286;No;Outside AD: prediction should be interpreted with caution.;453.56;6.03;
6;2;4;5;0.595;1.253;Pass;0
7.89;0;3;3;0.815;1.487;Pass;0
ctive;0.49;0.314;No;Outside AD: prediction should be interpreted with caution.;565
4.54;6.51;44.12;0;4;7;0.251;2.349;Fail;1
o;Outside AD: prediction should be interpreted with caution.;454.55;4.38;86.75;1;7
o;Outside AD: prediction should be interpreted with caution.;420.51;6.61;54.26;2;3
;5.2;49.83;1;2;13;0.22;2.137;Fail;1
75.11;2;5;10;0.217;2.216;Fail;1
;55.76;1;3;12;0.485;2.369;Fail;1
prediction should be interpreted with caution.;320.34;1.92;100.9;2;6;9;0.312;1.74
D: prediction should be interpreted with caution.;374.34;5.26;59.15;1;5;3;0.497;1.
0;Inactive;0.489;0.26;No;Outside AD: prediction should be interpreted with caution
tion should be interpreted with caution.;280.37;4.83;29.46;1;2;5;0.795;1.461;Pass;
5;Yes;;494.57;6.0;87.57;1;4;7;0.3;2.689;Fail;1
ion should be interpreted with caution.;324.43;5.38;40.46;2;4;1;0.493;1.549;Fail;1
2;3;3;0.505;1.247;Pass;0
o;Outside AD: prediction should be interpreted with caution.;419.52;6.46;42.23;1;2
47.89;0;4;6;0.715;1.907;Pass;0
27;1.149;Pass;0
25;0.269;No;Outside AD: prediction should be interpreted with caution.;467.59;6.17
rediction should be interpreted with caution.;322.4;4.74;35.53;0;3;7;0.419;1.745;F
36;60.69;1;6;6;0.515;1.943;Pass;0
43;0;5;4;0.67;1.901;Pass;0
99.88;4.03;68.8;2;5;4;0.648;2.0;Pass;0

diction should be interpreted with caution.;289.33;2.39;81.0;3;4;3;0.453;1.279;Pas
35;3;4;4;0.64;1.593;Pass;0
ould be interpreted with caution.;250.3;3.84;40.46;2;2;2;0.725;1.301;Pass;0
active;0.374;0.3;No;Outside AD: prediction should be interpreted with caution.;554
;75.11;2;5;8;0.227;2.204;Fail;1
;Inactive;0.426;0.33;No;Outside AD: prediction should be interpreted with caution.
;;450.34;5.4;74.68;1;3;6;0.546;2.101;Fail;1
75;7.69;81.54;1;6;14;0.157;3.014;Fail;2
s;;445.56;4.58;64.43;0;5;7;0.499;2.391;Pass;0
4;3;0.783;1.467;Pass;0
1.46;1;5;5;0.553;1.783;Pass;0
67;0.352;No;Outside AD: prediction should be interpreted with caution.;524.68;6.25
o;Outside AD: prediction should be interpreted with caution.;437.56;7.02;42.23;1;3
o;Outside AD: prediction should be interpreted with caution.;433.55;6.65;42.23;1;2
O)c1O;Inactive;0.436;0.262;No;Outside AD: prediction should be interpreted with ca
;5.86;75.11;2;5;8;0.199;2.5;Fail;1
.66;75.11;2;5;8;0.281;2.232;Fail;1
5;5.2;74.6;2;4;8;0.469;1.689;Fail;1
;2;0.649;1.141;Pass;0
0;1;7;6;0.301;1.95;Pass;0
ediction should be interpreted with caution.;356.18;3.42;73.72;3;4;0;0.385;1.512;P
;0.777;0.385;Yes;;559.13;6.91;41.37;0;4;7;0.247;2.818;Fail;2
.524;0.243;No;Outside AD: prediction should be interpreted with caution.;535.35;7.
utside AD: prediction should be interpreted with caution.;375.43;3.86;110.59;0;6;6
;465.57;5.95;83.91;1;4;8;0.444;2.531;Fail;1
7;20.23;1;2;2;0.627;1.298;Fail;1
2;58.89;1;4;3;0.824;1.62;Pass;0
)C)c1O;Inactive;0.152;0.631;Yes;;612.8;8.45;152.36;5;8;11;0.123;2.926;Fail;2
317;No;Outside AD: prediction should be interpreted with caution.;535.33;7.0;104.7
(C)CC[C@]4(C)CC[C@]23C)[C@@]1(C)C(=O)O;Active;0.66;0.286;No;Outside AD: prediction
;0.365;Yes;;520.72;6.42;41.37;0;4;7;0.282;2.741;Fail;2
on should be interpreted with caution.;277.33;3.92;71.84;3;3;3;0.679;1.455;Pass;0
.666;0.289;No;Outside AD: prediction should be interpreted with caution.;557.14;5.
)CC(C)(C)CC2=O)C(C)C)c1O;Inactive;0.128;0.468;Yes;;708.93;9.63;172.59;6;9;9;0.146;
O)cc1C;Inactive;0.497;0.269;No;Outside AD: prediction should be interpreted with c
e;0.584;0.272;No;Outside AD: prediction should be interpreted with caution.;587.75
e interpreted with caution.;186.21;2.76;40.46;2;2;1;0.718;0.872;Pass;0
ve;0.733;0.282;No;Outside AD: prediction should be interpreted with caution.;575.7
9;Pass;0
o;Outside AD: prediction should be interpreted with caution.;451.59;7.16;42.23;1;3
ive;0.758;0.38;Yes;;574.69;7.13;41.37;0;4;7;0.236;2.849;Fail;2
;60.69;1;6;6;0.515;1.943;Pass;0
6;1.309;Pass;0
.718;0.381;Yes;;555.17;7.08;41.37;0;4;7;0.245;2.81;Fail;2
;Active;0.724;0.311;No;Outside AD: prediction should be interpreted with caution.;
36;3.37;102.03;1;6;4;0.512;1.713;Pass;0
.21;38.33;1;3;7;0.71;2.071;Fail;1
;1;1;13;0.254;2.133;Fail;1
541;1.533;Pass;0
c1;Active;0.715;0.297;No;Outside AD: prediction should be interpreted with caution
utside AD: prediction should be interpreted with caution.;439.58;4.72;80.32;2;5;3;
C(=O)O)[C@@H]5CC[C@]43C)[C@H]12;Active;0.563;0.257;No;Outside AD: prediction shoul
9;6.08;75.11;2;5;6;0.215;2.28;Fail;1
3;3.38;68.8;2;5;4;0.694;1.931;Pass;0

2;0;4;4;0.62;1.897;Fail;1
ediction should be interpreted with caution.;311.73;3.31;73.72;3;4;0;0.397;1.423;F
;449.57;5.83;74.68;1;3;7;0.493;2.399;Fail;1
0.568;Yes;;487.53;5.18;93.14;1;5;7;0.386;2.675;Fail;1
21;93.06;2;6;10;0.622;2.145;Pass;0
63;51.46;1;5;5;0.52;1.855;Pass;0
1;1;6;6;0.362;1.928;Pass;0
.679;0.295;No;Outside AD: prediction should be interpreted with caution.;552.72;5.
iction should be interpreted with caution.;295.27;2.79;73.72;3;4;0;0.397;1.391;Pas
s;;443.52;5.45;74.68;1;3;7;0.412;2.387;Fail;1
should be interpreted with caution.;278.38;2.2;41.57;1;3;2;0.842;1.157;Pass;0
64.68;1;4;3;0.69;1.549;Pass;0
4.14;51.1;1;3;4;0.767;1.905;Pass;0
2;4;4;0.479;1.32;Pass;0
Yes;;473.55;5.88;83.91;1;4;8;0.345;2.547;Fail;1
;0.645;0.282;No;Outside AD: prediction should be interpreted with caution.;571.77;
1;38.66;0;3;3;0.633;1.791;Pass;0
c(C(C)=O)c2O)c1O;Active;0.52;0.334;No;Outside AD: prediction should be interpretec
92;1.156;Pass;0
01;51.46;1;5;5;0.503;1.852;Fail;1
ould be interpreted with caution.;266.34;2.33;32.34;1;2;1;0.792;1.033;Pass;0
2;5;5;0.494;1.772;Pass;0
38.33;1;2;3;0.916;1.335;Pass;0
;1.149;Pass;0
06;No;Outside AD: prediction should be interpreted with caution.;496.61;5.13;77.32
.51;5.05;74.68;1;3;8;0.565;2.219;Fail;1
tside AD: prediction should be interpreted with caution.;454.59;4.59;77.52;1;6;4;0
3.6;1;4;6;0.6;1.381;Pass;0
0.801;Pass;0
D: prediction should be interpreted with caution.;329.4;4.87;42.23;1;2;4;0.694;1.8
.43;5.37;51.46;1;5;5;0.455;1.919;Fail;1
66.02;1;5;1;0.732;1.995;Pass;0
n should be interpreted with caution.;266.34;4.22;40.46;2;2;5;0.795;1.433;Pass;0
s;;449.55;5.51;74.68;1;4;7;0.404;2.399;Fail;1
2.96;6.49;88.0;2;6;6;0.166;2.546;Fail;1
.25;74.68;1;3;11;0.52;2.307;Fail;1
O;Inactive;0.443;0.248;No;Outside AD: prediction should be interpreted with cautic
 AD: prediction should be interpreted with caution.;350.41;4.66;52.6;0;4;7;0.414;1
o;Outside AD: prediction should be interpreted with caution.;405.5;6.53;42.23;1;2;
rediction should be interpreted with caution.;245.19;1.57;97.51;1;5;2;0.484;1.09;F
56.36;3.37;102.03;1;6;4;0.512;1.713;Pass;0
6.36;55.76;1;3;14;0.313;2.893;Fail;1
utside AD: prediction should be interpreted with caution.;489.35;4.51;112.57;3;4;6
37.3;1;1;3;2;0.765;1.233;Fail;1
64.99;1;5;13;0.362;2.509;Fail;1
;;431.51;5.24;74.68;1;3;6;0.453;2.263;Fail;1
.76;1;4;12;0.38;2.377;Fail;1
55.76;1;4;12;0.38;2.377;Fail;1
;468.65;4.71;48.47;1;4;10;0.346;2.737;Pass;0
2;0.692;1.056;Pass;0
4.35;3;4;4;0.634;1.621;Pass;0
9;51.46;1;5;5;0.539;1.819;Pass;0
;Inactive;0.417;0.305;No;Outside AD: prediction should be interpreted with caution
.96;6.56;75.11;2;5;6;0.197;2.3;Fail;1

1;0.408;Yes;;444.4;3.97;148.45;2;7;7;0.321;2.389;Pass;0
;No;Outside AD: prediction should be interpreted with caution.;454.96;7.26;54.26;
55.76;1;4;12;0.38;2.377;Fail;1
e AD: prediction should be interpreted with caution.;344.41;4.45;68.25;2;3;4;0.54
0;Inactive;0.475;0.245;No;Outside AD: prediction should be interpreted with cauti
0;Inactive;0.459;0.26;No;Outside AD: prediction should be interpreted with cautio
;0.702;1.925;Pass;0
.51;79.47;1;4;3;0.529;1.501;Pass;0
245;No;Outside AD: prediction should be interpreted with caution.;416.56;4.96;91.
4.12;2;3;5;0.681;1.717;Pass;0
;No;Outside AD: prediction should be interpreted with caution.;498.47;3.8;103.93;
679;Yes;;533.65;7.95;64.72;1;6;6;0.235;3.067;Fail;2
ediction should be interpreted with caution.;385.49;5.1;47.78;0;5;7;0.328;2.271;F
utside AD: prediction should be interpreted with caution.;440.57;4.66;77.52;1;6;3;
Inactive;0.475;0.291;No;Outside AD: prediction should be interpreted with caution.
;0.272;No;Outside AD: prediction should be interpreted with caution.;488.51;7.63;
.36;55.76;1;3;14;0.313;2.893;Fail;1
547;0.242;No;Outside AD: prediction should be interpreted with caution.;518.9;7.0
4)cc3)ccc2c1;Inactive;0.487;0.334;No;Outside AD: prediction should be interpreted
;3;1;0.531;1.453;Pass;0
2;Yes;;400.39;4.27;111.15;1;6;6;0.382;2.201;Pass;0
0.404;Yes;;416.39;3.97;131.38;2;7;6;0.359;2.233;Pass;0
should be interpreted with caution.;278.31;3.68;57.53;2;3;3;0.719;1.457;Pass;0
3.93;68.53;1;4;4;0.768;1.716;Pass;0
56;2;4;7;0.608;1.701;Pass;0
42;1.31;129.84;1;10;10;0.263;2.235;Pass;0
1.54;1;6;11;0.379;2.521;Pass;0
;Active;0.786;0.414;Yes;;609.14;7.79;41.37;0;4;7;0.215;2.918;Fail;2
ide AD: prediction should be interpreted with caution.;402.56;4.53;68.29;1;5;2;0.
ctive;0.712;0.31;No;Outside AD: prediction should be interpreted with caution.;60
;Outside AD: prediction should be interpreted with caution.;460.97;5.95;69.9;0;6;
ide AD: prediction should be interpreted with caution.;442.61;6.19;79.29;2;5;7;0.
3;38.33;1;3;5;0.859;1.815;Pass;0
10;Inactive;0.152;0.489;Yes;;556.7;6.89;152.36;5;8;7;0.228;2.413;Fail;2
1;0.576;Yes;;432.51;4.4;132.13;4;7;5;0.4;1.765;Pass;0
rediction should be interpreted with caution.;277.28;4.42;81.24;3;3;2;0.474;1.555;
81.7;1;5;6;0.284;1.431;Pass;0
46.34;0.96;114.56;2;6;7;0.438;1.793;Pass;0
s;;433.49;5.04;87.82;1;4;7;0.441;2.367;Fail;1
0.76;Pass;0
C(C)C)c1O;Inactive;0.111;0.516;Yes;;618.77;7.92;152.36;5;8;8;0.187;2.838;Fail;2
;0.568;Yes;;548.01;8.5;39.94;0;4;9;0.186;2.996;Fail;2
;Yes;;476.64;6.1;71.19;2;3;11;0.257;2.853;Fail;1
prediction should be interpreted with caution.;308.29;2.6;91.67;2;4;3;0.85;1.517;
e AD: prediction should be interpreted with caution.;345.4;4.57;62.46;2;3;4;0.681;
.352;No;Outside AD: prediction should be interpreted with caution.;348.35;1.54;13
)c1;Inactive;0.446;0.276;No;Outside AD: prediction should be interpreted with caut
on should be interpreted with caution.;325.39;3.38;57.01;0;6;6;0.513;1.851;Pass;0
;Outside AD: prediction should be interpreted with caution.;362.47;4.25;94.83;3;5;
should be interpreted with caution.;244.29;2.5;46.53;1;3;1;0.825;1.189;Pass;0
e interpreted with caution.;200.24;2.69;40.46;2;2;2;0.782;1.0;Pass;0
6;1;4;12;0.405;2.349;Fail;1
;0.822;1.188;Pass;0
3;0.444;Yes;;444.4;3.97;148.45;2;7;7;0.321;2.389;Pass;0

3;0.399;Yes;;444.4;3.97;148.45;2;7;7;0.321;2.389;Pass;0
ion should be interpreted with caution.;231.21;2.67;83.6;2;4;2;0.614;1.062;Pass;0
4;1;6;8;0.586;1.875;Pass;0
60.44;0;4;3;0.817;1.529;Pass;0
7;Yes;;406.42;4.33;111.15;1;7;6;0.377;2.213;Pass;0
d be interpreted with caution.;214.22;1.75;54.37;1;3;2;0.763;1.028;Pass;0
8;4.02;86.99;3;5;3;0.735;1.581;Pass;0
.39;4.19;75.99;2;5;4;0.637;1.705;Pass;0
.02;7.34;40.46;0;5;8;0.201;2.536;Fail;1
65;7.42;44.76;0;5;14;0.137;2.957;Fail;1
93;64.99;1;5;13;0.308;2.578;Fail;1
0.703;Yes;;449.49;4.43;104.33;0;7;7;0.305;2.399;Pass;0
96;6.46;40.46;0;5;6;0.255;2.28;Fail;1
0.722;1.073;Pass;0
1;4;9;0.541;1.993;Pass;0
;1;3;9;0.702;1.957;Pass;0
o;Outside AD: prediction should be interpreted with caution.;475.59;7.06;57.01;0;5
0.212;No;Outside AD: prediction should be interpreted with caution.;378.47;2.9;104
.6;1;4;3;0.884;1.489;Pass;0
(C)C(=O)C(C)(C)C2=O)C(C)C)c1O;Inactive;0.177;0.591;Yes;;702.84;7.55;186.5;5;10;8;0
3;47.56;1;3;7;0.731;2.047;Pass;0
2;68.65;1;5;12;0.291;2.651;Fail;1
0.892;Pass;0
.347;1.072;Pass;0
uld be interpreted with caution.;242.3;3.98;40.46;2;3;1;0.677;1.185;Pass;0
88;Yes;;415.41;3.85;137.17;2;7;6;0.277;2.231;Pass;0
ctive;0.438;0.345;No;Outside AD: prediction should be interpreted with caution.;51
;1.02;Pass;0
.99;1;5;13;0.362;2.509;Fail;1
;1.277;Pass;0
ctive;0.378;0.316;No;Outside AD: prediction should be interpreted with caution.;54
6.63;44.76;0;5;13;0.192;2.813;Fail;1
.455;0.382;Yes;;490.47;4.29;138.84;1;9;9;0.269;2.681;Pass;0
(C)(C)C(=O)C(C)(C)C1=O;Inactive;0.24;0.482;Yes;;632.79;7.41;124.04;1;8;4;0.269;2.6
6.47;2.1;117.12;4;6;11;0.417;2.333;Pass;0
2.45;1.8;128.12;5;6;10;0.386;2.205;Pass;0
0.398;Yes;;416.39;3.97;131.38;2;7;6;0.359;2.233;Pass;0
34;Yes;;509.7;6.07;84.08;2;5;12;0.227;3.019;Fail;2
e;0.468;0.343;No;Outside AD: prediction should be interpreted with caution.;529.55
.36;55.76;1;3;14;0.313;2.893;Fail;1
1;5;4;0.505;1.128;Pass;0
de AD: prediction should be interpreted with caution.;432.57;3.81;94.07;1;8;2;0.72
43;0.88;132.64;2;9;10;0.331;2.233;Pass;0
6.16;51.46;1;5;5;0.307;2.152;Fail;1
diction should be interpreted with caution.;374.83;2.23;83.48;1;7;4;0.878;1.95;Pas
;Yes;;452.51;4.67;70.42;0;6;7;0.408;2.405;Pass;0
.734;0.295;No;Outside AD: prediction should be interpreted with caution.;556.69;5.
26;0.312;No;Outside AD: prediction should be interpreted with caution.;535.95;4.74
eted with caution.;142.18;2.02;33.37;1;3;0;0.562;0.484;Pass;0
ss;0
9;Yes;;458.9;7.05;61.81;0;5;5;0.264;2.418;Fail;1
;0.199;0.398;Yes;;410.6;7.16;57.53;2;2;6;0.489;2.021;Fail;1
side AD: prediction should be interpreted with caution.;363.8;5.25;51.8;0;4;3;0.47
ction should be interpreted with caution.;294.39;4.83;18.46;0;2;7;0.681;1.689;Pass

```
Na+].[Na+];Active;0.647;0.378;Yes;;594.39;-1.31;83.42;1;5;6;0.218;2.389;Fail;1
22;1.013;Pass;0
;4.66;29.46;1;2;2;0.856;1.333;Pass;0
9;0;3;5;0.644;1.997;Fail;1
.37;0;3;14;0.139;2.065;Fail;1
3;1;1;14;0.225;2.261;Fail;1
1;0;4;2;0.785;1.431;Pass;0
9;0;5;4;0.738;1.625;Pass;0
.97;114.61;1;5;3;0.816;1.601;Pass;0
2;0.775;1.337;Pass;0
63.35;2.17;120.13;1;7;4;0.653;1.727;Pass;0
AD: prediction should be interpreted with caution.;451.35;5.47;80.9;2;5;4;0.404;2.
@]4(C)[C@]3(C)CC[C@@H]12;Active;0.54;0.224;No;Outside AD: prediction should be int
C)[C@]3(C)CC[C@@H]12;Active;0.506;0.214;No;Outside AD: prediction should be interp
;2;4;5;0.753;1.697;Pass;0
;1;11;0.318;1.877;Fail;1
1.46;1;3;2;0.647;1.689;Fail;1
e AD: prediction should be interpreted with caution.;372.45;5.64;45.81;0;4;4;0.556
rediction should be interpreted with caution.;355.44;4.95;50.94;1;4;6;0.532;2.111;
side AD: prediction should be interpreted with caution.;431.54;7.04;39.94;0;4;8;0.
4;2;0.785;1.377;Pass;0
;2;0.851;1.361;Pass;0
.55;Yes;;548.01;8.41;39.94;0;4;8;0.196;2.896;Fail;2
tside AD: prediction should be interpreted with caution.;439.56;7.55;30.71;0;3;6;0
es;;495.63;7.98;39.94;0;4;9;0.204;3.091;Fail;1
o;Outside AD: prediction should be interpreted with caution.;495.63;7.9;39.94;0;4;
(=O)O)[C@@H]5CC[C@]43C)[C@@H]2C1;Inactive;0.421;0.208;No;Outside AD: prediction sh
@]43C)[C@@H]2C1;Inactive;0.435;0.192;No;Outside AD: prediction should be interpret
.81;88.0;2;6;11;0.167;2.59;Fail;1
@]3(C)CC[C@@H]12;Active;0.516;0.187;No;Outside AD: prediction should be interprete
tside AD: prediction should be interpreted with caution.;465.23;2.68;97.74;1;7;2;0
94;88.0;2;6;10;0.178;2.462;Fail;1
C)[C@@H]2C1;Active;0.511;0.19;No;Outside AD: prediction should be interpreted with
@]43C)[C@@H]2C1;Inactive;0.498;0.223;No;Outside AD: prediction should be interpret
3C)[C@@H]2C1;Inactive;0.48;0.179;No;Outside AD: prediction should be interpreted w
C(C)(C)[C@@H]5CC[C@]43C)[C@H]12;Active;0.505;0.217;No;Outside AD: prediction shoul
5CC[C@]43C)[C@H]12;Active;0.571;0.215;No;Outside AD: prediction should be interpre
C@@H]5CC[C@]43C)[C@H]12;Active;0.573;0.22;No;Outside AD: prediction should be inte
]5CC[C@]43C)[C@@H]2C1;Inactive;0.435;0.207;No;Outside AD: prediction should be int
C@@H]5CC[C@]43C)[C@@H]2C1;Inactive;0.481;0.208;No;Outside AD: prediction should be
C[C@]4(C)CC[C@]23C)[C@@]1(C)C(=O)O;Active;0.687;0.278;No;Outside AD: prediction sh
O)[C@@H]5CC[C@]43C)[C@@H]2C1;Inactive;0.474;0.21;No;Outside AD: prediction should
CC[C@]23C)[C@@]1(C)C(=O)O;Active;0.59;0.245;No;Outside AD: prediction should be in
[C@@](C)(CO)[C@@H]5CC[C@]43C)[C@H]12;Active;0.547;0.226;No;Outside AD: prediction
)O)[C@@H]5CC[C@]43C)[C@H]12;Active;0.566;0.248;No;Outside AD: prediction should be
@@H]5CC[C@]43C)[C@@H]2C1;Inactive;0.415;0.204;No;Outside AD: prediction should be
4;5;0.753;1.697;Pass;0
CO)[C@@H]5CC[C@]43C)[C@@H]2C1;Inactive;0.474;0.21;No;Outside AD: prediction should
5CC[C@]43C)[C@@H]2C1;Inactive;0.473;0.219;No;Outside AD: prediction should be inte
C)[C@]3(C)CC[C@@H]12;Active;0.536;0.213;No;Outside AD: prediction should be interp
]5CC[C@]43C)[C@H]12;Active;0.532;0.2;No;Outside AD: prediction should be interpret
;0.868;1.182;Pass;0
38.33;1;2;3;0.919;1.279;Pass;0
ction should be interpreted with caution.;285.34;3.0;38.77;0;3;3;0.633;1.471;Pass;
```

6.3;0;3;13;0.134;2.197;Fail;1
AD: prediction should be interpreted with caution.;393.82;4.43;66.84;1;5;4;0.778;1
rpreted with caution.;154.12;0.8;77.76;3;3;1;0.522;0.608;Pass;0
COCC3)c2)C1.Cl;Inactive;0.205;0.763;Yes;;743.3;5.66;111.9;0;9;12;0.103;3.887;Fail;

[illegible];Fail;1

[illegible];0.594;2.123;Fail;1

11;3.122;Fail;2
)7;0.523;Yes;;1109.04;3.81;389.88;8;28;24;0.018;6.218;Fail;3

22;19;0.027;4.832;Fail;3

'0.75;1;3;5;0.296;2.525;Fail;2
0

37;1;4;6;0.368;2.539;Fail;1

;;7;0.294;2.499;Fail;1

ail;1
28;2.593;Fail;1

caution.;500.62;4.67;95.98;1;8;5;0.596;2.501;Fail;1

);6;0.213;2.702;Fail;2

72.36;2;4;7;0.62;2.747;Pass;0

0

3.058;Fail;2
76.37;1;4;7;0.335;2.667;Fail;1

1.909;Pass;0

5.7;67.15;0;6;7;0.29;2.822;Fail;2

Pass;0
ıss;0

65.29;1;3;6;0.286;2.507;Fail;1

5.41;8.49;100.38;2;4;5;0.217;2.831;Fail;2

';2;0.678;2.309;Pass;0
3;6;0.345;2.441;Fail;1

41;Pass;0
849;Fail;1
ı.;570.68;5.68;153.75;5;9;9;0.189;2.641;Fail;2
0

2;6;0.365;2.439;Fail;1

';65.29;1;3;7;0.261;2.635;Fail;1
Pass;0

ss;0

.68;6.82;144.52;5;8;11;0.15;2.609;Fail;2

;536.62;6.46;133.52;4;8;6;0.309;2.473;Fail;2

;41.37;0;4;7;0.287;2.749;Fail;2
;6;0.33;2.475;Fail;1
;7;0.332;2.567;Fail;1
ution.;570.68;5.79;164.75;6;9;12;0.137;2.741;Fail;3

ass;0

6;79.04;3;4;4;0.252;2.471;Fail;2
;0.187;1.751;Pass;0

3;3;4;5;0.305;2.171;Fail;2
should be interpreted with caution.;512.73;6.84;80.67;1;4;2;0.41;2.225;Fail;2

87;67.15;0;6;7;0.285;2.814;Fail;2
3.118;Fail;3
aution.;572.57;4.05;132.51;1;8;5;0.308;2.845;Fail;1
;7.26;80.12;1;6;10;0.182;3.375;Fail;2

3;6.8;54.26;0;5;7;0.214;3.051;Fail;2

;7;0.239;2.603;Fail;1

611.11;6.58;67.15;0;6;7;0.237;2.922;Fail;2

.;625.74;7.68;54.26;0;5;7;0.186;3.151;Fail;2
0.678;2.179;Pass;0
d be interpreted with caution.;470.69;6.27;74.6;2;3;1;0.474;2.041;Fail;1

'ass;0

22;76.38;0;7;8;0.298;2.905;Fail;2
ss;0

6.97;54.26;0;5;7;0.212;3.044;Fail;2

l with caution.;676.89;9.97;155.52;6;8;16;0.076;3.354;Fail;3

2;0;6;7;0.332;2.693;Fail;1

).684;2.309;Pass;0

359;Pass;0

on.;570.68;5.68;153.75;5;9;10;0.179;2.741;Fail;2
..801;Pass;0
5;0.383;2.311;Fail;1
'ass;0

6;0.472;2.179;Pass;0

n.;536.62;5.85;133.52;4;8;8;0.244;2.673;Fail;2

2;3;6;0.301;2.51;Fail;1

3;1.889;Pass;0
on.;570.68;5.68;153.75;5;9;10;0.179;2.741;Fail;2
n.;570.68;5.68;153.75;5;9;9;0.189;2.641;Fail;2

57;2;5;8;0.331;1.833;Pass;0

.;8;8;0.371;2.597;Pass;0

ail;1
0.703;2.181;Pass;0
;552.66;6.79;133.52;4;8;9;0.197;2.605;Fail;2
54.26;2;3;6;0.274;2.577;Fail;1

3;79.04;3;4;4;0.252;2.438;Fail;2
with caution.;648.7;6.29;160.56;2;9;10;0.129;3.497;Fail;2

738;2.005;Pass;0
5.69;5.93;76.38;0;7;8;0.248;3.013;Fail;2
7;0.251;2.422;Fail;1
426;2.185;Fail;1

Pass;0

Pass;0
1.891;Pass;0
0.61;6;7;4;0.462;1.697;Fail;1
tion.;641.0;7.84;83.91;1;5;7;0.332;2.982;Fail;2

7;0.465;1.625;Pass;0

;7;0.187;2.651;Fail;1
.06;3;6;6;0.485;1.757;Pass;0

.136;3.006;Fail;2

3.51;3.74;140.69;1;8;7;0.291;2.727;Fail;1

0.65;6.52;155.52;6;8;10;0.147;2.481;Fail;3

66;Fail;2

;4.11;132.85;1;9;9;0.251;2.959;Fail;1

5;2.065;Pass;0

s;0

05;76.38;0;7;8;0.302;2.913;Fail;2
;90.21;0;7;7;0.406;2.572;Fail;1

1;1.828;Fail;1
;0

103;Fail;1
:erpreted with caution.;470.69;6.41;74.6;2;3;1;0.464;2.041;Fail;1
oreted with caution.;456.71;6.32;57.53;2;3;1;0.485;2.013;Fail;1

5;1.945;Fail;1
Pass;0
265;2.663;Fail;1

).251;2.679;Fail;1

8;0.215;2.991;Fail;1
hould be interpreted with caution.;502.69;5.27;115.06;4;4;2;0.383;2.205;Fail;2
:ed with caution.;442.73;7.14;40.46;2;2;1;0.424;1.985;Fail;1

ed with caution.;442.73;7.14;40.46;2;2;1;0.424;1.985;Fail;1
).279;1.93;Pass;0

n caution.;426.73;8.17;20.23;1;1;0;0.387;1.853;Fail;1
:ed with caution.;440.71;7.35;37.3;1;2;0;0.427;1.881;Fail;1
vith caution.;442.73;7.14;40.46;2;2;1;0.424;1.985;Fail;1
d be interpreted with caution.;472.71;6.06;77.76;3;3;1;0.405;2.045;Fail;1
eted with caution.;426.73;8.02;20.23;1;1;0;0.389;1.853;Fail;1
erpreted with caution.;456.71;7.09;57.53;2;2;1;0.414;2.013;Fail;1
:erpreted with caution.;472.71;6.21;77.76;3;3;2;0.418;2.145;Fail;1
e interpreted with caution.;486.69;6.3;94.83;3;3;2;0.398;2.173;Fail;1
hould be interpreted with caution.;498.75;7.66;63.6;1;3;2;0.313;2.197;Fail;1
be interpreted with caution.;488.71;5.18;97.99;4;4;2;0.402;2.177;Fail;1
nterpreted with caution.;498.75;7.8;63.6;1;3;2;0.311;2.197;Fail;1
should be interpreted with caution.;488.71;5.03;97.99;4;4;2;0.409;2.177;Fail;1
e interpreted with caution.;456.71;7.09;57.53;2;2;1;0.414;2.013;Fail;1
interpreted with caution.;472.71;6.2;77.76;3;3;1;0.397;2.045;Fail;1

d be interpreted with caution.;488.71;5.18;97.99;4;4;2;0.402;2.177;Fail;1
erpreted with caution.;456.71;7.23;57.53;2;2;1;0.409;2.013;Fail;1
oreted with caution.;456.71;7.23;57.53;2;2;1;0.409;2.013;Fail;1
:ed with caution.;442.73;7.0;40.46;2;2;1;0.429;1.985;Fail;1

0

.. 788;Pass;0

2
